# Identification of a system for hydroxamate xenosiderophore-mediated iron transport in *Burkholderia cenocepacia*

**DOI:** 10.1099/mic.0.001425

**Published:** 2024-01-08

**Authors:** Syakira Mohammed Hussein, Aderonke Sofoluwe, Ameya Paleja, Anne Duhme-Klair, Mark S. Thomas

**Affiliations:** ^1^​ Division of Clinical Medicine, School of Medicine and Population Health, University of Sheffield, Medical School, Beech Hill Road, Sheffield S10 2RX, UK; ^2^​ Department of Immunobiology, School of Immunology & Microbial Sciences, King’s College London, London WC2R 2LS, UK; ^3^​ Department of Chemistry, University of York, Heslington, York YO10 5DD, UK

**Keywords:** iron transport, hydroxamate siderophore, TonB-dependent transporter, periplasmic reductase, sideromycins, *Burkholderia cenocepacia*

## Abstract

One of the mechanisms employed by the opportunistic pathogen *Burkholderia cenocepacia* to acquire the essential element iron is the production and release of two ferric iron chelating compounds (siderophores), ornibactin and pyochelin. Here we show that *B. cenocepacia* is also able to take advantage of a range of siderophores produced by other bacteria and fungi (‘xenosiderophores’) that chelate iron exclusively by means of hydroxamate groups. These include the tris-hydroxamate siderophores ferrioxamine B, ferrichrome, ferricrocin and triacetylfusarinine C, the bis-hydroxamates alcaligin and rhodotorulic acid, and the monohydroxamate siderophore cepabactin. We also show that of the 24 TonB-dependent transporters encoded by the *B. cenocepacia* genome, two (FhuA and FeuA) are involved in the uptake of hydroxamate xenosiderophores, with FhuA serving as the exclusive transporter of iron-loaded ferrioxamine B, triacetylfusarinine C, alcaligin and rhodotorulic acid, while both FhuA and FeuA are able to translocate ferrichrome-type siderophores across the outer membrane. Finally, we identified FhuB, a putative cytoplasmic membrane-anchored ferric-siderophore reductase, as being obligatory for utilization of all of the tested bis- and tris-hydroxamate xenosiderophores apart from alcaligin.

## Introduction

*Burkholderia cenocepacia*, like other members of the *Burkholderia cepacia* complex (Bcc), is a member of the phylum *Pseudomonadota* (formerly *Proteobacteriaceae*) that is found in the rhizosphere and in environments that contain water [1]. However, this species can cause opportunistic respiratory tract infections in immunocompromised patients, particularly in individuals with cystic fibrosis (CF) and chronic granulomatous disease [2, 3]. *B. cenocepacia* possesses strategies to survive under iron-limited conditions in the environment and within the human host, including the ability to utilize haem, an FtrABCD iron uptake system, and the release of ferric iron chelators (siderophores) and their subsequent uptake in the iron-loaded form [4–8]. *B. cenocepacia* produces two siderophores, ornibactin and pyochelin, with ornibactin being the primary siderophore [9]. Ornibactin is a modified tetrapeptide containing two hydroxamate groups and a single hydroxycarboxylate group that facilitates hexadentate chelation of ferric iron, while pyochelin is a metallophore containing two bidentate groups that can coordinate a variety of first-row transition metal ions in addition to ferric iron (see Thomas [9] for a review).

The first step in the uptake of iron-loaded siderophores by Gram-negative bacteria is translocation of the chelate across the outer membrane by means of a TonB-dependent transporter (TBDT). TBDTs are 22-stranded pore-like β-barrel proteins in which the N-terminal region of the polypeptide forms a ‘plug’ domain that is inserted into the lumen of the barrel [10, 11]. Binding of the cognate ferric-siderophore complex to external loops of the barrel and the plug domain triggers conformational changes that result in unfolding or displacement of the plug domain, thereby allowing entry of the iron-siderophore complex to the periplasmic space. The energy required to elicit these conformational changes derives from the proton motive force generated at the cytoplasmic membrane and requires an interaction between a conserved N-terminal sequence of 5–7 aa (the ‘TonB box’) that is located on the periplasmic side of the plug domain and the C-terminal domain of the cytoplasmic membrane-anchored TonB protein [12, 13]. Exogenous ferric-ornibactin is transported into the periplasmic compartment of *B. cenocepacia* via the TBDT, OrbA, whereas uptake of iron-loaded pyochelin has been shown to require the FptA TBDT in *Pseudomonas aeruginosa* and *Burkholderia pseudomallei* [14–16]. Based on studies carried out in *P. aeruginosa*, pyochelin appears to be transported from the periplasmic space into the cytosol by a single subunit cytoplasmic membrane permease, FptX [17–19]. Genes encoding presumed FptA and FptX orthologues (BCAM2224 and BCAM2221, respectively) are also present in the pyochelin gene cluster of *B. cenocepacia* [9].

Many bacteria can also use siderophores released by other micro-organisms (xenosiderophores). Utilization of xenosiderophores by Gram-negative bacteria requires additional sets of transport proteins. For example, *P. aeruginosa* strain PAO1, which produces the siderophores pyoverdine and pyochelin, possesses approximately 35 TBDTs [20]. Two of these are the ferric-pyoverdine and ferric-pyochelin TBDTs, FpvA and FptA, respectively, but many of the other TBDTs are involved in the uptake of iron–xenosiderophore complexes [21–26]. These include the FoxA (PA2466) and FiuA (PA0470) TBDTs, which can transport ferrioxamine B and ferrichrome, respectively, and FpvB (PA4168), which can transport both xenosiderophores [22, 23, 27, 28]. Possession of uptake systems for exogenously produced siderophores offers the bacterium a potential advantage, as it saves the metabolic energy needed for synthesizing its own endogenous siderophore for survival during iron scarcity [29]. However, possession of an uptake system for a xenosiderophore can also render the bacterium susceptible to naturally occurring siderophore mimics that possess a toxic moiety. Such compounds (known as sideromycins) include the ferrichrome mimic, albomycin δ_2_, and the ferrioxamine B mimic, salmycin A, both of which are protein synthesis inhibitors that are produced by *Streptomyces* species [30].

Ferrioxamine B and ferrichrome are examples of hydroxamate-type siderophores (siderophores that exclusively chelate iron through one or more hydroxamate groups) (Fig. S1, available in the online version of this article). Hydroxamate siderophores are among the most common secondary metabolites secreted by bacteria and fungi, including almost all soil fungi [31, 32]. Given that *B. cenocepacia* can exploit niches that are occupied by several other micro-organisms, it is likely that this bacterium can utilize exogenously produced hydroxamate siderophores for survival. To test this hypothesis, different types of hydroxamate siderophore of both bacterial and fungal origin were screened for their ability to support growth of *B. cenocepacia* under iron-restricted conditions. We observed that *B. cenocepacia* can utilize a broad spectrum of exogenously produced hydroxamate siderophores. Moreover, we identified the TBDTs that are required for the transport of some members of this class of siderophore and showed that utilization of tris-hydroxamate xenosiderophores depends on a cytoplasmic membrane protein that is likely to promote reductive release of iron from the ferric–siderophore complex in the periplasm. We propose that this mode of siderophore utilization was adopted by *B. cenocepacia* for protection from sideromycins that target essential cytoplasmic functions.

## Methods

### Bacterial strains, media and growth conditions

Bacterial strains are listed in Table S1. *B. cenocepacia* strains were grown at 37 °C and were maintained at room temperature on M9-glucose agar. *Escherichia coli* strains were cultured at 37^ ^°C (except where indicated otherwise) and maintained on LB agar at 4 °C. For routine culture of bacteria, Lysogeny broth (LB) was used, solidified with the addition of agar (1.5 %, w/v) for plates [33]. Lennox agar was prepared as for LB agar but with half the amount of NaCl added. M9 minimal salts broth was prepared as previously described [34] and also contained 0.5 % (w/v) glucose (M9-glucose). M9-CAA was M9-glucose supplemented with 0.1 % casamino acids (Becton-Dickinson). To generate iron-replete conditions, ferric chloride was added to the medium to a final concentration of 50 µM. When required, M9-glucose agar solidified with Agar No. 1 (Oxoid) was supplemented with 0.1 % (w/v) dl-4-chlorophenylalanine (cPhe; Acros Organics) prior to autoclaving [35]. Selection for *E. coli* transformants containing pSNUFF derivatives was performed on M9 agar containing 0.5 % glycerol as the carbon source, 5 μg ml^−1^ thiamine, 40 µg ml^−1^ X-gal, 0.1 mM IPTG and 25 μg ml^−1^ trimethoprim at 30 °C. Otherwise, Iso-Sensitest (IST) medium was used for selection and maintenance of trimethroprim-resistant plasmids in *E. coli* strains. *E. coli* transformants containing pSNUFF3Cm derivatives were selected for on LB agar containing chloramphenicol (25 μg ml^−1^), X-gal and IPTG. *B. cenocepacia* strains expressing trimethoprim resistance were maintained on M9-glucose agar containing 25 μg ml^−1^ trimethoprim, although IST agar containing 25 μg ml^−1^ trimethoprim was employed when scoring for the presence or absence of chromosomally integrated pSHAFT-GFP derivatives by viewing colonies under UV light [36]. *B. cenocepacia* strains containing pSRKKm plasmid derivatives were maintained on M9-glucose agar containing 50 μg ml^−1^ kanamycin.

### Recombinant DNA techniques

Boiled lysates were used as templates for PCR amplification from bacterial genomic DNA. For DNA amplification prior to cloning, Q5 High-fidelity DNA polymerase was used. Following PCR amplification of genomic DNA or restriction digestion, DNA fragments were purified using the GeneJet PCR Purification Kit or GeneJET Gel Extraction Kit (Thermo Scientific) according to the manufacturer’s instructions. Following restriction digestion, the enzyme was inactivated and buffer components were removed using the PCR Purification Kit (Thermo Scientific). Transformation of *E. coli* cells with plasmid DNA was performed using the heat shock method [37]. Successful ligation was determined by colour screening of transformant colonies on selective medium containing X-gal (40 µg ml^−1^) and IPTG (100 µM) for pEX18Tp-pheS, pBBR1MCS, pSNUFF and pSRKKm derivatives, and by PCR screening using DreamTaq (Promega) or GoTaq G2 Flexi (NEB) DNA polymerases.

The integrity of cloned DNA was confirmed by Sanger DNA sequencing carried out by the Medical School Genetic Core Sequencing Facility at the University of Sheffield. The M13for and M13rev primers were used for reading DNA inserted into the multiple cloning site (MCS) of the plasmids pBBR1MCS and pSRKKm. M13for and M13BACTHrev primers were used for sequencing DNA cloned into pEX18Tp-pheS-derived plasmids, while catendout and pUTcatrev primers were used for pSHAFT derivatives. Additional primers that annealed to the cloned DNA were also used where necessary to close any gaps in the sequence.

### Construction of plasmids

Plasmids and oligonucleotides used in this study are shown in Tables S2 and S3, respectively. pSNUFF is a derivative of the allelic replacement vector pEX18Tp-pheS [35] in which the tandem *rrnB* terminators have been removed and the *dfrB2* (Tp^R^) gene has been replaced by the TpTer cassette from p34E-TpTer [36, 38]. We have found that pSNUFF is much more efficient than pEX18Tp-pheS for introducing mutant alleles into the *B. cenocepacia* genome (Spiewak and Thomas, unpublished results). pSNUFF3Cm additionally contains the *cat* (Cm^R^) gene to facilitate selection of recombinants containing the chromosomally integrated vector on nutrient agar. It also harbours the target site for the yeast I-SceI meganuclease that allows for resolution of plasmid integrants upon introduction of a I-SceI expression plasmid (Spiewak and Thomas, unpublished results).

To generate pSNUFF-ΔpobA, a 2010 bp DNA fragment containing the *B. cenocepacia pobA* gene (756 bp) and flanking genomic DNA was amplified using primers pobAfor2 and pobArev2 and the amplicon was ligated to the *Bam*HI and *Hin*dIII sites of pSNUFF to generate pSNUFF-pobA. A 399 bp in-frame deletion was then introduced into the *pobA* coding sequence by cleavage of pSNUFF-pobA with *Sac*II followed by self-ligation of the plasmid, resulting in pSNUFF-ΔpobA. To generate pSNUFF-ΔfeuA, the *feuA* gene together with 300–400 bp of flanking DNA was amplified as a 2916 kb fragment with primers BCAL2281for and BCAL2281rev, whereupon the resulting product was cut with *Acc*65I and *Hin*dIII, and ligated to the corresponding sites of pSNUFF giving rise to pSNUFF-feuA. Inactivation of *feuA* by in-frame deletion was carried out by digestion of this plasmid with *Sna*BI and *Zra*I, which released a 981 bp fragment from the *feuA* gene, followed by self-ligation to give pSNUFF-ΔfeuA. pSNUFF3Cm-ΔfhuB was constructed by amplifying a 1956 bp DNA fragment containing the entire *fhuB* gene (1236 bp) and flanking sequences with primers BCAL0117for2 and BCAL0117rev3. The amplicon was ligated to the *Bam*HI and *Eco*RI sites of pSNUFF3Cm to generate pSNUFFCm-fhuB. This plasmid was passaged through the *E. coli dam* mutant, GM48, following which an 879 bp in-frame deletion was introduced into the FhuB coding sequence by digestion with *Nru*I followed by self-ligation, giving rise to pSNUFF3Cm-ΔfhuB. pSHAFT-GFP-ΔfhuA was constructed in two steps by amplification of a 1263 bp DNA fragment internal to the *fhuA* gene with primers BCAL0116for and BCAL0116rev, digestion of the resulting amplicon with *Xho*I and *Xba*I, and ligation to the corresponding sites of pSHAFT-GFP, resulting in pSHAFT-GFP-fhuA. The TpTer cassette was then removed from p34E-TpTer as an *Sma*I fragment and transferred into the *Zra*I site located within the FhuA coding sequence contained on pSHAFT-GFP-fhuA.

To construct pSHAFT2-ΔorbA, a large region of the *orbA* gene was amplified with primers BCAL1700for and BCAL1700rev, and the resulting amplicon (1780 bp) was digested with *Hin*dIII and *Xba*I, and cloned between the same sites of pBBR1MCS forming pBBR1-orbA. The cloned *orbA* DNA was then disrupted by insertion of the *Bam*HI TpTer cassette (acquired from p34E-TpTer) between the pair of *Bam*HI sites located within *orbA*, giving rise to pBBR1-ΔorbA. Restriction digestion of the plasmid with *BsrG*I and *Xba*I showed that the *dfrB2* gene was inserted in the same orientation as the *orbA* coding sequence. In the final step, a DNA fragment containing the *orbA* mutant allele was released from pBBR1-ΔorbA by digestion with *Bgl*II and *Xba*I, and was ligated to pSHAFT2 digested with the same enzymes.

To construct pSRKKm-fhuA, the entire *fhuA* gene was amplified with primers BCAL0116forfull and BCAL0116revfull. The 2319 bp amplicon was digested with *Hin*dIII and *Bam*HI, and ligated to pSRKKm which was cut with the same restriction endonucleases, giving rise to pSRKKm-fhuA. Note that the forward primer contained a stop codon to terminate readthrough translation of *lacZ* mRNA, thereby preventing the production of a LacZ–FhuA fusion protein and possible occlusion of the *fhuA* translation initiation region. To construct pSRKKm-feuA, the same PCR product used to generate pSNUFF-feuA was digested by *Hin*dIII and *Acc*651, and ligated between the same restriction sites of pSRKKm. pSRKKm-fhuB was constructed by amplifying the *fhuB* gene with primers BCAL0117for and BCAL0117rev2, and ligating the 1348 bp product to the *Bam*HI and *Sal*I sites of pSRKKm.

### Conjugation of plasmid DNA into *B. cenocepacia*

Allelic replacement and complementation plasmids were introduced into *B. cenocepacia* by conjugation using S17-1(λpir) as the *E. coli* donor strain except for plasmids expressing only trimethoprim resistance, in which case SM10(λpir) was used. Conjugal transfer of plasmids was carried out by the biparental filter mating procedure as previously described [39, 40]. To isolate *B. cenocepacia* exconjugants containing pSRKKm-derived complementation plasmids, Lennox agar containing kanamycin (100 µg ml^−1^) was used as the selection medium, with the addition of tetracycline (10 µg ml^−1^) to counterselect the *E. coli* donor strain.

### Construction of *B. cenocepacia* mutants

The markerless *B. cenocepacia pobA* mutant was generated following introduction of pSNUFF-ΔpobA into *B. cenocepacia* strain H111 by conjugation and selection for *B. cenocepacia* exconjugants on M9-glucose agar containing trimethoprim (25 μg ml^−1^). Plasmid integrants that arose through recombination between homologous sequences carried by the plasmid and the target genome were verified by PCR with primers pEX18Tpfor and pEX18Tprev. Merodiploids were restreaked on counter-selective medium containing cPhe and lacking trimethoprim. cPhe-resistant recombinants that were trimethoprim-sensitive were checked by PCR for the presence of the mutant allele at the expected locus using primers pobAfor4 and pobArev3. The Δ*feuA* allele was introduced into the H111ΔpobA strain in an analogous fashion using pSNUFF-ΔfeuA, and following resolution of merodiploids diagnostic PCR with primers BCAL2281forout2 and BCAL2281revout was carried out to identify Δ*feuA* mutants.

To generate the *fhuB* mutant, plasmid pSNUFF3Cm-ΔfhuB was introduced into *B. cenocepacia* H111 by conjugation followed by selection for *B. cenocepacia* exconjugants on LB agar containing chloramphenicol (50 μg ml^−1^). Following confirmation of genomic integration of the plasmid, a second plasmid, pDAI-SceI-pheS, was then similarly introduced into the strain to promote a second homologous recombination event, thereby resolving the merodiploid state. *B. cenocepacia* exconjugants containing pDAI-SceI-pheS were selected on Lennox agar containing tetracycline (125 μg ml^−1^) and ampicillin (100 μg ml^−1^). Chloramphenicol-/trimethoprim-sensitive exconjugants were screened by PCR with vector-specific primers to confirm loss of the integrated plasmid and with BCAL0117forout and BCAL0117revout to identify Δ*fhuB* mutants. One such mutant was subsequently cured of the I-SceI expression plasmid by growing an overnight culture in the absence of antibiotic selection, washing the cells twice with an equal volume of 0.85 % (w/v) saline and resuspending them in 0.5 vol saline. Then 100 µl of the bacterial cell suspension was spread on M9-glucose agar containing cPhe, and cPhe-resistant colonies were screened for loss of tetracycline resistance.

To generate *fhuA* mutants, pSHAFT-GFP-ΔfhuA was introduced into the appropriate *B. cenocepacia* strain by conjugation followed by selection for trimethoprim-resistant exconjugants on M9-CAA agar containing trimethoprim (25 μg ml^−1^) and tetracycline (10 μg ml^−1^). Candidate double-crossover recombinants that resulted in replacement of the *fhuA* gene by the Δ*fhuA* (*fhuA*::TpTer) allele were identified by their lack of GFP fluorescence under UV light [36]. PCR with primers BCAL0116forout and BCAL0116revout was then used to confirm the presence of the Δ*fhuA* allele.

H111ΔpobAΔorbA was generated by conjugating the *E. coli* donor strain SM10(λpir) containing pSHAFT2-ΔorbA with H111ΔpobA and selecting exconjugants on M9-CAA agar containing tetracycline (10 µg ml-^1^) and trimethoprim (25 µg ml^−1^). Exconjugants that were unable to grow on M9-CAA agar containing chloramphenicol (50 µg ml^−1^) were screened by PCR using primers BCAL1700forout and BCAL1700revout to confirm the allelic replacement of the wild-type *orbA* gene by the constructed Δ*orbA* allele.

### Xenosiderophore utilization bioassays

All siderophores were used as iron-free purified products except for ferricrocin which was iron-loaded. Ferrichrome and ferrioxamine B were obtained from Sigma-Aldrich. Rhodotorulic acid and triacetylfusarinine C were obtained from EMC Microcollections. Alcaligin, cepabactin and ferricrocin were generous gifts from T. Brickman (University of Minnesota), G. Mislin (Université de Strasbourg) and J. Coulton (McGill University), respectively. All siderophores were dissolved in iron-free water and were made into stock solutions (5 mM).

Siderophore utilization assays conducted on solid medium were performed using a modified disc diffusion method [41, 42]. For this, 100 µl of an aerated iron-enriched overnight culture of the bacterial test strain was mixed with 3 ml of 0.65 % LB soft agar maintained at 42 °C and then overlaid as a thin layer onto an LB agar plate supplemented with the ferric iron chelator, ethylene diamine-*N*,*N′*-bis(2-hydroxyphenylacetic acid) (EDDHA, 40 µM). Siderophore solutions (10–20 µl) were spotted onto 13 mm paper discs (Whatman AA) which were then placed onto the solidified bacterial overlay and the plates were incubated inverted at 37 °C for 48 h. Haloes of bacterial growth around the filter disc were indicative of siderophore utilization. Complementation analysis was performed by the same method with inclusion of 1–5 mM IPTG in the soft agar overlay to induce transcription of the inserted gene in the pSRKKm plasmid.

Xenosiderophore utilization was assessed in liquid culture by performing growth curves in iron-restricted medium. A single colony of each *Burkholderia* strain to be tested was cultured for 24–48 h in M9-CAA medium [supplemented with kanamycin (50 µg ml^−1^) for strains harbouring a pSRKKm derivative]. A 50 ml aliquot of the same medium containing the ferric iron chelator diethylenetriamine pentaacetic acid (DTPA, 1 µM) was inoculated with the overnight culture to an initial optical density (OD) at 600 nm of 0.010–0.011 (approximately 1 : 250 dilution of the overnight culture). The test siderophore was included in the medium at 10 µM and IPTG (5 mM) was also added for complementation analysis. Cultures were grown with aeration at 37 °C and the bacterial growth was measured by monitoring OD_600_ at 1 h intervals for up to 10 h.

### Statistical analysis

Growth curves represent the means±sd of at least three independent experiments. Growth rates (doublings per hour) were determined from the exponential growth phase and comparisons were made using an unpaired Student’s t-test (two-tailed). Differences with a *P*-value <0.05 were considered significant. Disc diffusion assays were performed on at least three occasions and representative results are shown.

## Results

### *B. cenocepacia* can utilize hydroxamate xenosiderophores

**Fig. 1.**
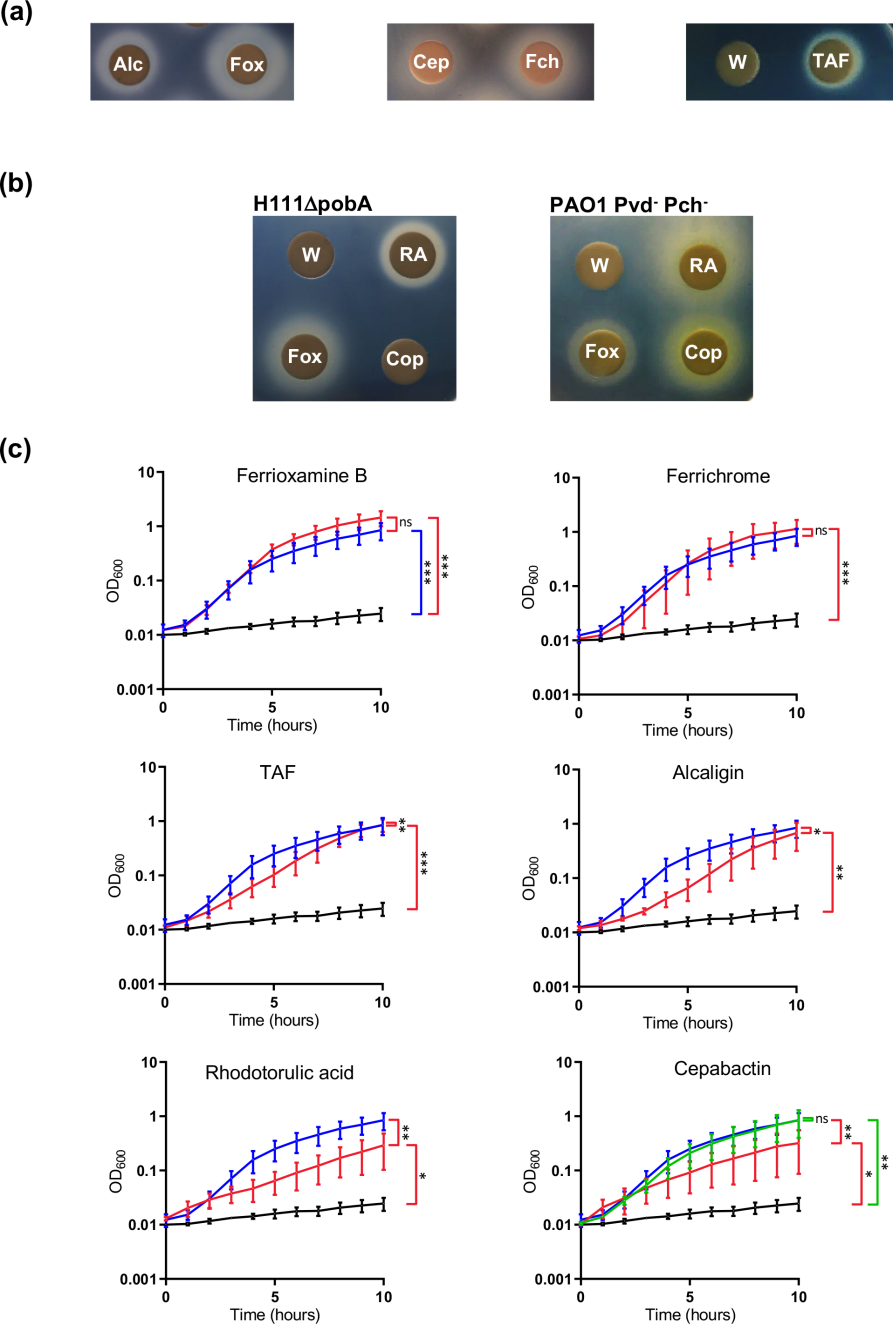
Growth promotion of *B. cenocepacia* by hydroxamate siderophores under iron-limiting conditions. (**a**) Analysis of alcaligin (Alc), cepabactin (Cep), ferrichrome (Fch), ferrioxamine B (Fox) and triacetylfusarinine C (TAF) utilization by *B. cenocepacia* H111ΔpobA using the disc diffusion assay. The negative control was iron-free water (W). (**b**) Analysis of coprogen (Cop) and rhodotorulic acid (RA) utilization by *B. cenocepacia* H111ΔpobA and *P. aeruginosa* PAO1 Pvd^-^ Pch^-^ using the disc diffusion assay. (**c**) Growth curves of *B. cenocepacia* in iron-depleted medium supplemented with different xenosiderophores. *B. cenocepacia* H111ΔpobA was grown at 37 °C in M9-CAA medium containing DTPA and supplemented with the indicated siderophores. Growth in the presence of the test siderophore (red growth curve) is compared to the same strain growing in medium without siderophore added (black growth curve) and in medium containing ornibactin (blue growth curve). Siderophores were included in the medium at 10 µM and additionally at 30 µM for cepabactin (green growth curve). Growth rates (doublings per hour) for media supplemented with the following siderophores were: ornibactin, 1.11±0.11; ferrioxamine B, 1.21±0.18; ferrichrome, 1.17±0.16; triacetylfusarinine C, 0.75±0.05; alcaligin, 0.73±0.18; rhodotorulic acid, 0.47±0.13; cepabactin (30 µM), 1.00±0.24; cepabactin (10 µM), 0.50±0.14; and for no siderophore: 0.19±0.06. Data represent the means of three independent experiments (*n*=3). Error bars, mean±sd. ***P<0.001, **P<0.01, *P<0.05, ns=not significant.

We have previously shown that inactivation of the *B. cenocepacia pobA* gene, encoding an Sfp-type phosphopantetheinyl transferase (PPTase), abolishes production of the two endogenous siderophores, ornibactin and pyochelin [43]. Therefore, to facilitate our investigation into the ability of *B. cenocepacia* to utilize hydroxamate xenosiderophores, we constructed an unmarked *pobA* mutant derivative of strain H111 (H111ΔpobA) and confirmed that, like the previously obtained *pobA* transposon mutant, AHA27, it was unable to produce endogenous siderophores (Fig. S2a). As expected, and in contrast to the wild-type strain, growth of the *pobA* mutant in medium containing the ferric iron chelator DTPA was almost completely abolished (Fig. S2b). The mutant strain was then used to screen for the ability of *B. cenocepacia* to utilize exogenously supplied hydroxamate siderophores using a disc diffusion assay. The siderophores tested included representatives possessing one (cepabactin), two (alcaligin and rhodotorulic acid) or three [ferrichrome, ferrioxamine B, triacetylfusarinine C (TAF) and coprogen] hydroxamate ligands (Fig. S1). The results of the disc diffusion assay showed that *B. cenocepacia* can utilize all of the tested hydroxamate siderophores except for coprogen (Fig. 1a, b). As expected, coprogen and rhodotorulic acid promoted the growth of a siderophore-deficient strain of *P. aeruginosa* which is able to use these siderophores (Fig. 1b) [44].

The ability of the hydroxamate siderophores to stimulate growth of the *B. cenocepacia pobA* mutant in iron-restricted broth medium was also analysed. Addition of the xenosiderophores promoted growth of the *pobA* mutant with varying efficiency in broth culture. The ferrichrome and ferrioxamine B-stimulated growth rates were similar to the ornibactin-stimulated growth rate (Fig. 1c). In the presence of TAF or alcaligin, the *pobA* mutant exhibited a lower growth rate than when supplemented with ornibactin but it achieved the same final optical density as the ornibactin-supplemented culture (Fig. 1c). Rhodotorulic-acid- and cepabactin-supplemented cultures also grew more slowly than when ornibactin was present in the medium and did not attain the same final cell density as observed for the other siderophore-supplemented cultures over the time course of the experiment (Fig. 1c). However, when the concentration of cepabactin was increased threefold, so that the concentration of the bidentate ligand was the same as present in the ornibactin-supplemented culture, this resulted in stimulation of the growth rate of the *pobA* mutant to a level similar to that promoted by ornibactin (Fig. 1c). Together, these results suggest that all of the hydroxamate siderophores tested, except for coprogen, can be utilized by *B. cenocepacia* to scavenge iron.

### Identification of a TBDT for hydroxamate xenosiderophore utilization in *B. cenocepacia*

**Fig. 2.**
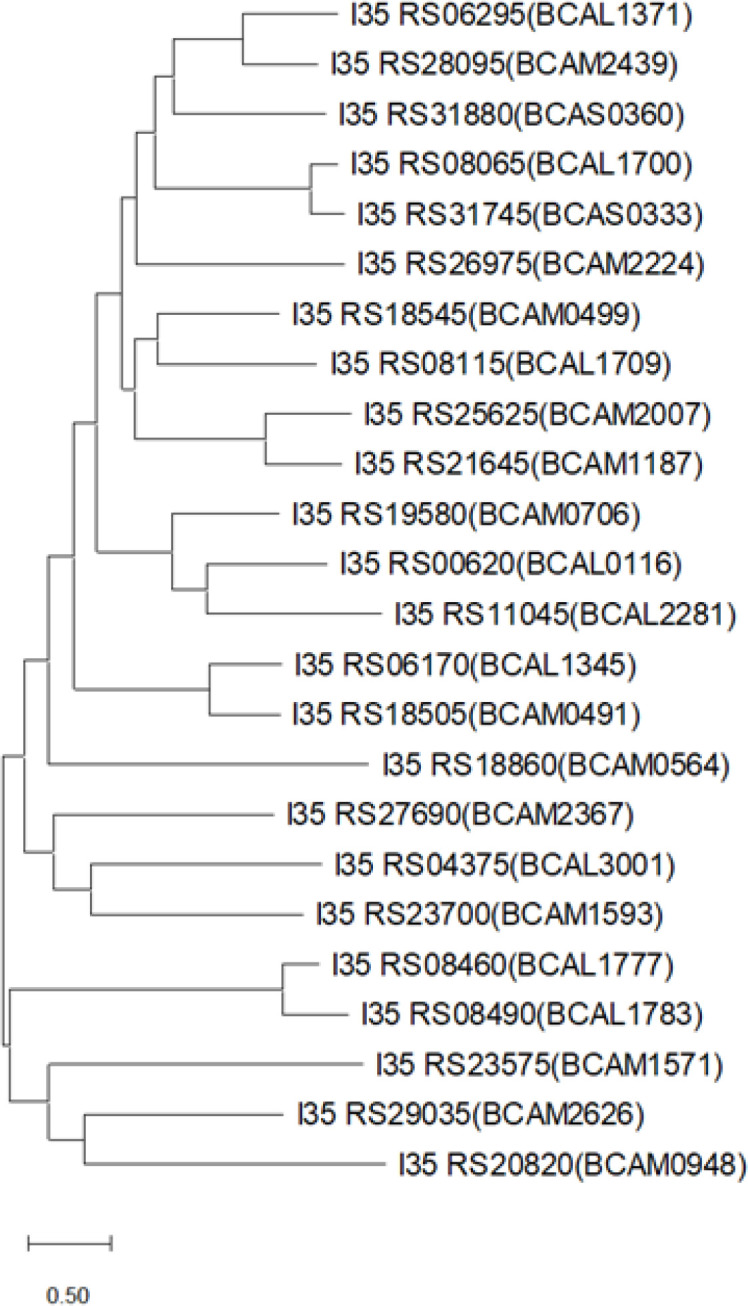
Phylogeny of TBDTs encoded by *B. cenocepacia*. Maximum-likelihood phylogenetic tree reconstructed from amino acid sequences of 24 TBDTs encoded by *B. cenocepacia* strain H111. Corresponding TBDTs in strain J2315 are shown in parentheses. Sequences are of the mature proteins and extend from the TonB box to the C-terminus in each case (see Fig. S3). The alignment and tree reconstruction were performed using mega 11. Bar, 0.5 substitutions per site. Note that the genes encoding the J2315 BCAL1783 and BCAM0706 TBDTs contain an in-frame translation stop codon and a frameshift mutation, respectively.

Having demonstrated that *B. cenocepacia* can utilize various hydroxamate xenosiderophores, we sought to identify the TBDT(s) involved in transporting them into the periplasm. The translated genomes of *B. cenocepacia* strains H111 and J2315 were subjected to a blastp search using two known hydroxamate siderophore TBDTs as search queries, the PA0470 (FiuA) and PA2466 (FoxA) TBDTs of *P. aeruginosa* PAO1. To increase the likelihood that all the *B. cenocepacia* TBDTs would be identified, a few of the most poorly matching *B. cenocepacia* TBDTs were subsequently used as queries in successive blastp searches. By this means, 24 TBDTs were identified in H111, with the majority being encoded on chromosome 2 (Fig. 2 and Table S4). Genes encoding these TBDTs were also identified in strain J2315, although in two cases, the gene was inactivated through mutation (Table S4). As expected, sequences matching the TonB box and the plug domain were identified near the N-terminus of each mature protein (Fig. S3 [45]). Unexpectedly, the best matching *B. cenocepacia* TBDT to both of the *P. aeruginosa* search queries was OrbA (BCAL1700), the ornibactin TBDT (gene locus nomenclature of the *B. cenocepacia* type strain, J2315, is used in this report). Although ornibactin contains two hydroxamate groups, OrbA does not serve as an outer membrane transporter for at least some hydroxamate siderophores, as an *orbA* mutant can utilize ferrioxamine B for iron acquisition (Fig. S4). Therefore, we reviewed the genomic context of the *B. cenocepacia* TBDT genes. We observed that although the TBDT encoded by the BCAL0116 gene locus, here referred to as FhuA, was not among the ten best matching *B. cenocepacia* TBDT candidates to either of the search queries, the gene located immediately upstream of *fhuA* (BCAL0117, here referred to as *fhuB*) (Fig. S5) encodes a protein that is homologous to FoxB (PA2465), which is encoded adjacent to the *foxA* gene in *P. aeruginosa*. The product of *foxB* was originally proposed to serve as the cytoplasmic membrane transporter of ferric-ferrioxamine B but more recent evidence suggests that it functions as a periplasmic reductase that facilitates release of iron from ferric-ferrioxamine E ([46, 47] see Discussion).

**Fig. 3.**
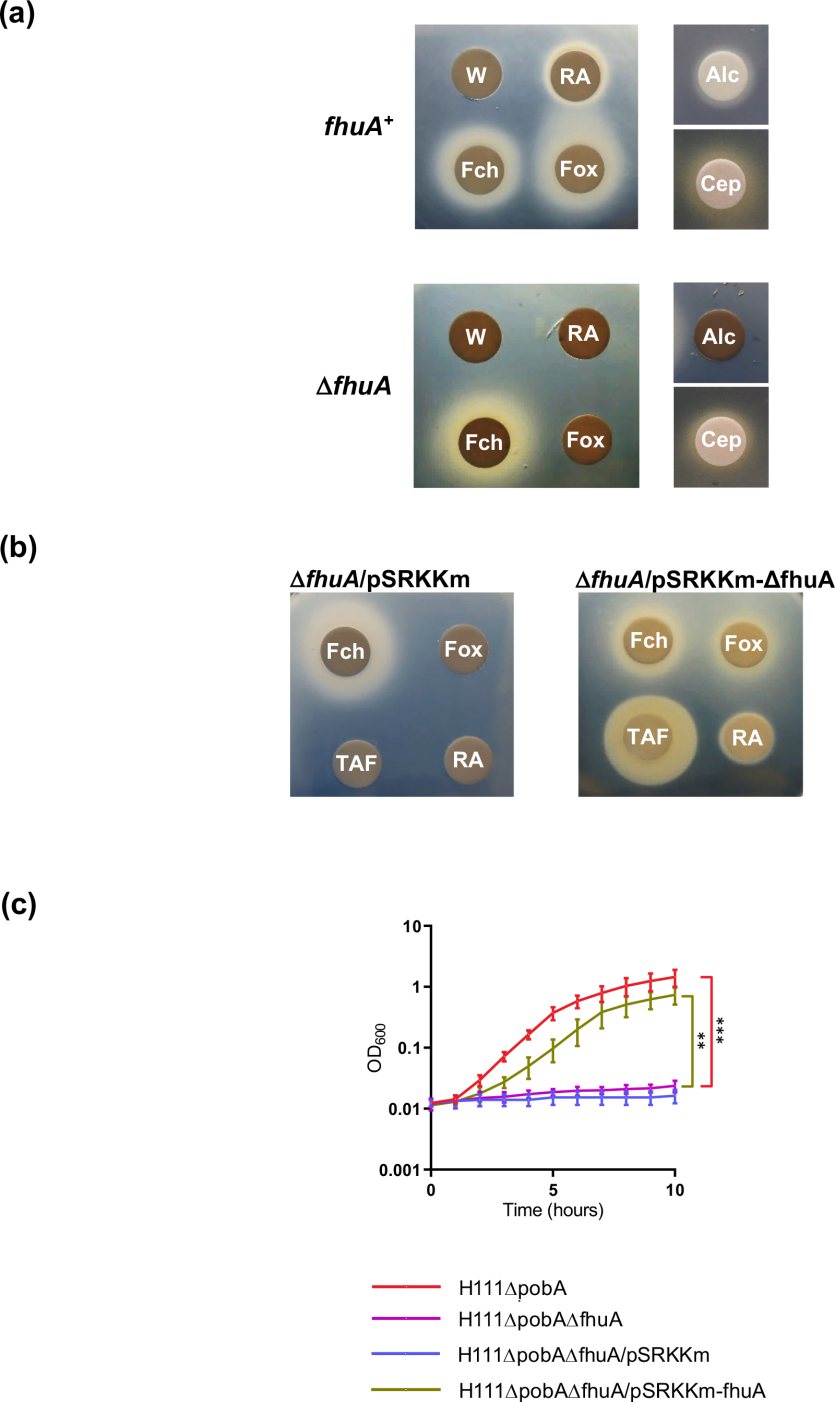
Effect of inactivation of *fhuA* on hydroxamate siderophore utilization by *B. cenocepacia*. (**a**) Analysis of alcaligin (Alc), cepabactin (Cep), ferrichrome (Fch), ferrioxamine B (Fox) and rhodotorulic acid (RA) utilization by *B. cenocepacia* H111ΔpobAΔfhuA (Δ*fhuA*) compared to the H111ΔpobA parent strain (*fhuA^+^*) using the disc diffusion assay. Deionized water (W) was used as a negative control. (**b**) Complementation analysis of the Δ*fhuA* mutant using the disc diffusion assay. Filter discs spotted with ferrichrome, ferrioxamine B, triacetylfusarinine C (TAF) or rhodotorulic acid were applied to assay plates seeded with the H111ΔpobAΔfhuA mutant containing plasmid pSRKKm-fhuA or the empty vector, pSRKKm, as indicated. (**c**) Growth of the H111ΔpobAΔfhuA mutant in iron-depleted M9-CAA broth medium supplemented with DTPA and 10 µM ferrioxamine B compared to the *fhuA^+^* parent strain. Also shown is growth of the H111ΔpobAΔfhuA mutant containing plasmid pSRKKm-fhuA or the empty vector, pSRKKm. Data points represent the means of three independent experiments (*n*=3). Error bars, ±sd. ***P<0.001, **P<0.01.

To investigate the possibility that FhuA could transport hydroxamate siderophores into *B. cenocepacia*, we introduced a *fhuA* null allele into the *B. cenocepacia pobA* mutant and tested the resulting strain (H111ΔpobAΔfhuA) for the ability to utilize hydroxamate siderophores. The disc diffusion assay showed that inactivation of *fhuA* prevented the utilization of ferrioxamine B, TAF, rhodotorulic acid and alcaligin by *B. cenocepacia*, indicating that the *fhuA* gene product is likely to be solely responsible for transporting these iron-loaded siderophores across the outer membrane of *B. cenocepacia* (Fig. 3a). In contrast, the mutant was still able to utilize ferrichrome and cepabactin. Introduction of a plasmid expressing *fhuA* into the mutant led to restoration of growth promotion by ferrioxamine B, TAF, rhodotorulic acid and alcaligin (Fig. 3b).

To provide further evidence that FhuA represents the sole TBDT for some hydroxamate xenosiderophores, the effect of ferrioxamine B on growth of the *pobA fhuA* mutant was monitored in iron-depleted liquid medium. Under these conditions, growth of the mutant was almost completely abolished (Fig. 3c). As expected, growth of the *pobA fhuA* mutant containing the *fhuA* complementation plasmid was significantly stimulated by ferrioxamine B (Fig. 3c). These results confirmed the role of FhuA in utilization of ferrioxamine B. Assignment of the name FhuA (ferric hydroxamate utilization protein A) to this protein was based on its ability to facilitate growth stimulation by several different hydroxamate siderophores, although it should be noted that the *E. coli* FhuA TBDT is more specific for ferrichromes [48].

### Identification of a second TBDT for hydroxamate xenosiderophores in *B. cenocepacia*

**Fig. 4.**
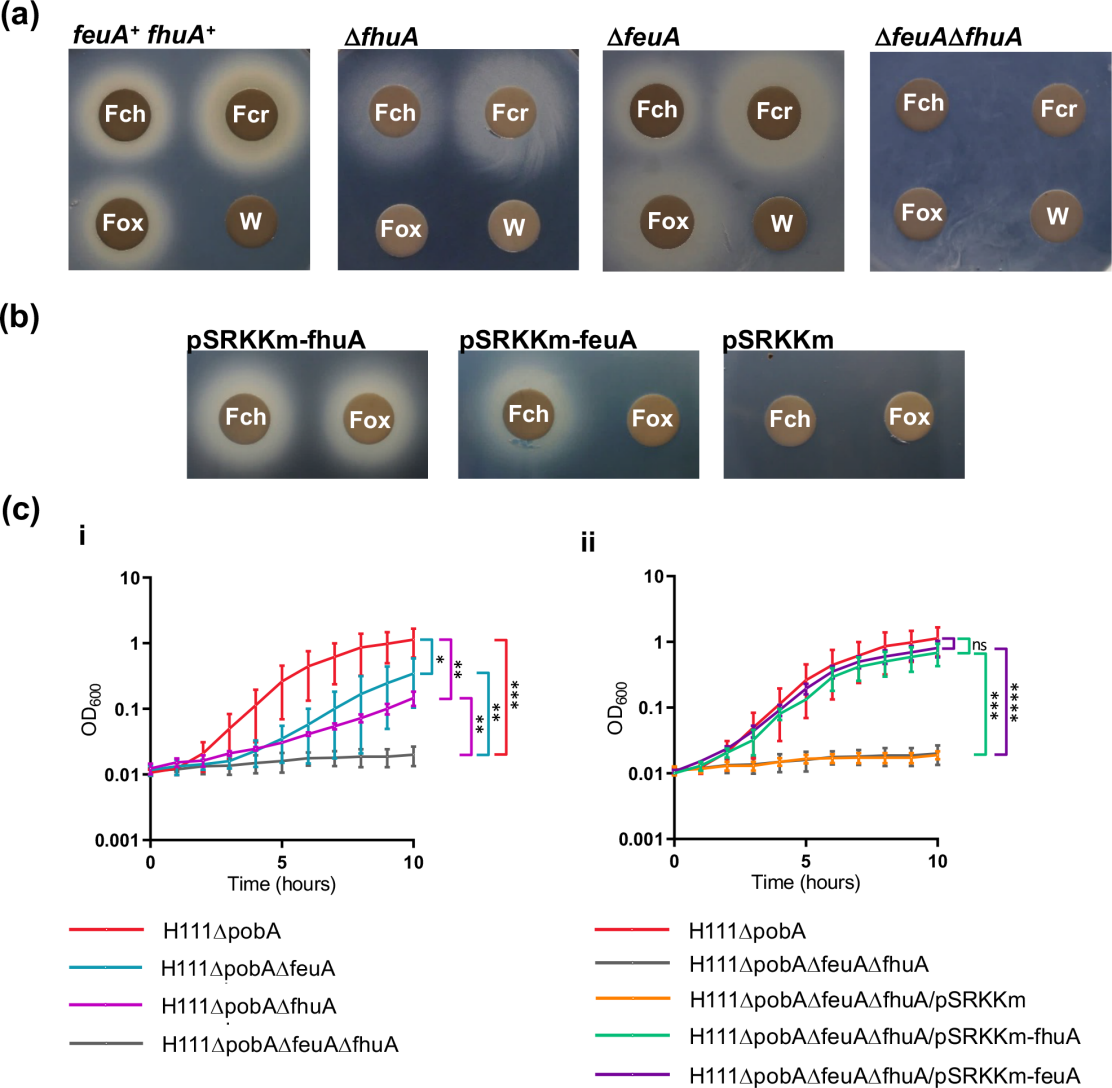
Effect of inactivation of *feuA* on hydroxamate siderophore utilization in the absence or presence of an *fhuA* null allele. (**a**) Analysis of ferrichrome (Fch) and ferricrocin (Fcr) utilization by the Δ*feuA* and Δ*feuA*Δ*fhuA* mutants compared to the H111ΔpobA parent strain (*feuA* ^+^ *fhuA* ^+^) and the Δ*fhuA* mutant using the disc diffusion assay. Ferrioxamine B (Fox) and deionized water (W) were included as controls. (**b**) Complementation analysis of the *B. cenocepacia feuA fhuA* double TBDT mutant using the disc diffusion assay. Filter discs were spotted with ferrichrome or ferrioxamine B (control) and applied to assay plates seeded with the H111ΔpobAΔfeuAΔfhuA mutant containing the pSRKKm-feuA or pSRKKm-fhuA complementation plasmids or pSRKKm, as indicated. (**c**) Effect of inactivation of *feuA* and *fhuA*, separately and together, on ferrichrome-stimulated growth of *B. cenocepacia* in iron-limited broth culture. Cultures were grown in M9-CAA medium containing DTPA and 10 µM ferrichrome at 37 °C. (**i**) Growth of the Δ*feuA*, Δ*fhuA* and Δ*feuA*Δ*fhuA* TBDT mutants compared to the H111ΔpobA parent strain. (ii) Growth curves of the Δ*feuA*Δ*fhuA* mutant containing individual pSRKKm-feuA or pSRKKm-fhuA complementation plasmids or pSRKKm. The plasmid-less Δ*feuA*Δ*fhuA* mutant and the Δ*pobA* parent strain were included for comparison. Data points represent the means of three independent experiments (*n*=3). Error bars, ±sd. ****P<0.0001, ***P<0.001, **P<0.01, *P<0.05, ns=not significant.

While *B. cenocepacia* employs FhuA for uptake of the hydroxamate siderophores ferrioxamine B, TAF, rhodotorulic acid and alcaligin, the utilization of cepabactin and ferrichrome must require a different TBDT in addition to or instead of FhuA. In the phylogenetic tree showing the evolutionary relationships between the *B. cenocepacia* TBDTs, FhuA was observed to be most closely related to the TBDT encoded by the BCAL2281 gene locus, here referred to as FeuA (Fig. 2), and therefore this protein was deemed as a possible additional hydroxamate TBDT. To investigate this possibility, the *feuA* gene was inactivated in the Δ*pobA* strain, and the resultant mutant, H111ΔpobAΔfeuA, was screened for utilization of ferrichrome and the structurally related siderophore ferricrocin by the disc diffusion assay. However, this mutant was able to utilize both of these siderophores, indicating that FeuA does not serve as the sole outer membrane transporter for ferrichrome-type siderophores (Fig. 4a). As it has been demonstrated that ferrichrome can be transported across the outer membrane of *P. aeruginosa* PAO1 by two TBDTs, FiuA (PA0470) and FpvB (PA4168) [22–24, 28], we hypothesized that FhuA and FeuA may exhibit overlapping specificity for ferrichrome-type siderophores. Therefore, a Δ*feuA*Δ*fhuA* double TBDT knock-out mutant was generated and tested for its ability to utilize this class of siderophore. The disc diffusion assay revealed that inactivation of both of these TBDTs in combination inhibited ferrichrome- and ferricrocin-promoted growth of the *B. cenocepacia* Δ*pobA* strain under low iron conditions (Fig. 4a). Introduction of a plasmid expressing either *fhuA* or *feuA* into the double TBDT mutant restored the ability of the bacterium to take advantage of ferrichrome under conditions of iron limitation, confirming that both TBDTs can mediate uptake of iron-loaded ferrichrome (Fig. 4b).

As both FhuA and FeuA can serve to import iron-loaded ferrichrome, the relative contribution of each TBDT was evaluated by comparing ferrichrome-promoted growth of the individual TBDT mutants in iron-limited liquid medium. As expected, ferrichrome supported growth of the *fhuA* and *feuA* mutants under these conditions, and the growth rates of the two mutants were similar (Fig. 4c i). However, the ferrichrome-promoted growth rate of the individual TBDT mutants was not as high as that of the H111ΔpobA parent strain, suggesting that under these conditions both TBDTs are required for optimum utilization of iron-loaded ferrichrome. In contrast, growth of the double TBDT mutant in liquid medium was not stimulated by ferrichrome. Introduction of a plasmid expressing *fhuA* or *feuA* into the double TBDT mutant resulted in full complementation of the growth rate defect (Fig. 4c ii).

The *feuA fhuA* double TBDT mutant was observed to form a zone of growth around a filter disc impregnated with cepabactin, indicating that uptake of this siderophore across the outer membrane involves an alternative TBDT (Fig. S6a). This conclusion was supported by the observation that growth of the double TBDT mutant in iron-limited liquid medium was stimulated by cepabactin to a similar degree as that of the Δ*pobA* parent strain (Fig. S6b). The TBDT responsible for cepabactin transport was not investigated further in this study. As FeuA seems to be specific for ferrichromes, it was named accordingly (ferrichrome utilization protein A).

### Identification of a PepSY_TM domain-containing protein required for hydroxamate siderophore utilization

**Fig. 5.**
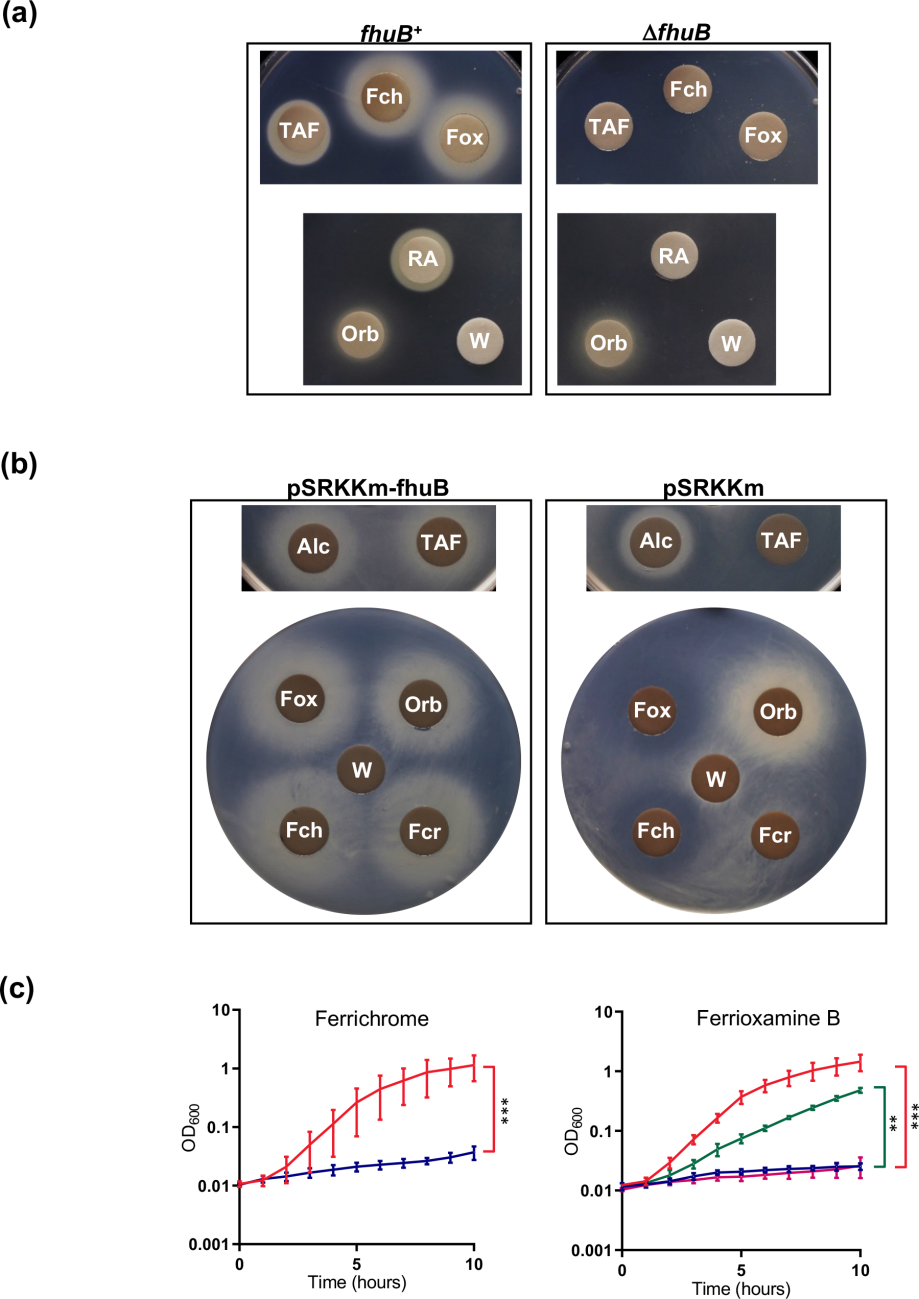
Effect of inactivation of the *fhuB* gene on hydroxamate siderophore utilization by *B. cenocepacia*. (**a**) Analysis of ferrichrome (Fch), ferrioxamine B (Fox), triacetylfusarinine C (TAF) and rhodotorulic acid (RA) utilization by the Δ*fhuB* mutant compared to the H111∆pobA parent strain (*fhuB^+^*) using the disc diffusion assay. Ornibactin (Orb) and deionized water (W) were used as positive and negative controls, respectively. (**b**) Complementation analysis of the Δ*fhuB* mutant using the disc diffusion assay. Filter discs spotted with triacetylfusarinine C, alcaligin (Alc), ferrioxamine B, ferrichrome or ferricrocin (Fcr) were applied to assay plates seeded with the H111ΔpobAΔfhuB mutant containing plasmid pSRKKm-fhuB or the empty vector, pSRKKm, as indicated. Ornibactin and deionized water were used as positive and negative controls, respectively. (**c**) Growth of the H111ΔpobAΔfhuB mutant (blue growth curve) in iron-depleted M9-CAA medium supplemented with DTPA and either ferrichrome or ferrioxamine B (10 µM) compared to the *fhuB^+^* parent strain (red growth curve). Also shown in the right-hand graph is the growth of the H111ΔpobAΔfhuB mutant containing plasmid pSRKKm-fhuB (green growth curve) or the empty vector, pSRKKm (magenta growth curve), in medium containing ferrioxamine B. Data points represent the means of three independent experiments (*n*=3). Error bars, ±sd. ***P<0.001, **P<0.01.

It was previously proposed that the product of the *P. aeruginosa* PA2465 gene (FoxB), a PepSY_TM domain-containing protein, plays a role in utilization of some ferrioxamine-type siderophores ([46, 47] see Discussion). To determine whether the *B. cenocepacia* FoxB homologue, FhuB (BCAL0117), is involved in utilization of ferrioxamine B, an *fhuB* null allele was introduced into the H111 Δ*pobA* mutant and the resultant mutant strain was screened for utilization of this siderophore. The disc diffusion assay showed that ferrioxamine B was unable to promote growth of the *fhuB* deletion mutant under iron starvation conditions (Fig. 5a). Moreover, the mutant was also unable to utilize the hydroxamate siderophores ferrichrome, ferricrocin, TAF and rhodotorulic acid (Fig. 5a, b). In contrast, utilization of alcaligin was not adversely affected by deletion of *fhuB* and nor was utilization of the hydroxamate-hydroxycarboxylate siderophore, ornibactin (Fig. 5a, b). The effect of *fhuB* inactivation on utilization of cepabactin was not investigated. The disc diffusion bioassay was also used to demonstrate that the ability of ferrioxamine B, ferrichrome, ferricrocin and TAF to promote growth of the mutant was restored upon introduction of a plasmid expressing the wild-type *fhuB* gene (Fig. 5b).

The participation of *fhuB* in hydroxamate siderophore utilization was confirmed by monitoring growth of the Δ*pobA*Δ*fhuB* mutant under iron-restricted conditions in liquid medium in the presence or absence of ferrioxamine B or ferrichrome as representatives of utilizable hydroxamate xenosiderophores. Growth of the mutant in iron-limited medium was demonstrated to be severely limited regardless of the presence of ferrioxamine B or ferrichrome in the medium (Fig. 5c). A genetic complementation experiment was also performed which showed that introduction of plasmid-borne copies of the *fhuB* gene into the mutant restored its ability to utilize ferrioxamine B (Fig. 5c). These results demonstrate that FhuB is required for utilization of the same subset of hydroxamate siderophores that are transported by the FhuA TBDT, with the exception of alcaligin.

### *B. cenocepacia* is resistant to the ferrichrome-like sideromycin albomycin δ_2_

Given that the molecular structure of the naturally occurring sideromycin, albomycin δ_2_, resembles ferrichrome and it is transported through the ferric hydroxamate transport system in some bacteria [49, 50], we tested the sensitivity of *B. cenocepacia* to this sideromycin. However, we observed that albomycin δ_2_ did not inhibit the growth of *B. cenocepacia* (Fig. S7).

### Components of the *B. cenocepacia* ferric-hydroxamate siderophore utilization system are conserved in related bacterial species

Our *in silico* analysis revealed that FhuA and FeuA orthologues are encoded in the genomes of other *B. cenocepacia* strains, including K56-2 and ST32 (Table S5). On the other hand, members of the closely related species, *Burkholderia orbicola*, may either contain both TBDTs (as in strains AU1054, PC184 and HI2424) or only one of them (strain MC0-3 encodes only FhuA). We have also observed the presence of an FhuA orthologue in more than ten other more distantly related Bcc members such as *B. cepacia, B. contaminans, B. dolosa, B. lata, B. stabilis, B. stagnalis, B. pseudomultivorans* and *B. ubonensis* (Table S5). Fewer Bcc species possess the FeuA orthologue, including *B. cepacia, B. lata*, *B. multivorans* and *B. stabilis* (Table S5). Thus, along with many *B. cenocepacia* strains, individual strains of *B. anthina*, *B. arboris*, *B. orbicola*, *B. cepacia*, *B. lata*, *B. paludis* and *B. stabilis* possess both of the hydroxamate TBDTs. Outside of the Bcc, four members of the plant-pathogenic *Burkholderia glumae* group (*B. glumae*, *B. gladioli*, *B. perseverans* and *B. plantarii*) also encode FeuA but not FhuA, whereas members of the *Burkholderia pseudomallei* complex lack both of these TBDTs. TBDTs that are highly similar to FhuA and FeuA were also observed in members of related genera such as *Paraburkholderia* and *Caballeronia*, i.e. *P. fungorum* and *C. novacaledonica*.

Putative orthologues of the *B. cenocepacia* FhuB protein were found to be present in other members of the Bcc, such as *B. orbicola*, *B. cepacia*, *B. lata*, *B. stabilis*, *B. multivorans* and *B. ubonensis*. On the other hand, Bcc species that lack both FhuA and FeuA, such as *B. ambifaria* and *B. vietnamiensis*, generally also lack FhuB (Table S5). This is also true of the *B. pseudomallei* complex. Curiously, orthologues of FhuB are also absent from the four members of the plant-pathogenic *B. glumae* group, suggesting that an alternative route for delivery of ferrichrome-bound iron from the periplasm to the cytoplasm may exist in these species. We also observed the presence of putative FhuB orthologues in some members of *Caballeronia* and *Paraburkholderia*, i.e. *C. mineralivorans* and *P. gardini*.

## Discussion

The experiments described here demonstrate the ability of *B. cenocepacia* to exploit a broad range of hydroxamate xenosiderophores to sustain its growth under iron-limiting conditions. These include siderophores belonging to different structural classes and with a different number of iron-chelating groups, including macrocycles containing three *exo*-hydroxamate groups (i.e. ferrichrome, ferricrocin), macrocycles containing two or three *endo*-hydroxamate groups (i.e. alcaligin and TAF, respectively), diketopiperazines containing two *exo*-hydroxamate groups (rhodotorulic acid), linear trihydroxamates (i.e. ferrioxamine B) and 1-hydroxypyridin-2-one monohydroxamates (i.e. cepabactin) (Fig. S1). Perhaps surprisingly, coprogen, which is structurally related to rhodotorulic acid, was not employed as a xenosiderophore by *B. cenocepacia*. The ability of *B. cenocepacia* to utilize these siderophores is likely to reflect the different niches that it can adapt to in the environment, particularly as this species can exist in soil as a saprotroph where it is likely to encounter *Streptomyces* species that produce ferrioxamine B (such as *S. ambofaciens*, *S. coelicolor*, *S. pilosus* and *S. viridosporus*) and various fungal species that produce ferrichromes, TAF and rhodotorulic acid (including *Aspergillus*, *Fusarium*, *Neurospora*, *Penicillium*, *Rhodotorula* and *Ustilago* spp.) [51–56]. Given the variety of hydroxamate siderophores that can be utilized by *B. cenocepacia*, it is very likely that it can also take advantage of other ferrioxamines (e.g. ferrioxamines E and G) and structurally related iron-chelating compounds such as coelichelin that are also produced by *Streptomyces* spp., as well as other fungally derived ferrichromes (i.e. ferrichrysin, ferrirubin and ferrirhodin) and molecules related to alcaligin (putrebactin, avaroferrin and bisucaberin produced by *Shewanella putrefaciens*) that were not tested in this study, thereby extending the range of environmental species *B. cenocepacia* may take advantage of [53, 57].

As *B. cenocepacia* and other Bcc members have been increasingly observed in polymicrobial respiratory tract infections in CF patients [58, 59], the ability of these organisms to use hydroxamate siderophores to pirate iron may provide *B. cencocepacia* with a similar advantage during infection, depending on the presence of co-infecting hydroxamate siderophore producers such as *Achromobacter xylosoxidans* (alcaligin), *Aspergillus fumigatus* and *Aspergillus nidulans* (TAF, ferricrocin, ferrichrome) and more rarely *Bordetella bronchiseptica* (alcaligin), *Fusarium* (TAF, ferricrocin), *Penicillium* (TAF, ferrichrome) and *Rhodotorula* (rhodotorulic acid) spp. [56, 60–66]. *B. cenocepacia* may also be able to use iron-loaded cepabactin where the CF patient has been co-infected with a cepabactin-producing member of the Bcc, such as *B. cepacia* [67].

The genome of *B. cenocepacia* strain H111 encodes 24 putative TBDTs, the highest number of TBDTs possessed by a Bcc member (Fig. 2; S.M.H. and M.S.T., unpublished data). This suggests that *B. cenocepacia* can utilize a broader spectrum of exogenous siderophores than related species, which in turn may provide an advantage over other bacterial species seeking to exploit the same niche. We demonstrated that at least two of these TBDTs, FhuA and FeuA, play a role in utilization of xenosiderophores exclusively harbouring hydroxamate iron-chelating ligands. The most probable function of these proteins, based on the role of other TBDT family proteins, is the active transport of iron–hydroxamate siderophore complexes across the outer membrane. FhuA appears to be solely responsible for transport of the hydroxamate siderophores ferrioxamine B, TAF, rhodotorulic acid and alcaligin. In addition, FhuA is able to function as a ferrichrome transporter. The versatility of this transporter contrasts with the more restricted range of substrates of the *P. aeruginosa* FoxA and FiuA TBDTs that were used as search queries, and may to some extent explain their low degree of similarity to FhuA.

Among the other *B. cenocepacia* TBDTs, FeuA is most similar to FhuA, and consistent with this, FeuA was also shown to facilitate ferrichrome-mediated iron acquisition. Redundancy among TBDTs is not unique to *B. cenocepacia*. For example, while it has been established for some time that *P. aeruginosa* PAO1 employs the FoxA (PA2466) and FiuA (PA0470) TBDTs in the uptake of iron-loaded ferrioxamine B and ferrichrome, respectively [23, 24], a recent study has shown that the FpvB (PA4168) TBDT can transport both of these iron-siderophore complexes [28, 68]. Moreover, *P. aeruginosa* PAO1 encodes two TBDTs, [PfeA (PA2688) and PirA (PA0931)], that can transport iron-loaded enterobactin, and three TBDTs [HxuA (PA1302), HasR (PA3408) and PhuR (PA4710)] play a role in haem uptake in this pathogen [69–71]. The possession of two or more TBDTs that can transport the same siderophore is probably a consequence of their differing substrate specificities that in combination would allow for a broader range of siderophores to be utilized from any particular siderophore class. Although both FhuA and FeuA were demonstrated to transport a range of bis- and tris-hydroxamate siderophores, they were not required for utilization of the heterocyclic mono-hydroxamate siderophore, cepabactin. Therefore, the transport of cepabactin is likely to involve an alternative TBDT instead of, or in addition to, FhuA and/or FeuA.

Our investigation revealed that FhuB is required for utilization of various hydroxamate siderophores, including ferrioxamine B, ferrichrome, TAF and rhodotorulic acid, although not alcaligin. FhuB exhibits a high degree of similarity to the proposed ferrioxamine and ferrichrome permease of *P. aeruginosa*, FoxB (PA2465; 36 % identity, 58 % similarity) [46]. However, the role of FoxB as a cytoplasmic membrane transporter of these siderophores is open to question. For example, inactivation of the PA0476 gene (*fiuB*), encoding a predicted cytoplasmic membrane permease, abolished ferrichrome-mediated iron uptake by *P. aeruginosa* PAO1, suggesting that FiuB is the sole means of translocation of the iron–ferrichrome complex across the cytoplasmic membrane [24]. Moreover, *B. cenocepacia* FhuB and *P. aeruginosa* FoxB, and related proteins, are commonly annotated in microbial genome databases as ‘PepSY domain-containing proteins’. The PepSY domain is a loosely conserved domain of 60–90 aa that contains a conserved hDhXXG motif (where ‘h’ is a hydrophobic residue) located near the C-terminus [72]. FhuB and related proteins form a subclass of such proteins that possess two tandem PepSY domains, each of which is flanked on either side by a conserved transmembrane domain (TMD) known as PepSY_TM (Fig. S8). These TMDs have common features, such as a highly conserved histidine located N-terminal to each TMD and an (S/T)G motif located near the C-terminus of each TMD, and are predicted to be orientated in the cytoplasmic membrane such that each of the two PepSY domains is presented to the periplasmic compartment ([72] Fig. S9). The region containing the tandem PepSY domains together with the flanking PepSY_TM domains is annotated in the NCBI database as ‘PepSY-associated TM region’ or ‘PiuB’ (COG3182), with the latter term being derived from the name of the *P. aeruginosa* PA4513 gene product which also possesses a PepSY-associated TM region ([73] Fig. S9). In some proteins of this subclass, such as *B. cenocepacia* FhuB and the *P. aeruginosa* PiuB-type proteins PA1909, PA2403 (FpvG) and PA2465 (FoxB), the conserved PiuB/PepSY-associated TM region accounts for almost the entire length of the polypeptide, and therefore these proteins harbour only four TMDs. This contrasts with the *P. aeruginosa* ferrichrome and pyochelin single subunit transporters, FiuB and FptX, both of which are predicted to contain 12 TMDs per polypeptide (based on analysis by DeepTMHMM v1.0.24 ([74] Fig. S9).

Although there is little evidence to support a role for FhuB and other PiuB-type proteins as cytoplasmic membrane transporters for ferric–siderophore complexes, the genomic context of most genes encoding PiuB-type proteins suggests a role in siderophore-mediated iron assimilation. For example, in *P. aeruginosa* PAO1 the genes encoding the PiuB-type PepSY domain-containing proteins PA1909, PA2465 (FoxB), PA3789 and PA4513 (PiuB) are located adjacent to genes encoding the ferric-siderophore/copper chelate TBDTs FemA, FoxA, OprC and PiuA, respectively, while PA2403 (FpvG) and PA4219 (FptC) are located within the *fpvGHJKCDEF* and *fptHIABCX* gene clusters that encode systems required for translocation of pyoverdine- and pyochelin-acquired iron across the cytoplasmic membrane [17, 18, 75, 76]. Interestingly, both pyoverdine- and pyochelin-mediated iron uptake are proposed to involve periplasmic release of iron from the ferric-siderophore complex and its subsequent translocation across the cytoplasmic membrane as a free ion, raising the possibility that PiuB-type PepSY domain-containing proteins participate in this process [76, 77]. Accordingly, an investigation into the role of PA2403 (FpvG) in pyoverdine-mediated iron uptake revealed that this protein does indeed participate in iron dissociation from the ferric-siderophore complex in the periplasm, with FpvG probably functioning as a ferric iron reductase that promotes periplasmic release of the iron atom from the siderophore by converting it to the ferrous form [75].

**Fig. 6.**
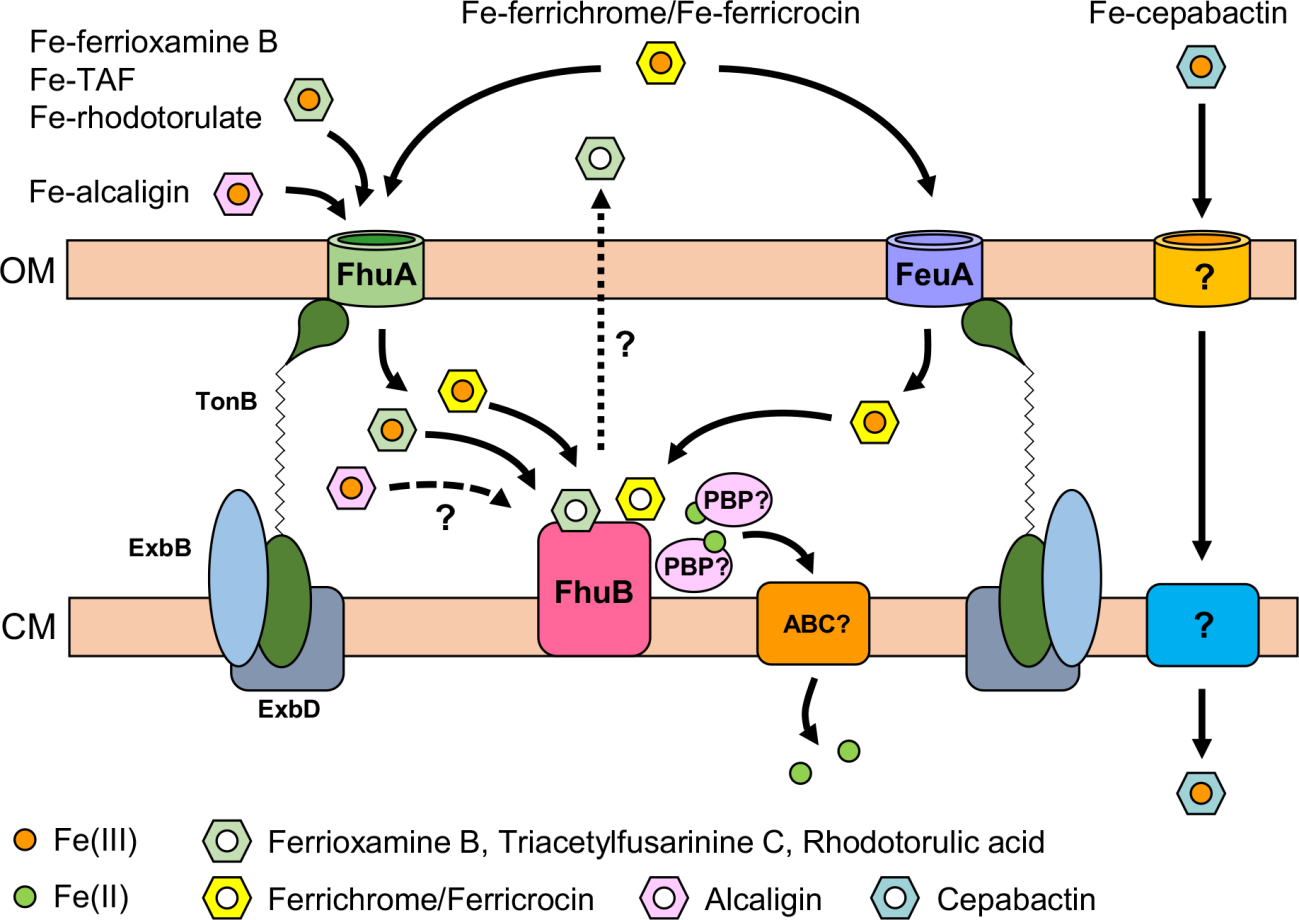
Model for hydroxamate siderophore utilization in *B. cenocepacia*. Most hydroxamate siderophores shown are translocated across the outer membrane (OM) by the FhuA TBDT. This includes ferrichrome-type siderophores which are also recognized by the FeuA TBDT. The TonB system, consisting of TonB, ExbB and ExbD, is required to energize TBDTs. Once internalized, iron is proposed to be reductively released from many of the hydroxamate siderophores by the PiuB-like FhuB protein (alcaligin utilization is not dependent on FhuB but it is possible that ferric-alcaligin may also be processed by this system). Based on other systems that employ periplasmic PiuB-type reductases, reductively released ferrous iron is likely to be conveyed to an unknown ABC transporter (ABC) by a periplasmic binding protein (PBP) for delivery into the cytoplasm. Cepabactin utilization involves an unidentified TBDT and an unknown cytoplasmic membrane (CM) transport system, and the involvement of FhuB has not been determined. For clarity, the figure does not show the potential involvement of PBPs in chaperoning ferric-siderophore complexes to the FhuB iron-siderophore reductase.

A subsequent study on the *P. aeruginosa* FoxB protein confirmed that members of this group of proteins do indeed possess periplasmic ferric iron reductase activity [47]. The authors proposed that PiuB-type proteins shuttle electrons from the cytoplasm to the ferric-siderophore complex bound within a cavity created by the two periplasmically located PepSY domains where reduction of the ferric ion would promote ferrous iron release [47]. Therefore, we conclude that *B. cenocepacia* FhuB is likely to function in an analogous fashion to *P. aeruginosa* FoxB, and results in reductive dissociation of iron from the periplasmically located iron-loaded hydroxamate xenosiderophore. It is possible that this activity may also extend to ferric-alcaligin, but there may be redundancy of reductases for this siderophore in *B. cenocepacia* or a system for translocating ferric-alcaligin across the cytoplasmic membrane may also exist. A model for the utilization of hydroxamate xenosiderophores by *B. cenocepacia*, showing how FhuB may participate in this process, is shown in Fig. 6.

Iron released from pyoverdine through the action of the FpvG reductase is translocated across the cytoplasmic membrane of *P. aeruginosa* by the FpvDE ABC transporter [78, 79]. Similarly, we suggest that the PiuB-type protein, FptC (PA4219), may be responsible for reductive release of iron from pyochelin, with the free iron atom then available for translocation across the cytoplasmic membrane by the PchHI ABC transporter [76, 80]. In contrast to *fpvG* and *fptC*, the *fhuB* and *foxB* genes of *B. cenocepacia* and *P. aeruginosa*, respectively, are not located in gene clusters that encode ABC transporters, so the transport systems required for uptake of the ferrous iron atom released from hydroxamate xenosiderophores in these species remain to be identified.

Following reductive release of iron from ferric-pyoverdine, the deferrated siderophore is recycled by an efflux mechanism that requires the periplasmic binding protein, FpvF, and the PvdRT-OpmQ ABC exporter [77–79]. Thus, periplasmic release of iron from hydroxamate xenosiderophores in *Burkholderia* raises the possibility that these siderophores are also subsequently exported. This may obviate the requirement for inactivation of the desferri-siderophore as occurs for cytoplasmic ferrichrome in *E. coli* and *P. aeruginosa* (presumably as a means of reducing potential toxic effects of the chelator), particularly if the released ferrous iron is escorted to a cytoplasmic membrane transporter by a chaperone protein, as the iron atom is at little risk of being recaptured by the aposiderophore. During pyoverdine utilization by *P. aeruginosa*, this task is fulfilled by the periplasmic binding protein, FpvC, which in turn is proposed to dock with the FpvDE ABC transporter to facilitate transfer of the iron atom to the cytoplasm [75, 78]. However, the existence of an efflux system for deferrated hydroxamate siderophores and a periplasmic binding protein system for chaperoning ferrous ions released from such siderophores has yet to be investigated in *B. cenocepacia*.

Our investigation showed that the antimicrobial compound albomycin, a naturally occurring sideromycin that is structurally related to ferrichrome, exhibited no detectable bactericidal activity against *B. cenocepacia*. This is consistent with the previous observation that *B. cepacia* is resistant to albomycin [81]. The dependence of *B. cenocepacia* on the FhuB periplasmic reductase system for the utilization of some hydroxamate siderophores strongly suggests that there is no alternative cytoplasmic membrane transport system that would deliver these siderophores to the cytoplasm. Therefore, the resistance of *Burkholderia* species to albomycin is probably also due to the absence of a mechanism for vectoring albomycin to its cytoplasmic target [49, 82, 83].

Periplasmic deferration and recycling of endogenously produced siderophores is economical, as it spares the energy that would otherwise be involved in the resynthesis of such siderophores. However, adoption of an analogous mechanism for xenosiderophores would not offer such an advantage. Nevertheless, the observation that members of the genus *Burkholderia* are resistant to albomycin provides a rationale for periplasmic removal of iron from hydroxamate xenosiderophores. For bacteria living in an environment where they encounter cytoplasmically active sideromycins that are structurally analogous to classes of siderophore they are capable of utilising, it may be advantageous to employ a system where these siderophores (and by extension the related sideromycins) do not access the cytoplasm. Thus, *Burkholderia* species, many of which occupy niches such as soil, where they will encounter members of the genus *Streptomyces* that produce albomycin [84, 85], would potentially greatly benefit from a system that denies access of the sideromycin to the cytoplasm. Another soil bacterium, *Streptomyces violaceus*, produces the toxic ferrioxamine B analogue, salmycin A, which also has a cytoplasmic target [86, 87]. Although we have not tested the toxicity of this compound towards *Burkholderia* spp., survival in an environment where such sideromycins also occur would provide a selective pressure for *Burkholderia* and other successful soil dwellers to adopt a system that also avoids cytoplasmic uptake of ferrioxamines. By employing a single system that processes a range of hydroxamate siderophores in the periplasm, including both ferrichrome and ferrioxamine B, *B. cenocepacia* has evolved an economical way of keeping itself in the bacterial arms race. In contrast, the observation that *E. coli* employs a mechanism for utilizing these siderophores in which the ferric-siderophore complex enters the cytoplasm may suggest that, in its normal niche, this bacterium would seldom encounter producers of ferrichrome- and ferrioxamine-based sideromycins.

## Supplementary Data

Supplementary material 1
