# Supplementary material for: Identification of a system for hydroxamate xenosiderophore-mediated iron transport in Burkholderia cenocepacia
Source: Microbiology (Reading). 2024 Jan 8;170(1):001425. doi: 10.1099/mic.0.001425 (PMC10866019; doi:10.1099/mic.0.001425)
Supplement: Supplementary material 1 [file mic-170-1425-s001.pdf]

SUPPLEMENTARY INFORMATION

Table S1. Bacterial strains.

Table S2. Plasmids.

Table S3. Oligonucleotides.

Table S4. List of putative TBDTs present in *B. cenocepacia* J2315 and H111.

Table S5. Potential orthologues of the *B. cenocepacia* FeuA, FhuA and FhuB proteins in related bacteria.

Fig. S1. Hydroxamate siderophore structures.

Fig. S2. Characterisation of a *B. cenocepacia pobA* deletion mutant.

Fig. S3. Alignment *of B. cenocepacia* TBDT amino acid sequences.

Fig. S4. Effect of inactivation of *orbA* on hydroxamate siderophore utilisation.

Fig. S5. Genomic context of *B. cenocepacia* TBDT genes.

Fig. S6. Effect of cepabactin on growth of a *B. cenocepacia* Δ*feuA* Δ*fhuA* mutant in iron-limited medium.

Fig. S7. Effect of albomycin on growth of *B. cenocepacia.*

Fig. S8. Amino acid sequence alignment of representative PepSY_TM domain-containing proteins (PiuB-type proteins).

Fig. S9. TMD analysis of the FptX, FiuB, FoxB and FpvG proteins of *P. aeruginosa* PAO1 and the *B. cenocepacia* H111 FhuB protein.

Table S1. Bacterial strains

Bacterial strain	Genotype/description[a,b]	Source/reference
Burkholderia cenocepacia		
715j	Clinical isolate, *tssM*::ISUmu23 (Orb$^+$ Pch$^+$)	(1-3)
AHA27	715j pobA::mini-Tn*5*CmlacZYA (Orb$^-$ Pch$^-$ Cm^R)	(4)
AHA27ΔfhuA	AHA27 containing *fhuA*::TpTer allele (Orb$^-$ Pch$^-$ Cm^R Tp^R)	This study
H111	Clinical isolate (Orb$^+$ Pch$^+$)	(5)
H111ΔpobA	H111 containing in-frame deletion within the *pobA* gene (Orb$^-$ Pch$^-$)	This study
H111ΔpobAΔfhuA	H111ΔpobA containing *fhuA*::TpTer allele (Orb$^-$ Pch$^-$ Tp^R)	This study
H111ΔpobAΔfhuB	H111ΔpobA containing in-frame deletion within the *fhuB* gene	This study
H111ΔpobAΔfeuA	H111ΔpobA containing in-frame deletion within the *feuA* gene (Orb$^-$ Pch$^-$)	This study
H111ΔpobAΔorbA	H111ΔpobA containing *orbA*::TpTer allele (Orb$^-$ Pch$^-$ Tp^R)	This study
H111ΔpobAΔfeuAΔfhuA	H111ΔpobAΔfeuA containing *fhuA*::TpTer allele (Orb$^-$ Pch$^-$ Tp^R)	This study
Pseudomonas aeruginosa		
PAO1 pch$^-$ pvd$^-$	PAO1 containing deletion of entire *pch* gene cluster (PvdI$^-$ Pch$^-$)	(6)
Escherichia coli		
JM83	F$^-$ *ara* Δ(*gpt-mhpC*) *rfbD1 thiE rpsL* (Sm^R) *ϕ80dlacZ*Δ*M15*	(7)
SM10(λpir)	*thi-1 thr-1$_{(Am)}$ leuB6 fhuA21 lacY1 [Δ(lacY-ykgF) INV(ykgE-phoE) Δcrl] glnV44 recA glvB::*RP4-2-Tc::Mu λpir (Km^R)	(8, 9)
CC118(λpir)	*araD139 Δ(ara-leu)7697 ΔlacX74 galE15 galK16 phoA20 thi-1 rpsE rpoB* (Rf^R) *argE$_{(Am)}$ recA*1 λpir	(10)
S17-1(λpir)	*pro-82* [Δ(*frsA-ykfC*)] Δ(*ynaJ-fnr*) *thiE endA1 recA creC510 rfbD1 glnV44 ttdB hsdR17$_{(Op)}$ rbsR guaC$_{(Op)}$ abgB$_{(Oc)}$ rpoS396*$_{(Am)}$ *rssB*$_{(Am)}$ *sgrR$_{(Oc)}$ narG$_{(Am)}$ ftsP$_{(Am)}$ cadB$_{(Op)}$ cpdB$_{(Am)}$::IS1 crl*::IS*1 kdgR*::IS*5 gatB*::IS*5 fimE*::IS*1 lrhA*::IS*3 srlD-gutM::IS4 mhpC*::RP4-2-Tc::Mu1-*kan*::Tn*7 pstS-glmS*::Tn*7^c* λpir (Tp^R Sm^R)	(8, 9, 11)
GM48	F$^-$ *thr-1 leuB6 thiE1 lacY1 [Δ(lacY-ykgF) INV(ykgE-phoE) Δcrl] galK2$_{(Oc)}$ galT22 araC14 fhuA tsx-78 dam-3 dcm-6 glnV44*	(12)
MC1061	*araD139 Δ(ara-leu)7697 ΔlacX74* [Δ(*codB-lacI*)3] *galK16 galE15 mcrA0 relA1 rpsL150 spoT1 mcrB1 hsdR2 λ$^-$ e14$^-$* (Sm^R)	(13)

[a]Phenotype abbreviations: Rf^R, rifampicin resistant; Sm^R, streptomycin resistant; Tp^R, trimethoprim resistant; Km^R, kanamycin resistant; Orb, ornibactin phenotype; Pch, pyochelin phenotype; TpTer, cassette containing *dfrB2* gene (specifying resistance to trimethoprim) fused to *E. coli rrnB* T1T2 terminators (14).
[b]The *B. cenocepacia orbA*, *pobA*, *fhuA*, *fhuB* and *feuA* genes correspond to gene loci BCAL1700, BCAL2248, BCAL0116, BCAL0117 and BCAL2281 in the *B. cenocepacia* type strain, J2315, and I35_RS08065, I35_RS10890, I35_RS00620, I35_RS00625 and I35_RS11045 in strain H111, respectively.
[c]A second copy of Tn*7* is inserted in the *pstS-glmS* intergenic region in S17-1(λpir).

Table S2. Plasmids

Plasmid name	Description	Source/reference
pEX18Tp-pheS	Gene replacement vector derived from ColE1 with mutated phenylalanyl tRNA synthase α-subunit gene (*pheS**) for cPhe counter-selection (Tp^R)	(15)
pSNUFF	Gene replacement vector derived from pEX18Tp-pheS containing TpTer cassette (Tp^R)	(14, 15)
pSNUFF3Cm	pSNUFF derivative containing *catA2* gene and *I-SceI* recognition site(Tp^R Cm^R)	(Spiewak and Thomas, unpublished results)
pSHAFT-GFP	R6K-derived allelic replacement vector (Ap^R)	(14)
pSHAFT2	Mobilisable suicide vector derived from pUT, oriR6K (Ap^R Cm^R)	(14)
pBBR1MCS	Mobilisable BHR cloning vector IncP- and ColE1-compatible, *lacZ*α MCS (Cm^R)	(16, 17)
pSRKKm	pBBR1MCS-2 derivative containing *lacI* and an additional *lac* operator for tight regulation of P_{lac} (Km^R)	(18)
pDAI-SceI-pheS	Self-curable plasmid expressing I-SceI endonuclease (Tc^R)	(19)
p34E-TpTer	p34E derivative containing TpTer cassette (Ap^R Tp^R)	(14)
pSNUFF-pobA	pSNUFF containing the *B. cenocepacia pobA* gene	This study
pSNUFFΔpobA	pSNUFF-pobA containing an in-frame deletion within *pobA*	This study
pSHAFT-GFP-fhuA	pSHAFT-GFP containing the *B. cenocepacia fhuA* gene	This study
pSHAFT-GFP-ΔfhuA	pSHAFT-GFP containing insertionally inactivated *fhuA* gene (*fhuA*::TpTer)	This study
pSNUFF-feuA	pSNUFF containing the *B. cenocepacia feuA* gene	This study
pSNUFF-ΔfeuA	pSNUFF-feuA containing an in-frame deletion within the *feuA* gene	This study
pSNUFF3Cm-fhuB	pSNUFF3 containing the *B. cenocepacia fhuB* gene	This study
pSNUFF3Cm-ΔfhuB	pSNUFF3-fhuB containing in-frame deletion within the *fhuB* gene	This study
pBBR1-orbA	pBBR1MCS carrying a segment of the *B. cenocepacia* BCAL1700 gene	This study
pBBR1-ΔorbA	pBBR1-BCAL1700 with TpTer cassette inserted in the *Bam*HI site of *orbA*	This study
pSHAFT2-ΔorbA	pSHAFT containing insertionally inactivated *orbA* gene (*orbA*::TpTer)	This study
pSRKKm-fhuA	pSRKKm carrying the entire *fhuA* gene	This study
pSRKKm-feuA	pSRKKm carrying the entire *feuA* gene	This study
pSRKKm-fhuB	pSRKKm carrying the entire *fhuB* gene	This study

Abbreviations: Ap^R, encodes ampicillin resistance; Cm^R, encodes chloramphenicol resistance; Km^R, encodes kanamycin resistance; Tp^R, encodes trimethoprim resistance; Tc^R, encodes tetracycline resistance; TpTer, *dfrB2* gene fused to *rrnB* T1T2 terminators (14); cPhe, DL-4-chlorophenylalanine.

Table S3. Oligonucleotides

Oligonucleotide name	Sequence (5' → 3')[a,b]	Restriction sites present
M13for	gtaaaacgacggccagt	-
M13BACTHrev	gtgtggaattgtgagcggat	-
M13rev	caggaaacagctatgac	-
pEX18Tpfor	gaagccagttaccttcgga	-
pEX18Tprev	ttgtcggtgaacgctctcct	-
pUTcatrev	aacggttgtggacaacaagc	-
catendout	gatgtgtgataacatactga	-
pobAfor2	gcgcggatcctatgcgtactggcgcgatta	*Bam*HI
pobArev2	gcgcaagcttttctcgaccaccttcttcgt	*Hin*dIII
pobAfor4	gcgccatcgaacgtgcatgt	-
pobArev3	gaatgcggcgtgacgaccaa	-
BCAL0116forfull	gcgcggatccg**TAA**ccttgtacgcggcatggaat	*Bam*HI
BCAL0116revfull	gcgcaagcttgggaaaatcccgaggccatt	*Hin*dIII
BCAL0116for	gcgctctagatacaagctcggcgacgtgtt	*Xba*I
BCAL0116rev	cgcgtagacatcattcacgc	-
BCAL0116forout	gttctcgacgaccatcgtca	-
BCAL0116revout	gttcgctgcgttcgcgtatt	-
BCAL0117for	gcgcggatccg**TAA**gcgcggtgctcgacatcaaa	*Bam*HI
BCAL0117for2	gcgcggatccgcgagtaaaaatgcgagtaag	*Bam*HI
BCAL0117rev2	gcgcgtcgacacttgccccgcggtccttat	*Sal*I
BCAL0117rev3	gcgcgaattcatcgagaaattgctggagaa	*Eco*RI
BCAL0117forout	tgcgcggccgtgatgttgag	-
BCAL0117revout	gtcgatgccgtcgcacgctt	-
BCAL1700for	gcgcggtaccaagcttagatctatcatcgacgtgcacaccaa	*Bgl*II, *Hin*dIII, *Kpn*I
BCAL1700rev	gcgctctagacatggatgcgtcgtggaaaa	*Xba*I
BCAL1700fullfor	cgctctagag**TAA**gtcgctcgaaaacgggatcgg	*Xba*I
BCAL1700forout	gtcgatccgtacatgatcga	-
BCAL1700revout	cgactggatgtagacgagat	-
BCAL2281for	gcgcaagcttgttccttttgaagagtgcgg	*Hin*dIII
BCAL2281rev	gcgcggtacctccgttcttcacgcaactga	*Acc*65I
BCAL2281forout2	caggatcagcgcaaaggcga	-
BCAL2281revout	ggtgtgcgtgtactacatcg	-

[a]Restriction sites used for cloning are underlined.

[b]Stop codons designed to prevent readthrough translation of vector-encoded *lacZ* mRNA are shown in bold upper-case letters.

Table S4. Identification of putative TBDTs present in *B. cenocepacia* J2315 and H111[a]

J2315 TBDTs	H111 TBDTs	Alternative name	Chromosome location	Number of amino acid residues[b]	Similarity to PA2466 (FoxA)			Similarity to PA0470 (FiuA)		
					Percentage (%)[c]	Coverage[d]	Ranking[e]	Percentage (%)[c]	Coverage[d]	Ranking[e]
BCAL0116	I35_RS00620	FhuA	1	707	42.5	279/656	14	43.1	296/686	14
BCAL1345	I35_RS06170		1	736	44.0	275/626	11	45.2	298/660	9
BCAL1371	I35_RS06295		1	839	45.4	347/765	3	47.0	358/762	3
BCAL1700	I35_RS08065	OrbA	1	755	54.0	360/667	1	54.6	376/689	1
BCAL1709	I35_RS08115		1	712	43.3	284/656	9	42.1	281/667	12
BCAL1777	I35_RS08460		1	906	-	-	-	-	-	-
BCAL1783[f]	I35_RS08490		1	941	-	-	-	-	-	-
BCAL2281	I35_RS11045	FeuA	1	726	44.4	199/458	16	39.5	270/684	16
BCAL3001	I35_RS04375		1	787	52.7	59/112	19	48.2	53/110	20
BCAM0491	I35_RS18505		2	707	43.5	291/669	12	45.2	297/657	11
BCAM0499	I35_RS18545		2	732	46.0	303/660	5	46.2	325/704	5
BCAM0564	I35_RS18860		2	788	45.5	194/426	17	43.6	68/156	18
BCAM0706[f]	I35_RS19580		2	743	42.1	268/636	13	44.9	298/664	10
BCAM0948	I35_RS20820		2	688	47.8	54/113	22	-	-	-
BCAM1187	I35_RS21645		2	739	45.4	296/652	10	43.8	287/657	8
BCAM1571	I35_RS23575		2	694	46.8	80/171	21	53.3	49/092	21
BCAM1593	I35_RS23700		2	642	45.2	166/367	20	39.5	256/648	15
BCAM2007	I35_RS25625		2	747	46.4	310/668	8	42.7	271/635	13
BCAM2224	I35_RS26975	FptA	2	727	43.7	286/654	7	44.4	304/684	6
BCAM2367	I35_RS27690		2	777	46.3	126/272	15	44.6	296/686	19
BCAM2439	I35_RS28095		2	722	44.2	295/667	6	45.8	306/668	7
BCAM2626	I35_RS29035		2	757	46.3	152/328	18	41.9	238/568	17
BCAS0333	I35_RS31745		3	724	53.3	357/669	2	54.5	365/670	2
BCAS0360	I35_RS31880		3	701	43.6	291/668	4	44.9	302/672	4

[a]TBDTs encoded by *B. cenocepacia* strains H111 and J2315 were identified by BLASTP using the mature sequences of PA0470 (FiuA) and PA2466 (FhuA) from *P. aeruginosa* PAO1 as search queries.

[b]The number of amino acids indicated refers to the full-length protein encoded by strain H111.

[c]Percentage similarity over the matching region.

[d]Length of the matching region.

[e]*B. cenocepacia* TBDT with the highest similarity to the search query is ranked as 1.

[f]Pseudogenes in J2315: in-frame translation termination codon in BCAM0706 and frameshift mutation in BCAL1783.

- Not found by search query. BCAL1777 and BCAL1783 were not identified by either search query but were detected by using low ranking *B. cenocepacia* TBDTs as search queries. BCAM0948 was not identified by the PA0470 search query.

Table S5. Potential orthologues of the *B. cenocepacia* FeuA, FhuA and FhuB proteins in related bacteria[a]

Genus	Group	Species	Strain	Locus tag		
				FeuA orthologue	FhuA orthologue	FhuB orthologue
Burkholderia	BCC	*B. ambifaria*	AMMD[T]	-	-	-
		B. anthina	DSM 16086[T]	JQK92_02425	JQK92_29135	JQK92_29130*
			1CH1	J4G50_RS05470	J4G50_RS18190	J4G50_RS18185
		B. anthinoferrum	XXVI[T]	NA	NA	NA
		B. arboris	LMG 24066[T]	BAR24066_06989	BAR24066_04423	BAR24066_04422
			MEC_B345	NLX30_12850	NLX30_00945	NLX30_00950
		B. catarinensis	89[T]	-	-	
		B. cenocepacia	J2315[T]	BCAL2281	BCAL0116	BCAL0117
			K56-2	BURCENK562V_C0873	BURCENK562V_C6829	BURCENK562V_C6827
			H111	I35_RS11045	I35_RS00620	I35_RS00625
			ST32	TQ36_07235	TQ36_13020	TQ36_13015
		B. cepacia	ATCC 25416[T]	QDC44_007384	QDC44_003576	QDC44_003577
		B. contaminans	LMG 23361[T]	-	-	-
			MS14	-	-	-
			R-71171	-	BCO71171_04328	BCO71171_04329
			AU10235	-	C6P88_37635	C6P88_37625
			AuBur16	K8353_16005	K8353_07600	K8353_07605
		B. diffusa	AU 1075[T]	-	-	-
		B. dolosa	AU 0645[T]	-	AK34_2911	AK34_2910
		B. lata	383[T]	BCEP18194_RS17695	BCEP18194_RS06780	BCEP18194_RS06785
		B. latens	CCUG 54555[T,b]	-	F7R21_31455	F7R21_31450
		B. metallica	LMG 24068[T]	-	-	-
			UMG646	FCJ61_27035	-	FCJ61_05780
			UMC708	-		FCJ60_23340
		B. multivorans	ATCC BAA-247[T]	BURMUCF1_2167	-	BURMUCF1_3486
		B. orbicola	AU 1054	BCEN_RS29065	BCEN_RS14305	BCEN_RS14300**
			PC184	BCPG_02526	BCPG_01509	BCPG_01510
			HI2424	Bcen2424_2186	Bcen2424_0243	Bcen2424_0244
			MC0-3	-	Bcenmc03_0225	Bcenmc03_0226
		B. paludis	MSh1[T]	GQ56_0128925	GQ56_0101910	GQ56_0101905

	B. pseudomultivorans	SUB-INT23-BP2	-	WS57_RS13795	WS57_RS13800
	B. puraquae	CAMPA 1040[T]	-	-	-
	B. pyrrocinia	DSM 10685[T,c]	-	-	-
	B. reimsis	BE51[T]	-	DPV79_37150	DPV79_37140
	B. seminalis	LMG 24067[T]	BSE24067_05326	-	BSE24067_04695
		Bp8988	DF032_34585	-	DF032_36540
		AU3273	LGN30_22985	-	LGN30_29255
		AU2283	LGN23_05100	-	LGN23_28865
		BC00018	I7824_32460	-	I7824_17880
		BC00027	I5745_31205	-	I5745_11810
		869T2	-	-	DT99_001015
		FL-5-4-10-S1-D7	-	-	WJ12_RS00875
	B. stabilis	ATCC BAA-67[T]	BBJ41_18070	BBJ41_RS10855	BBJ41_RS10860
	B. stagnalis	MSMB735WGS	-	WT74_RS01140	WT74_RS01145
	B. territorii	A63	-	BZY94_36395	BZY94_36385
	B. ubonensis	MSMB1189WGS[d]	-	WK67_RS04365	WK67_RS04370
	B. vietnamiensis	TVV75[T]	-	-	-
BPC	*B. humptydooensis*	MSMB43[T]	-	-	-
	B. mayonis	BDU6[T]	-	-	-
	B. oklahomensis	C6786[T]	-	-	-
	B. pseudomallei	K96243[T]	-	-	-
	B. savannae	MSMB266[T]	-	-	-
	B. singularis	LMG 28154[T]	-	-	-
	B. thailandensis	E264[T]	-	-	-
PPC	*B. gladioli*	ATCC 10248[T]	BM43_6624	-	-
	B. glumae	LMG 2196[T]	KS03_3720***	-	-
	B. perseverans	INN12[T]	BpN12_RS14015	-	-
	B. plantarii	ATCC 43733[T]	bpln_RS32110	-	-

Paraburkholderia	*P. caledonica*	W50D[T]	NA	NA	NA
		DS1039	-	J2776_001575	J2776_001576
		DS1061	-	J2793_000688	J2793_000689
		PHRS4	-	CUJ87_01715	CUJ87_01710
	P. caribensis	MWAP64[T]	-	-	AN416_RS00175
		PCAR477	PCAR4_80036	-	PCAR4_180102
	P. fungorum	ATCC BAA 463[T]	-	OI25_1096	OI25_1097
		SEMIA 4007	-	GGD68_007346	GGD68_007345
		OTU2BAGNBA1	-	NFE55_02990	NFE55_02985
		ANT-6	OSB38_39685	OSB38_05475	OSB38_05470
	P. gardini	LMG 32171[T]	-	R54767_03116	R54767_03117
	P. graminis	LMG 18948[T]	NA	NA	NA
		PHS1	-	CUJ91_01990	CUJ91_01985
		C4D1M	-	R8871_02285	R8871_02286
	P. kururiensis	KP23[T,e]	NA	NA	NA
	P. phenazinium	LMG 2247[T]	NA	SAMN05216466_109234	SAMN05216466_109233
		GAS86	NA	SAMN05444168_2260	SAMN05444168_2261
		GAS95	SAMN05444165_4936	SAMN05444165_4647	SAMN05444165_4646
	P. phymatum	STM815[T]	-	-	Bphy_0337
	P. phytofirmans	PsJN[T]	-	-	Bphyt_0607
	P. sabiae	LMG 24235[T]	QEN71_29690	QEN71_01815	QEN71_01810
	P. silvatlantica	SRMrh-20[T]	NA	NA	NA
		SRMrh-85	-	-	FHX59_003367
	P. terrae	DSM 17804[T]	-	-	C2L65_01675
		KU-15	-	-	PTKU15_03230
		KU-64	-	-	PTKU64_03210
	P. terricola	LMG 20594[T]	-	SAMN05192548_1014145	SAMN05192548_1014144
		mHS1	-	CUJ90_01870	CUJ90_01865
	P. unamae	MTI-641[T]	NA	NA	NA
		SCZa-39	C7402_10442	C7402_10430	NA
		CATux-332	C7401_103397	C7401_103157	C7401_104368
		PUN457	PUN4_660028	PUN4_590008	PUN4_100011
	P. xenovorans	LB400[T]	-	-	Bxe_A4111

Caballeronia	*C. choica*	LMG 22940[T]	-	AWB68_05906	AWB68_05907
	C. cordobensis	LMG 27620[T]	AWB70_03009	-	AWB70_03008
	C. glathei	AB V[T]	NA	NA	NA
		LMG 14190	BGLT_04383	-	-
	C. grimmiae	R27[T]	-	BG57_30900	BG57_05355
	C. humi	LMG 22934[T]	AWB65_01214	-	-
	C. jiangsuensis	MP-1[T]	BG58_26510	-	BG58_26505
	C. mineralivorans	PML1(12) [T]	-	-	EOS_23765
	C. novocaledonica	LMG 28615[T]	NOV72_00077	NOV72_00075	NOV72_00076
	C. sordidicola	LMG 22029[T]	AWB64_03051	-	AWB64_01872
	C. telluris	LMG 22936[T]	AWB66_03347	-	AWB66_01685
	C. terrestris	LMG 22937[T]	AWB67_01907	-	AWB67_03043
	C. udeis	LMG 27134[T]	-	-	-
	C. zhejiangensis	OP-1[T]	BG60_15320	BG60_15310	BG60_15315
Trinickia	*T. caryophilli*	720[T]	-	C0Z17_28435	C0Z17_28430
	T. dinghuensis	DHOM06[T]	DWV00_33035	-	-
	T. fusca	7MK8-2[T]	-	D7S89_23560	D7S89_23565
	T. soli	GP25-8[T]	-	-	-
	T. symbiotica	LMG 26032[T]	-	-	-
	T. terrae	7GSK02[T]	-	-	-
	T. violacea	DHOD12[T]	-	FAZ95_32945	FAZ95_30970
Robbsia	*R. andropogonis*	ICMP2807[T]	WM40_22060	-	-
Pararobbsia	*P. alpina*	LMG 28138[T]	-	-	-
	P. silviterrae	DHC34[T]	-	-	-
Mycetohabitans	*M. endofungorum*	HKI456[T]	-	-	-
	M. rhizoxinica	HKI454[T]	-	-	-

[a]All species are members of *Burkholderia* sensu lato (20).
[b]FhuA and FhuB orthologues are encoded by the genome of the *B. latens* type strain but not by the three sequenced strains AU0505, AU2934 and AU17928.
[c]FhuA and FhuB orthologues are encoded in one strain identified as *B. pyrrocinia* (MSMB1755).

[d]FhuA and FhuB orthologues are not encoded in some *B. ubonensis* strains such as MSMB22 and MSMB153.
[e]FhuA and FhuB orthologues are encoded by some *P. kururiensis* strains.
-, not identified in the indicated strain or species unless indicated otherwise. In some cases, this may be due to incomplete coverage of the genome.
NA, genome sequence data unavailable.
*, annotated as a pseudogene in the type strain, but intact in other strains such as 1CH1.
**, annotated as a pseudogene.
***, annotated as a pseudogene in the type strain, but intact in some strains such as AU6208 (locus tag GQR88_RS27760).
[T]Type strain.
Abbreviations: BCC, Burkholderia cepacia complex; BPC, Burkholderia pseudomallei complex; PPC, phytopathogenic clade.

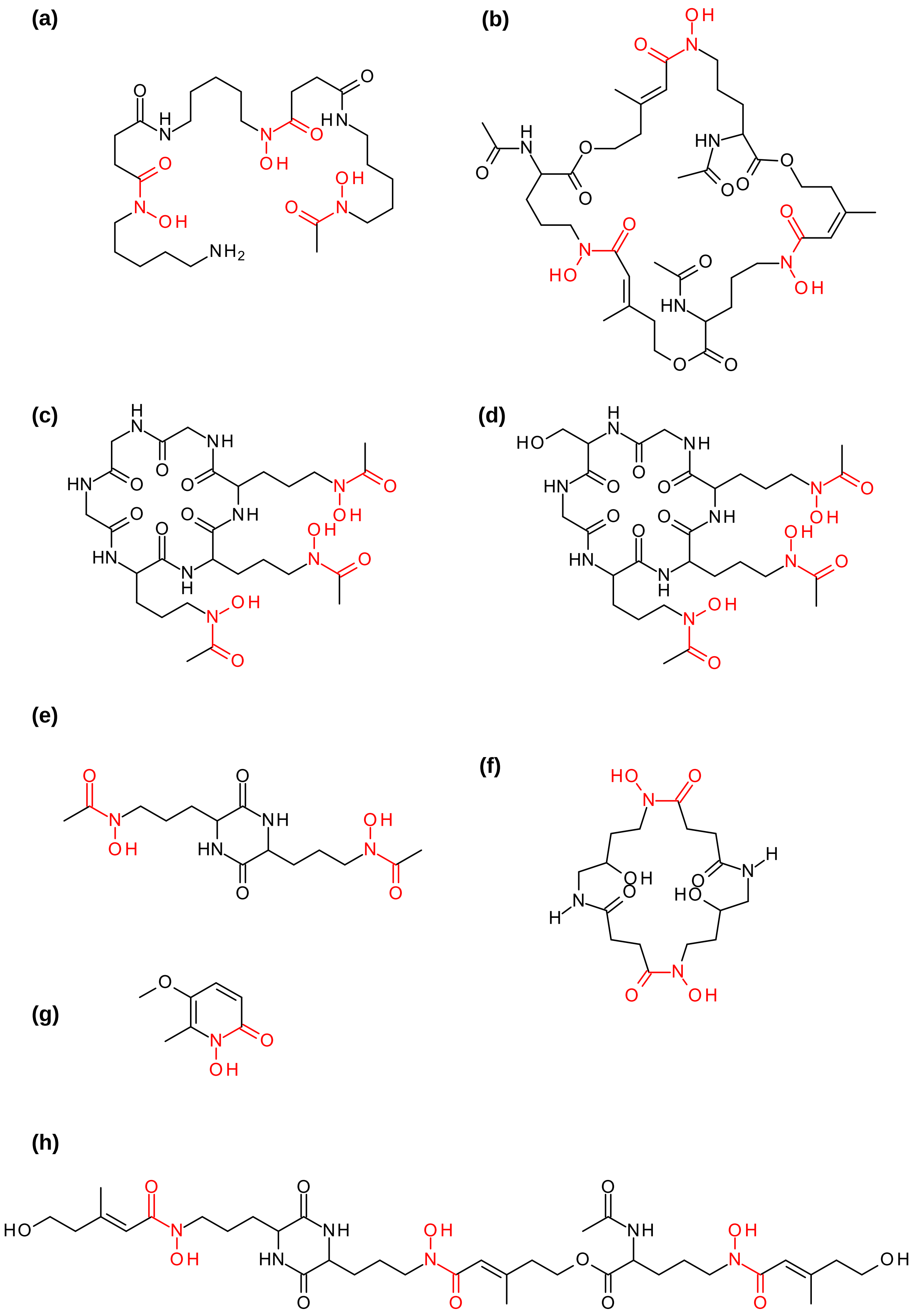
(a)
(b)
(c)
(d)
(e)
(f)
(g)
(h)

Fig. S1. Structures of hydroxamate siderophores. (a) Ferrioxamine B, (b) triacetylfusarinine C, (c) ferrichrome, (d) ferricrocin, (e) rhodotorulic acid, (f) alcaligin, (g) cepabactin, (h) coprogen. Hydroxamate groups are shown in red. Chemical structures were drawn using BIOVIA Draw.

(a)

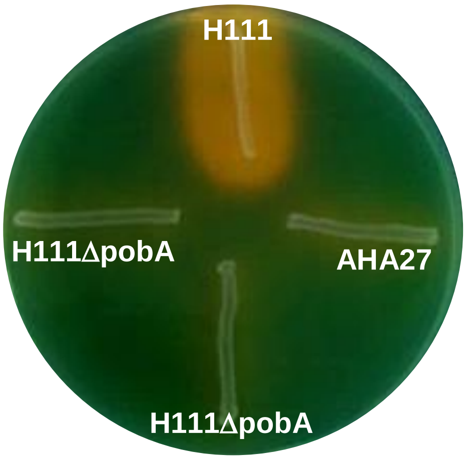

(b)

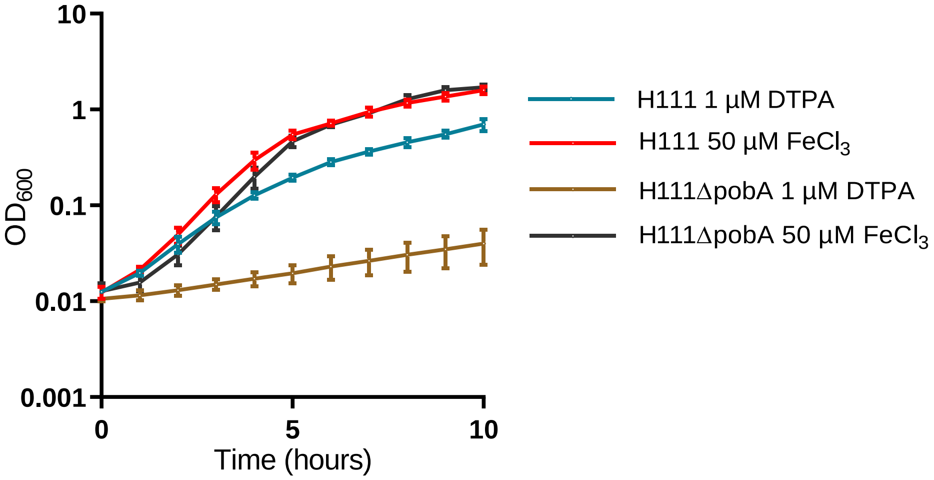

Fig. S2. Characterisation of a *B. cenocepacia pobA* deletion mutant. (a) CAS agar assay of siderophore production by the H111 wild type strain and *pobA* mutant derivatives of strains H111 (H111ΔpobA) and 715j (AHA27). (b) Comparison of the growth of H111 and H111ΔpobA in iron starvation medium and iron replete medium. Data were derived from three independent experiments (n=3). Error bars represent the ±SD.

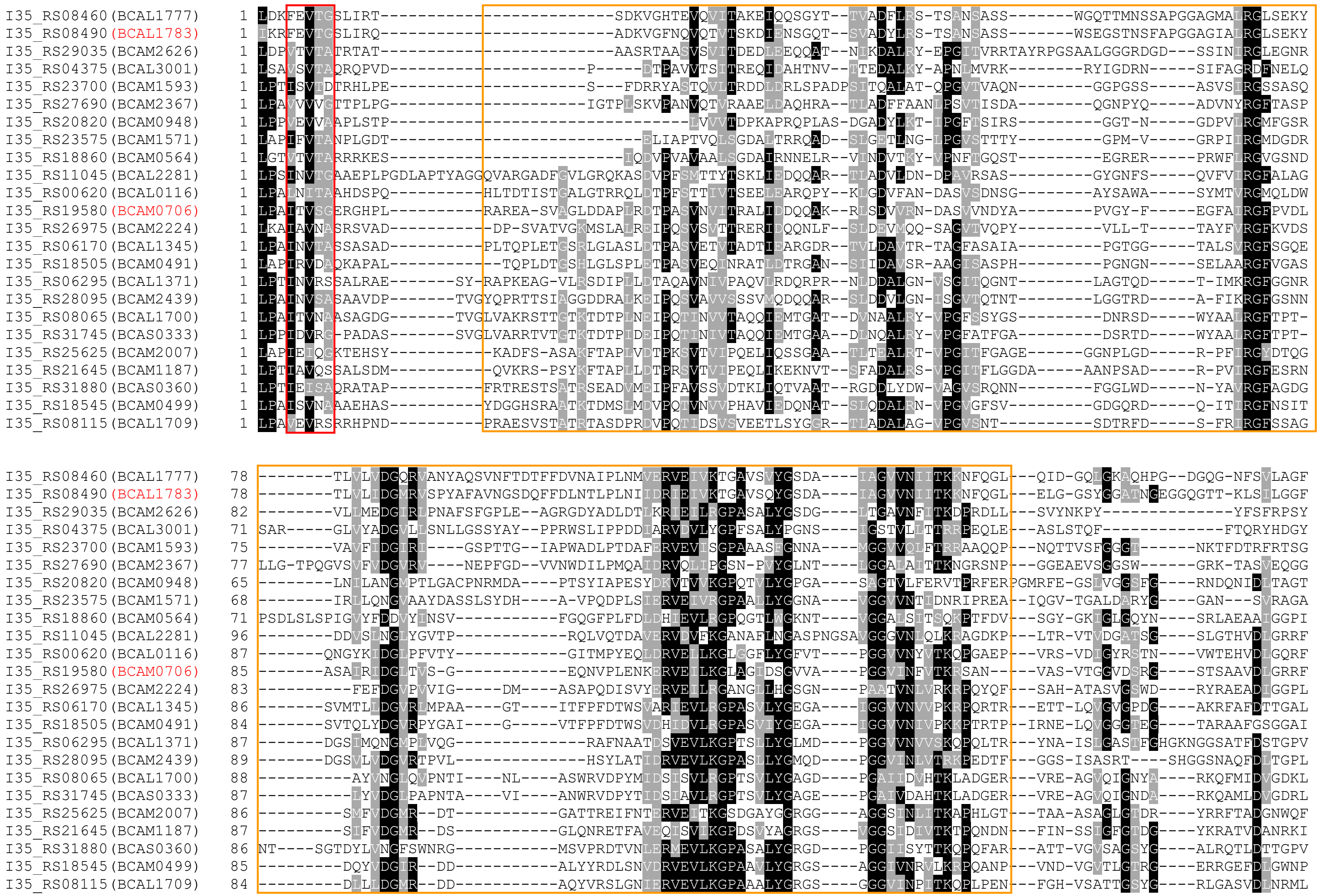
I35_RS08460(BCAL1777) 1 LDKFEVTGSLIRT----------------------------SDKVGHTEVQVITAKEIQQSGYT--TVADFLRS-TSANSASS-------WGQTTMNSSAPGGAGMAIRGLSEKY
I35_RS08490(BCAL1783) 1 IKRFEVTGSLIRQ----------------------------ADKVGFNQVQTVTSKDIENSGQT--SVADYLRS-TSANSASS-------WSEGSTNSFAPGGAGIAIRGLSEKY
I35_RS29035(BCAM2626) 1 LDPVTVTATRTAT----------------------------AASRTAASVSVITDEDLEEQQAT--NIKDALRY-EPGITVRRTAYRPGSAALGGGRDGD---SSINIRGLEGNR
I35_RS04375(BCAL3001) 1 LSAVSVTAQRQPVD------------------------P---DTPAVVTSITREQIDAHTNV--TTEDALKY-APNIMVRK--------RYIGDRN-----SIFAGRDFNELQ
I35_RS23700(BCAM1593) 1 LPTISVTDTRHLPE------------------------S---FDRRYASTQVLTRDDLDRLSPADPSITQALAT-QPGVTVAQN--------GGPGSS-----ASVSIRGSSASQ
I35_RS27690(BCAM2367) 1 LPAVVVVGTTPLPG--------------------IGTPLSKVPANVQTVRAAELDAQHRA--TLADFFAANLPSVTISDA--------QGNPYQ-----ADVNYRGFTASP
I35_RS20820(BCAM0948) 1 LPPVEVVAAPLSTP----------------------------------LVVVTDPKAPRQPLAS-DGADYLKT-IPGFTSIRS---------GGT-N-----GDPVLRGMFGSR
I35_RS23575(BCAM1571) 1 LAPIFVTANPLGDT----------------------------ELIAPTVQLSGDALTRRQAD--SLGETLNG-IPGVSTTTY---------GPM-V-----GRPIIRGMDGDR
I35_RS18860(BCAM0564) 1 LGTVTVTARRRKES--------------------------IQDVPVAVAALSGDAIRNNELR--VINDVTKY-VPNFIGQST---------EGRER-----PRWFLRGVGSND
I35_RS11045(BCAL2281) 1 LPSINVTGAAEPLPGDLAPTYAGGQVARGADFGVLGRQKASDVPFSMTTYTSKLIEDQQAR--TLADVLDN-DPAVRSAS---------GYGNFS-----QVFVIRGFALAG
I35_RS00620(BCAL0116) 1 LPALNITAAHDSPQ----------HLTDTISTGALGTRRQLDTPFSTTIVTSEELEARQPY--KLGDVFAN-DASVSDNSG---------AYSAWA-----SYMTVRGMQLDW
I35_RS19580(BCAM0706) 1 LPAITVSGERGHPL----------RAREA-SVAGLGDDAPLRDTPASVNVITRALIDDQQAK--RLSDVVRN-DASVNDYA---------PVGY-F-----EGFAIRGFPVDL
I35_RS26975(BCAM2224) 1 LKAIAVNASRSVAD----------DP-SVATVGKMSLALREIPQSVSVTTRERIDQQNLF--SLDEVMQQ-SAGVTVQPY---------VLL-T-----TAYFVRGFKVDS
I35_RS06170(BCAL1345) 1 LPAINVTASSASAD----------PLTQPLETGSRLGLASLDTPASVETVTADTIEARGDR--TVLDAVTR-TAGFASAIA---------PGTGG-----TALSVRGFSGQE
I35_RS18505(BCAM0491) 1 LAPIRVDAQKAPAL-----------TQPLDTGSHLGLSPLETPASVEQINRATLDTRGAN--SIIDAVSR-AAGISASPH---------PGNGN-----SELAARGFVGAS
I35_RS06295(BCAL1371) 1 LPTINVRSSALRAE-------SY-RAPKEAG-VLRSDIPLLDTAQAVNIVPAQVLRDQRPR--NLDDALGN-VSGITQGNT--------LAGTQD-----T-IMKRGFGGNR
I35_RS28095(BCAM2439) 1 LPAINVSASAAVDP-------TVGYQPRTTSIAGGDDRALKEIPQSVAVVSSSVMQDQQAR--SLDDVLGN-ISGVTQTNT--------LGGTRD-----A-FIKRGFGSNN
I35_RS08065(BCAL1700) 1 LPAITVNAASAGDG-------TVGLVAKRSTTGTKTDTPLNEIPQTINVVTAQQIEMTGAT--DVNAALRY-VPGFSSYGS---------DNRSD-----WYAALRGFTPT-
I35_RS31745(BCAS0333) 1 LPPIDVRG-PADAS-------SVGLVARRTVTGTKTDTPIDEIPQTINIVTAQQIEMTGAA--DLNQALRY-VPGFATFGA---------DSRTD-----WYAALRGFTPT-
I35_RS25625(BCAM2007) 1 LAPIEIQGKTEHSY----------KADFS-ASAKFTAPLVDTPKSVTVIPQELIQSSGAA--TLTEALRT-VPGITFGAGE------GGNPLGD-----R-PFIRGYDTQG
I35_RS21645(BCAM1187) 1 LPTIAVQSSALSDM----------QVKRS-PSYKFTAPLLDTPRSVTVIPEQLIKEKNVT--SFADALRS-VPGITFLGGDA-----AANPSAD-----R-PVIRGFESRN
I35_RS31880(BCAS0360) 1 LPTIEISAQRATAP----------FRTRESTSATRSEADVMEIPFAVSSVDTKLIQTVAAT--RGDDLYDW-VAGVSRQNN--------FGGLWD-----N-YAVRGFAGDG
I35_RS18545(BCAM0499) 1 LPAISVNAAAEHAS----------YDGGHSRAATKTDMSLMDVPQTVNVVPHAVIEDQNAT--SLQDALRN-VPGVGFSV---------GDGQRD-----Q-ITIRGFNSIT
I35_RS08115(BCAL1709) 1 LPAVEVRSRRHPND----------PRAESVSTATRTASDPRDVPQTIDSVSVEETLSYGGR--TLADALAG-VPGVSNT-----------SDTRFD-----S-FRIRGFSSAG
I35_RS08460(BCAL1777) 78 --------TLVLVDGQRVANYAQSVNFTDTFFDVNAIPLNMVERVEIVKTGAVSVYGSDA----IAGVVNIITKKNFQGL---QID-GQLGKAQHPG--DGQG-NFSVLAGF
I35_RS08490(BCAL1783) 78 --------TLVLIDGMRVSPYAFAVNGSDQFFDLNTLPLNIIDRIEIVKTGAVSQYGSDA----IAGVVNIITKKNFQGL---ELG-GSYGGATNGEGGQGTT-KLSILGGF
I35_RS29035(BCAM2626) 82 --------VLLMEDGIRLPNAFSFGPLE--AGRGDYADLDTLKRIEILRGPASALYGSDG----LTGAVNFITKDPRDLL---SVYNKPY--------------YFSFRPSY
I35_RS04375(BCAL3001) 71 SAR-----GLVYADGVLLSNLLGSSYAY--PPRWSLIPPDDIARVDVLYGPFSALYPGNS----IGSTVLLTTRRPEQLE---ASLSTQF---------------FTQRYHDGY
I35_RS23700(BCAM1593) 75 --------VAVFIDGIRI----GSPTTG--IAPWADLPTDAFERVEVISGPAAASFGNNA----MGGVVQIFTRRAAQQP---NQTTVSFGGGT-------NKTFDTRFRTSG
I35_RS27690(BCAM2367) 77 LLG-TPQGVSVFVDGVRV----NEPFGD--VVNWDILPMQAIDRVQLIPGSN-PVYGLNT----LGGALAITTKNGRSNP---GGEAEVSGGSW------GRK-TASVEQGG
I35_RS20820(BCAM0948) 65 --------LNILANGMPTLGACPNRMDA----PTSYIAPESYDKVTVVKGPQTVLYGPGA----SAGTVLFERVTPRFERPGMRFE-GSLVGGSFG----RNDQNIDLTAGT
I35_RS23575(BCAM1571) 68 --------IRILQNGVAAYDASSLSYDH----A-VPQDPLSIERVEIVRGPAALLYGGNA----VGGVVNTIDNRIPREA--IQGV-TGALDARYG----GAN---SVRAGA
I35_RS18860(BCAM0564) 71 PSDLSLSPIGVYFDDVYINSV-----------FGQGFPLFDLDHIEVLRGPQGTLWGKNT----VGGALSITSQKPTFDV---SGY-GKIGLGQYN----SRLAEAAIGGPI
I35_RS11045(BCAL2281) 96 --------DDVSLNGLYGVTP-----------RQLVQTDAVERVDVFKGANAFLNGASPNGSAVGGVNLQLKRAGDKP---LTR-VTVDGATSG----SLGTHVDIGRRF
I35_RS00620(BCAL0116) 87 -------QNGYKIDGLPFVTY-----------GITMPYEQLDRVEILKGLGGFLYGFVT----PGGVVNYVTKQPGAEP---VRS-VDIGYRSTN----VWTEHVDVGQRF
I35_RS19580(BCAM0706) 85 -------ASAIRIDGLTVS-G-----------EQNVPLENKERVEILKGLAGIDSGVVA----PGGVINFVTKRSAN-----VAS-VTGGVDSRG----STSAAVDIGRRF
I35_RS26975(BCAM2224) 83 ---------FEFDGVPVVIG-----DM----ASAPQDISVYERVEILRGANGLLHGSGN----PGGVVNLVRKRPQYQF---SAH-ATASVGSWD----RYRAEADIGGPL
I35_RS06170(BCAL1345) 86 -------SVMTLLDGVRLMPAA----GT----ITFPFDTWSVARIEVLRGPASVLYGEGA----PAATVNLVRKRPQRTR---ETT-LQVGVGPDG----AKRFAFDTTGAL
I35_RS18505(BCAM0491) 84 -------SVTQLYDGVRPYGAI----G-----VTFPFDTWSVDHIDVLRGPASVIYGEGA----IGGVVNIVPKKPTRTP---IRNE-LQVGGGTEG----TARAAFGSGGAI
I35_RS06295(BCAL1371) 87 -------DGSIMQNGMPLVQG-------------RAFNAATDSVEVLKGPTSLLYGLMD----PGGVVNVVSKQPQLTR---YNA-ISLGASTFGHGKNGGSATFDSTGPV
I35_RS28095(BCAM2439) 89 -------DGSVLVDGVRTPVL-------------HSYLATIDRVEVLKGPASLLYGMQD----PGGVINLVTRKPEDTF---GGS-ISASRT----SHGGSNAQFDITGPL
I35_RS08065(BCAL1700) 88 ----------AYVNGLQVPNTI----NL----ASWRVDPYMIDSISVLRGPTSVLYGAGD----PGAIIDVHTKLADGER---VRE-AGVQIGNYA----RKQFMIDVGDKL
I35_RS31745(BCAS0333) 87 ----------IYVDGLPAPNTA----VI----ANWRVDPYTIDSIAVLRGPTSVLYGAGE----PGAIVDAHTKLADGER---VRE-AGVQIGNDA----RKQAMLDVGDRL
I35_RS25625(BCAM2007) 86 ---------SVFVDGMR--DT-----------GATTREIFNTERVEITKGSDGAYGGRGG----AGGSINLITKAPHLGT---TAA-ASAGLGTDR----YRRFTADGNWQF
I35_RS21645(BCAM1187) 87 ---------SIFVDGMR--DS-----------GLQNRETFAVEQISVIKGPDSVYAGRGS----VGGSIDIVTKTPQNDN---FIN-SSIGFGIDG----YKRATVDANRKI
I35_RS31880(BCAS0360) 86 NT----SGTDYLVNGFSWNRG-----------MSVPRDTVNLERMEVLKGPASALYGRGD----VGGSIDIVTKQPQFAR---ATT-VGVSAGSYG----ALRQTLDTTGPV
I35_RS18545(BCAM0499) 85 ---------DQYVDGIR--DD-----------ALYYRDLSNVDRVEVLKGPAAVLYGRGS----AGGIIVNRVLKRPQANP---VND-VGVTLGTRG----ERRGEFDIGWNP
I35_RS08115(BCAL1709) 84 ---------DLLIDGMR--DD-----------AQYVRSLGNIERVEVLKGPAAALYGRGS----GGGVINPITKQPLPEN---FGH-VSATTGSYG----RLGASVDLNRML

```
I35_RS08460 (BCAL1777)  171 G---------DLNSDRFNVTAAASYYRDSGSTLG-DRDMTSAQDFTQ-------Y-PGGLAAPLGPNQ-QSYWS-LADGSRVPLSPCPPGSKTS-----------ATNCTYN
I35_RS08490 (BCAL1783)  173 G---------DLNADRFNVTAALSYYKSNGISIA-DRDTTQNQNFSN-------F-PGGLSNQ----S-VSYWRNPATGAKVPLSPCPNGGQAVPGTSVAQVNGPGTVCANN
I35_RS29035 (BCAM2626)  163 D-----------------STDR---SIGATVSAAGGN--D--------------R-VQGMIIADGRRG-HE--------------VDTRGGNNT--------------ASTL
I35_RS04375 (BCAL3001)  156 G-----------------FADSL-------------GGNHQTARIAN-------R-VGRFWFALSLDR-LE----------------NNG-------------------QPM
I35_RS23700 (BCAM1593)  160 T-----------------VPS------------TGPL---------A-------A-LGGLTYSLGL----------------------------------------------
I35_RS27690 (BCAM2367)  167 T-----------------IGSNLDYYATANVANDGGWADHNASRVRQ-------A-FGKLRYTDADTT--------------------------------------------
I35_RS20820 (BCAM0948)  156 P---DVYGRVTANHAHSQDYQDG-----NG-NT---VP-SQWDKW---------NADAALGWTPDDHTRVE------------L-------------------------TA-
I35_RS23575 (BCAM1571)  153 A---QVEGGNGRFAFHVDAFDRE-----TS-KL---RI-PGYARSSQQRAIDGPDTPQPEGNVPNSDGRVH------------GG--------------------AVGASY-
I35_RS18860 (BCAM0564)  160 G-------KNDVLAARVSVYHEN-----ADSFY---TNTVQQGRFGG-------FHDNAVRFQVLAVPTSD------------TD--------------------FLFNIHG
I35_RS11045 (BCAL2281)  180 G-------SEGQFGVRVNQSISG-----GD-TA---VDDERRRSNVT-------A--VSLDWRGDT-LRLS------------GD--------------------FLYQRQR
I35_RS00620 (BCAL0116)  168 G-------PDHMFGARLNATHEE-----GK-TY---NDGNIRRDSVS----------LALQANLTRDLSVS------------FG--------------------ALYQDRR
I35_RS19580 (BCAM0706)  163 G-------PDHQFGFRINAAKEN-----MH-SY---IDGTNGRRTFG-------S--IAADWDISPRASLQ------------LN--------------------AEFQQWI
I35_RS26975 (BCAM2224)  164 N-------AAGTVRSRLVAAYED-----RH-FF---YDHAKQDTRSI-------Y--SVTEVDVTRDTLLT------------FG--------------------AQYQTTS
I35_RS06170 (BCAL1345)  171 G---------PRLSYRFYASDAR-----AN-GL---AERADTHTTAI-------G--GALTFDVSPRLTLT------------LD--------------------YDYGRQM
I35_RS18505 (BCAM0491)  169 N---------DKLSYRFDVSGNR-----SS-NW---VDRGDSRDLSV-------S--GALRYDVTPDLYVT------------AS--------------------YAQGFMH
I35_RS06295 (BCAL1371)  170 G--------DSRLAYRLIVDQSN-----EQ-YW---RNFGEYRQTFV-------A--PSLAWY-GRDTQVA------------VS--------------------YQYRKFH
I35_RS28095 (BCAM2439)  168 GRPGQV--AGGTLAFRLTGEYDT-----SR-YW---RSFGRERNALI-------A--PALSWH-DANTSID------------VS--------------------YQYVDYT
I35_RS08065 (BCAL1700)  170 D-------PDGKYAYRFVGVARD-----GN-AL---TGPNNDQRVAL-------A--PSFRWRPDADTSLT------------LS--------------------ATYLQDW
I35_RS31745 (BCAS0333)  169 D-------PDGRYAYRFVGVARD-----GN-AV---TGPNGDRRVAL-------A--PSFRWRPNADTSLT------------FS--------------------ATFLQDS
I35_RS25625 (BCAM2007)  164 A---------DHAAFRLNLMSHN-----NDVAG---RDAVNNERWGV-------A--PSIAFGLGTSTRVT------------AS--------------------YYHLQTD
I35_RS21645 (BCAM1187)  165 N---------DTTAVRLNVMGHD-----ANQAG---RNDVYNKRWGV-------A--PSIVFGLNTPTTVT------------VS--------------------YYHMNSY
I35_RS31880 (BCAS0360)  171 T---------QSLAYRFVAMNEN-----NG-SF---RDTVSSKRYLF-------S--PSFTWDIGADTTLH------------YE--------------------FESARQR
I35_RS18545 (BCAM0499)  163 N---------DAARFRMTGAAEN-----SN-SF---RDRFQLNRQAI-------A--PSAQFKLDRDTVLN------------VE--------------------FDYLHDR
I35_RS08115 (BCAL1709)  162 S---------SAWSMRLNAGREH-----AG-SF---RDHVDGTRQFV-------A--PSIKWHDARRS-WL------------LQ--------------------FEYDTYR

I35_RS08460 (BCAL1777)  252 PAASTSLVPSTTRLNAKVRATFKIDDNTQAYAGFWVSRDETVQLQGPASISSTTNVYNPSTGSVSPLPRTV----PVSNPYNPFGVPTAINLTFPGNVVGADTVSTFWMANT
I35_RS08490 (BCAL1783)  262 TAYASSLSPWTERLSAKVHADFKITDAMQAFADLWESNNTTVTNNFVGRLGNSTQTYDPATGGLTPISNLV----PGSNPYNPFGVPAKLVYAFPATQ-AVHTSSNFWRAAT
I35_RS29035 (BCAM2626)  109 RTTSNPQDVYSESLLGKL-------VLTPT-------------------------TRDTIKFTAETVQRRVST-----DVLSAINAPTTLGLTTHD----------RLERNR
I35_RS04375 (BCAL3001)  194 -QYASPASAYNPRLGAAVPVTGAATDIGPN-------------------------GKPRTIVGAQTIERTEQLNE-----------TVRMGYAFTDHVDATLTLGHWENHYR
I35_RS23700 (BCAM1593)  180 --------------------------------------------------------HDYNTAGIDATRPFFYGHEDGRNPYHAQDLDARLGYARDNWSISTFALYHRSDLSY
I35_RS27690 (BCAM2367)  210 ------------------------LSIAAG-------------------------GADNTLTGTQTIPRSFLDNRKQ--A------------------------YTYPDRNR
I35_RS20820 (BCAM0948)  208 --------------------------------------------------------GTGDGYAR----------YAGRGMD---------------------GAHFRRETFG
I35_RS23575 (BCAM1571)  219 --------------------------------------------------------TWADGFAG----------LSYSGYESNYGSVAESD-----------VRLRMQERLA
I35_RS18860 (BCAM0564)  218 RNYTGG-GNAWHAEG-----------AGRG-------------------------GTNQFGFVGSSDPYTVSLNAPSSDHI------------------------STWG-TS
I35_RS11045 (BCAL2281)  234 IDNGRPIVLV----SGSPLPAV---------------------------------PSAT-HNYA----------QPWSFSE-L----------------------ED-TVGI
I35_RS00620 (BCAL0116)  222 TTGQTPSIFTGSYPGGALPATI---------------------------------SGGSTNLGG----------KD--QYL-N----------------------TNLQLYT
I35_RS19580 (BCAM0706)  218 QRSA-PG---YQLLGGTVVPSV---------------------------------KTTSKALGT----------QPWAKPV-T----------------------TDALNLN
I35_RS26975 (BCAM2224)  219 SVPDMSGVPM---ARDG----------SSL-------------------------GLSRSTFLD----------TAWGRFN------------------------WDTTRAF
I35_RS06170 (BCAL1345)  224 PATYYG-VPA---PNGVLDPSLRKL----------------------------------NYTVG----------DATISYY------------------------DQWTR--
I35_RS18505 (BCAM0491)  222 PMQYFG-APL---VDGARDRALDKK----------------------------------NYNVG----------DADIAFR------------------------DSWAT--
I35_RS06295 (BCAL1371)  223 SPFDRGTALD---PRTN-----APLDI------------------------------PARRRID----------EPFN----NM--------------------DGESHLAQ
I35_RS28095 (BCAM2439)  227 MPFDRGTVLV---NG-------RPDDA------------------------------LRYRRYE----------EAWS----QS--------------------SGIQETLR
I35_RS08065 (BCAL1700)  225 GDISSNFLPA---QGTVLPNP-------NG-------------------------QINKDIYEG----------DGNFNYYRK----------------------KQWS-IG
I35_RS31745 (BCAS0333)  224 GDISSNFLPA---SGTVLPNP-------NG-------------------------RLSQDLYMG----------DPSFNDYRK----------------------KQWS-LG
I35_RS25625 (BCAM2007)  218 DMPDGG-IPY---FYTTSNKPANVDTIYPA-------------------------PVDRHNFYG----------LIDRDFR-K----------------------TTSDIST
I35_RS21645 (BCAM1187)  219 DMPDFS-VPF---RAS---------GGTPV-------------------------PTDRGQFFG----------LNTRDYR-Y----------------------GQTDTGE
I35_RS31880 (BCAS0360)  215 APLDRG-VLA---VNGQLG------------------------------------VIPASRFLG----------EPRDGDY-----------------------DVRNTGHQ
I35_RS18545 (BCAM0499)  225 RTSDQG-LPA---YRG------RPVDV------------------------------PINAYYG----------SADGGNSSYN--------------------DISAKSAT
I35_RS08115 (BCAL1709)  214 RVPDRG-MPA---PVAAVDAAGKPLAFSLP-------------------------SAPRATFFG----------AAGRDTIRDE--------------------TMNWRS--
```

```
I35_RS08460(BCAL1777)  360 GVKGSFDTGRFGAWDWSADYGHSQSTVDTTYRNRINVAGLENMLANGTYNFSNPA--ATPNGLNGVFTDDD-------QQAIS-------------KVDSV-----------
I35_RS08490(BCAL1783)  369 GVKGSFSTPRVGDWDWTASYTHSQNTVSNNYSNLINAAALENIVQNGVFNFADPS--STPNGLNGLYGSTS-------TQAIT-------------KLDAV-----------
I35_RS29035(BCAM2626)  274 FSVDYDFRDDALRWFQTAH----V-----------------------QFYYQDAKQDQYAFETRGKLPSRS---------------RDNQ-----YKERAFGGA-AFAESGF
I35_RS04375(BCAL3001)  269 QHGETFLRDATGNPVYGGN----VSIGGQNMTVA--PN-----AFAPQRG--DQENWLYALGLNGRLDSGWRLSGVVSAYDVS---RDVL-----RAASTVQGGAGTLFQGD
I35_RS23700(BCAM1593)  236 DNS--GYANRELDH---------QLTTGVAFHLDITP---DT-QFDQSFGYANDRQFIYAD--NPALA----------TDQIN---SQR-----------ISTSTSLT--HQ
I35_RS27690(BCAM2367)  247 NSA--GYLTLSGERFFGEH----VELSGNAYYRHLRNTNTSS-NNNTDYGSVDEDGAIDTV--QGSNA-----------QSTIV---TDS-----------YGGSVQLTLLGK
I35_RS20820(BCAM0948)  233 L---SFDKRHLGDVLDRIEARVYYNEADH------------------VM------DNYTL--RQPDPASSMPM---RMA---------AD-----VRRRTVGAR---AAATF
I35_RS23575(BCAM1571)  255 L---ASEVRNLSGPFTTLKFDFAYTD-------------------------------YRH--KEIDNGET--------A---------TT-----FRNRGYEAR---IEARH
I35_RS18860(BCAM0564)  268 L---TAHWRINPAV--SLTSITAFEGLHR------------------WYQ----DDED----YSPVDAARSHD-----------RLSSRQ-----------------FSQEF
I35_RS11045(BCAL2281)  274 V---RAEYDFLPAW--TAYVSAGARHTNE----------------------HGDY---YTPTYSSSGTTG------------------SRLSVP-HKEDAQSAE-AGVRGRF
I35_RS00620(BCAL0116)  266 A---GLQYQLAPDW--QLDVAYSYSKATR----------------------RRNES----------------T---LYLQDAAGNYTDSRYVG-MEDHRFSQWR-AMVEGKV
I35_RS19580(BCAM0706)  260 A---RFDYQFNDDW--KAYIAAGRSRTMI----------------------DDNSAFAYGCSYAASCAAGATS---PFFFGANGDYDVYDFRSPGEYRRNDDLR-AVTTGKF
I35_RS26975(BCAM2224)  259 A---SVEQKLGAGW--KAKVSGEYQSVRS------------------DLK----YAGSFG-AIDPATGAGGRLTGAAY------QFSSYS----------RSLD-ANVQGPV
I35_RS06170(BCAL1345)  262 L---SASYRPTAGV--TIDNQLYYLTSN----------------------RHWRNAESYV--LDPATARVTRG---DYLDIGH---HQRQ----------IGDR-LSARFDG
I35_RS18505(BCAM0491)  260 V---SANWQPSDAL--NVTSTLYRMKSN----------------------RHWKDAEYYT--YLPSSAQVRRS---SYTEIFH---DQEQ----------YGNV-TTATVGS
I35_RS06295(BCAL1371)  263 L---SVDHQFNADW--SAHVGYSYNRET---------------------YD----ANQLRTTGVDPVKGTMTRS-----ND-ATHGSLSTD----------SYGI-GYVTGKL
I35_RS28095(BCAM2439)  265 T---RIEHRFSDAW--RVRATYGWGRDR---------------------YD----QFITRATAFNSRTGALTRS-----SD-ANLGRNDSD----------QIAT-LGLLGNV
I35_RS08065(BCAL1700)  269 Y---QFERNLTPAW--TFRQNTRLMHLS------------------------LDNASVFANGFAGDSLT----DVSRWAGLFQMNYSRFD----------IDN---NLEGRF
I35_RS31745(BCAS0333)  268 Y---ALEHRVNAIW--TLRQDVRWSHLS------------------------LDDATVFGNGLAPRSTT----NMMRFAGLFQLNYSRLD----------IDN---HAQARF
I35_RS25625(BCAM2007)  268 I---KIEHDITPNL--TVRNTTR---------------------------YTESTQDYIW--TQPDDSQGNVVNGKVWRRNNNRNSSINS----------LANL-TELFGEF
I35_RS21645(BCAM1187)  260 I---RVEHKLNDTW--KLKNTTM---------------------------FGRSTLDYVA--TNPQILASNPNMLG--LQAKSGKYALNG----------FSNQ-TEVTGSA
I35_RS31880(BCAS0360)  263 F---TLEHRIDSAW--SINAGVAQRNTD----------------------LSGRSSEAFA--LQPDGRTLWR----RY---RQVAFHSND----------LQGR-LETAGSF
I35_RS18545(BCAM0499)  258 V---SLDHRFNDSL--SFHGAIR------------------------AYDFSLERKNYVT--YEPIKTAAHPV---VTLDQSTRQRTDHG----------IDGL-FELTQKT
I35_RS08115(BCAL1709)  265 ----VFTHALDGDW--ELRHTLGVLDLRS------------------TFDNTYVTQSYVA--KPRDYRRVQRA---RYLQD----MTQLN----------VQTG-VELGGKV

I35_RS08460(BCAL1777)  439 -TAKAS---TSNLFTLPGGPVGL------GLGTEFRHES-STI---------NPQTLASQGVSAPAN-----------------------VQTVEGSRNVAAAFYQVDIPIL
I35_RS08490(BCAL1783)  448 -DATLS---TPNLFTLPTGNVGL------GLGTQFTHQS-QYI---------GTGADYANGTLIQPS-----------------------LQSVNGERNVAAVYYQIDIPIL
I35_RS29035(BCAM2626)  338 ATGPLA---HKLLVGVDGSLSRVT----------NLRDG-T-----------VPGVG--EAFPNKAF-----------------------P---DTDYTLFGAFVQDQIGYG
I35_RS04375(BCAL3001)  360 GTG-WR---TLDLKAEAPEVKGHTFTFGYHYDNYFLRNV-TYN---------TADWL--AG-PTTSL-----------------------ASVYRGDTRTQALFGQDAWRFA
I35_RS23700(BCAM1593)  305 AHG-FH------LFGLPL--SGES-----KLAYDFTREQ-AFL---------PVDIP--GGVPTR--------------------------------N-------DSAFSL-
I35_RS27690(BCAM2367)  326 LGG-MA---NRLVAGVSADVANSSYVAS-SQDASFTDAR-A-----------AIGIG--DFVPQT---------------------------SAKTRNANVGVYLSDALSLT
I35_RS20820(BCAM0948)  296 RFGDD----FKLVTGVDAQSNRL-------------DSR-SSM-------------GQQNYR-----------DQP---------------WDAQATMWNAGVFSELTWYAS
I35_RS23575(BCAM1571)  306 RKIGP----FEGAIGVQFGQNTF-----------------SAL-------------GDEMLV-------------------------------PSTRTNSVALFGLEEWQVV
I35_RS18860(BCAM0564)  321 RLESPQNDRLSWIVGTHLFTEQL---------------------AEQGAGGGLPGSPSPAYYH---------------------------LTDLTQHTQSAAIFGSVKYRFT
I35_RS11045(BCAL2281)  336 TTGPVS---HFVTAGASFIRIDS-------------QSAY----TMSNAFPTTLYDPAPVASPPTAYAG---GD--------------MNDPGTVTKTWLRSIAVSDTLGFL
I35_RS00620(BCAL0116)  330 RTGQFS---HQIVLGASWQKQAN-------------DYSANS--VFVPLGAGNLYAPNPYRYESPHG-F---IQ--------------Y----RTSEIVQKSLFASDTVQLT
I35_RS19580(BCAM0706)  341 ATGPLR---HELTLGVSVQRRVV-------------HM-ADA--VYDYVGSENVYGPDLTFSPSPNSPG---PS--------------Y----PRLDAWQYGVFGLDRISIG
I35_RS26975(BCAM2224)  326 HAFGLT---HDLLFGVTYANSSS-------------GQMTAPLLGDVAGTPVNVYRWNPSSVPEP-GI----------------GP---YQQSQQNDISQKGVYGLGRIKLA
I35_RS06170(BCAL1345)  328 MLFGRA---NRFVVGTEFSQTTF-------------SGT-NNS-PYGGETTVPVHGFDPGVFT------SPDPTV----------------PQFSTRARQAAVFAENRLEVL
I35_RS18505(BCAM0491)  326 ALFGMR---NTFSAGVEFNHTTF-------------QHD-NNS-PYAGTSTVDPFNVDPGSFI------NTAGTF----------------PKYRSQSNQYALFAENRLEIT
I35_RS06295(BCAL1371)  329 SLAGMR---HDVQVGFDTEYRRI-------------YRK-DMLRQAV---KTPFSYVDPVYGLLPPSS-TVSA----------------SDSDQTDTLHDASAFFQDTLHLT
I35_RS28095(BCAM2439)  331 TLAGMQ---HAIYVGGEYERQRS-------------FRG-DTIRGKA---TTGFNLYDPVYGLLAPGG-VPNP----------------KQSDSRSVVHAYSTIVQDSVKIT
I35_RS08065(BCAL1700)  335 ATGPLQ---HTLLLGFQYNRQTA-------------TDS-EWLAAAP-----TLNLYNPVYTPVTMGV--------------FSDPDATSRTNTYTTMNTFGLYAQDQIKW-
I35_RS31745(BCAS0333)  334 GTGPLE---HTLLFGAQFDRQTT-------------TNS-VWLALAP-----SLDLYHPVYRPVTAAI--------------FSGPTSLGHVDQYTAMNAFGVYAQDQIRW-
I35_RS25625(BCAM2007)  335 RTGPFK---HSFTTGIELSREWG-------------KRD-SYTVATDKGTICQKGIGAPSGYNCTSLW-SPNPNDPWAGSITRNNDYA------HARTTTKSIYGFDTIEIT
I35_RS21645(BCAM1187)  325 SLFGMK---HTMTAGVEFSHEQA-------------RYE-GYLVSDSAGNNIRSNSPCAVVGNCTPLAGGWNPNMPWTGSIVLNGDKGFPGATTNTRTDTVSAYIFDTVKLS
I35_RS31880(BCAS0360)  328 RTGGIG---HTLVMGVDAYRFNY-------------DQF-VTRSTPTAAAPYAIDIFDPVYG-----Q-PAPTPRTA--------------TNLLERDDGQGVYAQDTLAFG
I35_RS18545(BCAM0499)  325 SLFGMR---HELLYGLELSQQQK--------------F-DTIYSVTKVATYDLFNPQPVVL-----PGVPGGTRAK--------------TNASTVVGLAGVYAQDLISLT
I35_RS08115(BCAL1709)  333 ATGPAL---HHLLFGIEYGWQKR---------------T-PALWQADAA-PVPVTGPDRFAL-----VGST--PRPY--------------AMNRHRVSDYALFAQDRVDLG
```

```
I35_RS08460(BCAL1777)  508 RNLT-FTQAGRYDHYSDFG-------------------------------------------GAFSPSFALRFQPVQMLTTYASYSRGFRAPTLVENSQAVYLSHQNLVDPN
I35_RS08490(BCAL1783)  517 KNLT-FSQSGRYDHYSDFG-------------------------------------------GAFSPRFALRFQPIRELTMYGSFNRGFRAPTLIENTSSRTYGAQGAVDSN
I35_RS29035(BCAM2626)  397 -RLL-VTPGLRFDTYRLSP---------------TE------------NDPLFTGKAVSTSANELSPRVAVLYEITPAVIPYVQYAHGFRAPTPDQVNSS------------
I35_RS04375(BCAL3001)  432 PGWL-ATLGLRYERWDAYGGA-----LGNA----KG------------TLGYAD-----RSANALSPKVALQWDATEVWRFRLSFATGTRFPTVGELFQGT-----------
I35_RS23700(BCAM1593)  350 -HQS-ATLGSVTMFV-------------------AG------------RHEIVA---GQAV---NTGNAALSWAITPVYTARVSYGNAFRLPTFNDLYYPG-----------
I35_RS27690(BCAM2367)  392 PHWT-LTLSGRYDWSKAR--------IGDE----SG------------VQPLLD---GSHVFSRFNPAVGLNWNPVPGLTAYATYNEGMRSPTAIELACAD-----------
I35_RS20820(BCAM0948)  351 DVSR-VIGGARVDYASARDK----RAMKRG-MMMSKPNPT------------FDDDRTKVLPSGFVRYERD--LASLPVTWYAGIGHAERYPDYWELFSATRGPTGSVN---
I35_RS23575(BCAM1571)  353 PALK-LSLGGRFEHVK------------------VDPDPA----GV--E--KFAGAQPRDFNAGSLS-AGALFTLTPVWSIAANVAYTERAPTFYELYSNG--PHDATGQFL
I35_RS18860(BCAM0564)  385 DRFN-VTGGLRYTIERKTINLTGLQDTGN--VTFSDPNSWWSPSSVSSPLAVSAQQHQTNTWRAPTWDITPEYALSSNVRAYFRYARGFR-SGGYNG------------NAY
I35_RS11045(BCAL2281)  411 DDRVLFTLGARRQSISVDN----FDYTGAV-T-STY------------------------SNAITTPVFGLVVKPWRNVSIFANRSEALA-QGEIAP------------NT-
I35_RS00620(BCAL0116)  402 ERWS-VLAGVRYMNYAQRA----FQASGAE-DPGYR------------------------QNGVVTPTFAVMFKLAPTTTAYASYAESLE-PGSRVN------------DV-
I35_RS19580(BCAM0706)  413 EHWQ-VLAGGKEVLLRQRS----WSSLDGE-TT-HT------------------------DRSVFLPQVALVYKPVNVLSLYASYSKALS-LGDQAP------------VR-
I35_RS26975(BCAM2224)  402 EPLT-LVLGGRMSWWNQDS----L-------GAHYN------------------------TGHQFTPYGGLIWDFVRDWSWYASYAEVFQ-PQTKSM------------WG-
I35_RS06170(BCAL1345)  400 PRLA-WVSGLRYDHIAFSR-----EQAATG-AGFDK------------------------RFANIGWRTGFVFDIAPTFSAYAQYTTGAE-GVGSLV------------TLS
I35_RS18505(BCAM0491)  398 PRWS-VIGGLRYDHASVNR-----DDLVNG-GAFTK------------------------VFANTGWRIGTVYDVRPGLAVYGQYSVAAD-PVSSLL------------SLN
I35_RS06295(BCAL1371)  404 DKWI-VSGGIRYITYNQVA----GRGRPFT-ANTDL------------------------SGSKWLPRAGVVYKWTDSFSLYGSYSQSLK-PSSSIA------------PMT
I35_RS28095(BCAM2439)  406 DRLT-AVGGLRWENWQQES----GMGRPFV-FADRS------------------------RGSVWLPQFGLAYALTPALTAYANVSRSFK-PNVASN---------------
I35_RS08065(BCAL1700)  410 NRWT-LTLGGREDWVNMRQ-----DDRAAG-TSTKA------------------------DVTAFTGRVGLTYQGDYGLSPYISYATSFN-PLIGVN------------LL-
I35_RS31745(BCAS0333)  409 RRWT-LTLGGREDVVNARF-----DDRSAG-THVQQ------------------------DVSAFSGRVGLTYRGDAGWSPYVSYSTSFD-PVIGVR------------MF-
I35_RS25625(BCAM2007)  422 PRWQ-VNAGLRVDDYSTRF----TDTKANGGKTYTR------------------------DDTLFNWQAGLVFKPAQNGSIYASYATSST-PAGMLL-------------GE
I35_RS21645(BCAM1187)  420 ERWQ-FNTGLRFDRYDTTG-------KQAGVADLSN------------------------TSNLFSYQFGLVFKPVTNVSLYASYGTSSN-PPGSNG-------------GL
I35_RS31880(BCAS0360)  403 PHWK-ILAGLRWDRFHQSI-----DNRLKG-VTTSQ------------------------LQTALSPRIGVVYEMSPAWSLYANTAYSFR-PNNGAD------------VG-
I35_RS18545(BCAM0499)  399 EHWK-VLAGLRFDYLNQIR----HDYTSSN-VNLDR------------------------TDHAWSPRVGLIYEPLDWLTLYGSFSQSFS-PLADTL------------ISS
I35_RS08115(BCAL1709)  404 RAWK-LLGGLRAERFDVDS-----TNALNG-LHARR------------------------TTTAWSPRLGVVWSPVGAHSLYASYSKNFA-PVGGDL------------IGI

I35_RS08460(BCAL1777)  576 DPSGVPTKHFTTEQVAGNPNLQPEHTKNYNIGFQLSPD-AM---TDIGAAFYKVRIDGVIGTDDPNAVL-VANDPSRVVRNADGSVRYLVQHFVNLGAL-------DTDGFD
I35_RS08490(BCAL1783)  585 DPNNPNAYNLVEEVQTGNSKPQPERTKNYNIGFQLSPT-RN---TDFGFDWYKIHIDNVIGTEDIQTVI-DQNDPSKVIRNPNGTIAYVLLPYMNLSSL-------DTDGFE
I35_RS29035(BCAM2626)  468 -FSN-PV---YGYTSIGNPNLKPETSDTFEAGLRGKAG-TGYGIVRYSAAAFTGRYRNFISRTTI--------------AGSGRPVDPFVFQYVNFADA-------RIHGLE
I35_RS04375(BCAL3001)  506 -ISNNAI---VNN----NPNLRPEKAIDWDFTAERDVG--V---GVVRASVFQSDLRDSIYSQTT--------------ASGATT----VTNISNVDRV-------RVRGVE
I35_RS23700(BCAM1593)  413 -YGN--------------PSLSPERSTSVEAALDANT--AY---GTFTAAIYDTRVNNLIAYN----------------PATFS--------PMNIGRS-------HIRGID
I35_RS27690(BCAM2367)  465 -PAAPCS---LPNDFIADPPLEPVISKTFEAGMRGRIG-AA---TTWSAAAYRTTLTDDIQFI----------------SSPASA----QGYFRNVGDT-------RRQGIE
I35_RS20820(BCAM0948)  440 ----------------AFSAVRPEKTTQLDIGAQYKS--DR---F----DAWVSAYAGYVQDFILF---DYA-------TGMMGP-----TT-------QATNVNAQIMGGE
I35_RS23575(BCAM1571)  435 I---------------GNPNASKEKAVSTDLSLRYAS--GP---NRGSVGVFYNRFSNYLTEYNTGRVVDDD-------GEPVAP-----GTDGSLNEAIYRGVRAEFYGIE
I35_RS18860(BCAM0564)  481 -------------TQSTVSTVSPEYLSDYEVGIKSEWFDKR---LIVNASVFHYDYRD-------IQVFALA-------PNPFGG--PPVSTLSNAGQG-------RADGFE
I35_RS11045(BCAL2281)  479 -------------ARNAGQALSPYRSKQYEAGVRYDT--DK---YGASLALFQIEKPM-------AY-T-----------DP------ATNLFGADGTQ-------RHRGIE
I35_RS00620(BCAL0116)  470 -------------YANAGQVLKPLRSKQYELGIKSER--AR---WSATAALFRIERSA-------EY-A-----------N-------AANVYVQDGES-------IIQGIE
I35_RS19580(BCAM0706)  480 -------------ATNAYAFLPPVESHQFEVGAKYDWL-DR---LSLTAAVFSISKPF-------QF-A-----------DPDAS--GTSYTFVQRGTQ-------RHQGIE
I35_RS26975(BCAM2224)  464 -----------------GGILTPVKGRTYETGVKGELAGGK---LNVSLAAFRIDLDN-------NPQV-----------DLAHPCAGPSCYYVNGGSV-------RSQGFE
I35_RS06170(BCAL1345)  468 A---------------SQMNDRLATGAQWEAGLKQTLLDGR---AYWTVAVYDITKRN-------LLST-----------DPFNP-----ALRQQVGRQ-------SSRGVE
I35_RS18505(BCAM0491)  466 A---------------SKASFTLATGRQIEIGVKQSFLDGK---AEWTLAAYRIVKRN-------LLTA-----------DPVNP-----NQSIQVGQQ-------SSRGLE
I35_RS06295(BCAL1371)  473 G-------------YIIDGATPPEEATAWEVGGKLDLA-GG---MTGTLALFNIDKKN-------VLVS-----------QYNDA-TK-LTDWRTSGKA-------RSRGVE
I35_RS28095(BCAM2439)  472 ----------------VAAPLAPEYGRVLEAGLKFSLK-PA---ITGTLAVYQIDKRN-------VAVT-----------VDDI--------TSTIGTA-------RSRGIE
I35_RS08065(BCAL1700)  477 G----------------GGLPQPTRGKQIEAGLRWQPPGKN---LMLNAAIYQINQTN-------GITP-----------ALPSQDPG-GTKSVQSGEV-------RSRGIE
I35_RS31745(BCAS0333)  476 G----------------GGLPKPTRGVQTEAGLRWQPPGRN---LMLNAAVYQIDQTN-------VVTP-----------TPVNLDPT-ATTSVQTGKV-------RSRGIE
I35_RS25625(BCAM2007)  492 GSETQSLTPGRGGVGPNADQLSPEKNRSIELGTKWNVLNDK---LSLTAALFQIDTTN-------ARVT-----------LPNN-------QYAMVGNK-------RVQGLE
I35_RS21645(BCAM1187)  486 GGGTDQ-------ITATNQDLAPERARNIEIGAKWDVLQDQ---LSLTSALFQTEKTN-------ARVS-----------DGLG-------HTVNAGKQ-------RVRGFE
I35_RS31880(BCAS0360)  470 -----------------GRAFDPEKGHGYEAGAK--WAGAR---WLTTVSAFYVTKRN-------VLTA-----------DPANA-----GFSRAAGEV-------RSRGVE
I35_RS18545(BCAM0499)  468 G------------AFSNGAALAPQKTTAYEVGSRFDLG-GK---ATASVALFDMRQTN-------QQIG-----------DPANP-----GYALPIGTQ-------HVRGME
I35_RS08115(BCAL1709)  472 TPD----------ARGNANDLGPQYTRQYEIGVKSDWRDGA---LSTTLALFQLDLYN-------RRIA-----------DPVRP-----GFFDLTGLE-------RNRGLE
```

```
I35_RS08460(BCAL1777)  676 LNFRKALR----TKYGTFTLAGDWTYVWHFKLHSPGTAPQDFA-GNNLALLQPFGASNPRWK---GN--TSVSWDYR---Q-LTTTLTWQYTGPYTN-AVAAEF-GDGGTGS
I35_RS08490(BCAL1783)  685 TTFRQSVS----TKIGTFTLSGDWAYVWHFKMPVGG-ESTDFA-GNNGSLNEPFGGSFPRWK---GN--TSLNWNYH---NQWNATLTWMETGPYSQ-AILSPGQYPNMQDS
I35_RS29035(BCAM2626)  553 GRAE----WMMPNGITLKTAM-------AFTKGSTQ---ND----GAASQPLN------TVNPFSAV----FGVRYEPT-ERWFVQTDLLFQAAKRDKDVDKSDCSNKACFT
I35_RS04375(BCAL3001)  580 LAFSGENVG-------LRGLNLDANVS-----ASNA---QILA---DAANPAYVGSRFPRIPMRANLLAS----YRFD-EHWLASVGVRYSGRQYNT-LDNSDVNPDVYGG
I35_RS23700(BCAM1593)  474 LSYKGTIGRSTPVSIAVGILNPQDETNQTWLSRRPR---QT-------------------------VSLNVDHTWDELKL-HALSTGASLLYGGSTFD--------DPANRQY
I35_RS27690(BCAM2367)  542 LAGRTRVG---PLGVGLSYSYVDATYRSSWIEHSPA---NSTA-DANGNVTVKPGDRIPGIPAHTVKLRLD----YAAT-PAWDIGANVTWRGGVYAR-GD--ENNGDVNGR
I35_RS20820(BCAM0948)  505 AGVSWRPVA---------PLRVETSLAYAWGRN------VAS------------GDPLPQMPPLEARIGLEYTRGA------WSAGGLWRIVASQHRYALNEGNVVGKDFGP
I35_RS23575(BCAM1571)  515 LDGKWRAFSRRGH-----TVDLELTADYTHARN------VDT------------GQPLPRIAPLRATLAADYGYGP------FGARAQVTHAWSQHRVPD-------DDFS-
I35_RS18860(BCAM0564)  554 LELKAQPVN---------SLYLFANLGMLNTRY------TEFRNVPT-----AVGNSFARSPHTTLNAGVDYRVPVSF--GTLTAGGDVNYRSREYFSAT-RQTMP---QLW
I35_RS11045(BCAL2281)  541 TAVYGEPWK---------GVRLIAGATYLDATL------QNTAG------GTNDGHRPIGVPSFLLNAGAEYDVPML---RGLTLTARWIHTGPQYL---DVANTM-----S
I35_RS00620(BCAL0116)  531 VGARAKFGA---------HWNAG--VDAMLLDA------WYANG------IGNHGNRVAGAPRFVLAGDLGYAVPGV---PGLTLGVDAKETGATPL---RAAGGL-----D
I35_RS19580(BCAM0706)  547 LGAAGRVTE---------RLGLTASVAAIRARA------VDSGS------PAYEGHQIINVPALRASLYADYAVPGV---AGLNVLGGVEYSAARNA---NEEGTA-----R
I35_RS26975(BCAM2224)  531 FEANGRITP---------WWSVWASYTYDTMRY------ADNLANAGS-------FAPLLNPRHLFRLWTNYDLP--WQERRWSIGGGVQVQSSYSA----QANGV---TMS
I35_RS06170(BCAL1345)  532 LTGGARLPH---------GWTIDANVALLRARY------DAFNQTVGGATVSRAGNVPSGVPQQTANLWVGWAFAERWQ-----ANAGVRYVGATYG---DDANRV-----Q
I35_RS18505(BCAM0491)  530 ATVGAEIAK---------DWRVDANVSILRAKY------DDFQQSSGGTTVSRAGNVPVSVPQRLANLWISWRFAPDWT-----GIAGVKYVGKRFA---DTANQL-----V
I35_RS06295(BCAL1371)  541 LDVSGKIGE---------RVNVIASYAYIDAKT------TED-PLYAG-------NQLWNVARHTASFAAVYDFGTVAAGDDLRLGADVRYVGARPG---DSANSF-----T
I35_RS28095(BCAM2439)  531 LDVAGQITR---------HLSVIGSYAYTNAND------RDS-----N-------TPLVNVARHTGSLFAVYDTAIANLPGRWRFGGGARLVGARSG---DTANSF-----T
I35_RS08065(BCAL1700)  544 LSATGKVTP---------NLSVVASYVYQDVKV------IQANDVSLN-----NWPVDIPRPRQMASLWTDWTWHTGPL-AGFGLGGGIRYQSASAG---AADNSL-----T
I35_RS31745(BCAS0333)  543 LSAVGKVTR---------ELSIVASYLYQDVKN------VQANDASLN-----HWPVSVPLPRQMASMWADWTWHTGAL-AGVGVGGGVRYQSASAG---APDNSL-----T
I35_RS25625(BCAM2007)  569 LGLAGQITK---------QWQVFGGYTYMKSEL------RDNGKDTA-----NNGNRFPNTPKHSLTMWSNYDVTPKFT-----VGGGAFYMSEVFGD---PANLR-----A
I35_RS21645(BCAM1187)  556 FGFAGNLTQ---------KWHVFGGYSYLNAIT------TDAGPGSPG----ASGLPMVMVPKHNFTLWTSYDVMPKLT-----LGAGATVMSKTYASV-SPTVKK-----W
I35_RS31880(BCAS0360)  530 FEWSGDLGH---------GLRGLANLAYVDAEV------TRDAVLTPG-------SRLVDVPRLSGSALLMYETTLPFA-DKAGAGAGVIYVGRRAGNTANTQDGF-----D
I35_RS18545(BCAM0499)  534 LGFTGEIAP---------KWSVYAGYAYLNGTV------DGSAQSTAAG-LAVSSNTPGLMPRHSANLWLKRELPYGFY-----AAAGMQFQSARYT---SASDLV-----T
I35_RS08115(BCAL1709)  541 LGVAGRLTG---------DWFVRGGIGWQHARV------VDAEPKYAG-------KRSAGVSASNGSLFVSHAPLRGFF-----AELGVVYEGARYA---DRDNLL-----E

I35_RS08460(BCAL1777)  772 VASYSQFNLMFNYRG---FKHWTIYGGITNLFDKK--------PPFDVEWQAVPDITGYDQSLYTNLGRFFQVGAT--YRF-
I35_RS08490(BCAL1783)  782 VASYSQFNLMVSYTG---FKHWTIYAGIDNIFNRA--------PPFDPVYMNASYQTGYDTSLYTYVGRFAQVGAT--YKF-
I35_RS29035(BCAM2626)  636 PPSSFVVDLRGGYRF---NKHVSATIGIRNLFDRKYWNWSDVRGIAADSQVLDA---------YSSPGRTVAVSMK--VDF-
I35_RS04375(BCAL3001)  668 TSSFTVVDLKARYRF---DRHWTASAGIDNLTDRRYYTFHP-----------------------Y---PGRTFYGELK--WSL-
I35_RS23700(BCAM1593)  550 LASYLTVSLRASYRI---NSHLTVSASLSNLFDRQYMTAYG-----------------------YNTLGRTAFGKVS--YTF-
I35_RS27690(BCAM2367)  639 LAGYVLVDLDMRYRI---TKRFEVFASVTNLLDKRYASFGALGQNFFNGPNHTFDGARPVNEQFVGPGAPRGAWVGVRYAWD
I35_RS20820(BCAM0948)  584 SAGFGVLSLHTQYNV---SKTVQISVGVDNVLNKAYTEHLNLAGNAGFGY--------PANAPVMEPGRTAWVRVS--AKL-
I35_RS23575(BCAM1571)  590 TDGYTSLGVMLTYKFRVGPTHWLAYLRGDNITNQEIRYSTS-------------------VVRGFAPQGGRSVMAGLRTTF-
I35_RS18860(BCAM0564)  640 QGGYTVLNAHVSYTT--PNQKYIVTGYVTNLTNKVYKK---------------LELLPSYGAYPVLYGDPRTVGITLTAKI-
I35_RS11045(BCAL2281)  621 IQAWDRFDLGARYATDVFGKKTTFRATVRNVANKSYWSS--------------------TIGGYLTQGDPRSVWLSMTTDF-
I35_RS00620(BCAL0116)  609 APGFLVVNAGARYLTRVGRHDVTLRASIDNVLNRRYWEY--------------------QYADYVKPGDPRTVSLSAKIDF-
I35_RS19580(BCAM0706)  627 VPSWFVFNLGARYTTKIGGHRTVLRVSVDNLFNKFYWRDAGEQQ----------------GDAYLFPGAPRTARVSLTYDF-
I35_RS26975(BCAM2224)  612 QGGYALASVRLGYRY---DKHWSAALNVNNLFDRHYYLSLSQP-----------------GWNNRYGEPRNVMLTVRGQF-
I35_RS06170(BCAL1345)  616 VPSYTVFDASLRWQP---TSRTELALYLRNLANRTYAVTTSNG-----------------GEQWLLGPSRSAELVATMRF-
I35_RS18505(BCAM0491)  614 MPSYTTVDLGLAWKP---RKDTTITARAYNVFNRRYVQSAYYN-----------------ETQYLLGNDRRFELLANYRF-
I35_RS06295(BCAL1371)  622 LPSYVLADAFATYDTRIGKQKLSFQLNVKNLFNRTYYPSSAN------------------RYFVAVGDARQVSLLTTLQF-
I35_RS28095(BCAM2439)  608 LPGYVSVDAFAAYETTIGKFPTRFQLNVKNLLDKTYYPSSNN------------------NLIVAVGEPRLVTLTTTVSF-
I35_RS08065(BCAL1700)  627 VSSVTLFDAGVHY-D---TRNWRFAVNGTNLFNRHYISGCQS------------------MNVCIFGTDRTVIAIAKYNW-
I35_RS31745(BCAS0333)  626 VPSATLYDLAIHY-D---LPRWRFAVNVANLFDRRYVSGCQS------------------YAVCVFGNERTVLASAKYSW-
I35_RS25625(BCAM2007)  648 VPSYWRFDAMAQYRI---NKKLDLQLNVNNLFNRTYFDQAYPA-----------------HYASIAPGRSAFVTLN--ARY-
I35_RS21645(BCAM1187)  638 TPGYARFDAAATWRV---NKTMDVQLNVQNLFDKQYYASAYPI-----------------YATWAPGRSAMVTLN--FYQ-
I35_RS31880(BCAS0360)  614 LPAYATVQLNGYLQV---NKHLRASVVLNNLFNRTTYVSSY-------------------NSLWVTPGAPRSLFASLAYSF-
I35_RS18545(BCAM0499)  617 LPSFTVFNLGAGY-R---SKKVDVTLTLDNLFDRRYFIAAHGN-----------------ADLYNMPGDPRTLTATVKWHM-
I35_RS08115(BCAL1709)  618 LPAYLRWDGKAGY-R---TRDLEVTLAAVNLADRDYYANATGL-----------------AQIVPGAPRTFTLTAAYKF-
```

Fig. S3. Amino acid sequence alignment of *B. cenocepacia* TBDTs. Amino acid sequences of putative TBDTs from *B. cenocepacia* H111 were aligned by Clustal Omega and identical or similar amino acids were highlighted using BOXSHADE. White font with black shading indicates identical residue at the corresponding position in ~50% of sequences and white font with grey shading indicates similar residues at the corresponding position in ~50% of sequences. Red box indicates the location of the TonB box conserved region (21). Orange box indicates the location of the receptor plug domain. The names of the corresponding TBDTs from J2315 are shown in parentheses, with the two non-functional J2315 TBDTs shown in red font.

H111ΔpobAΔorbA

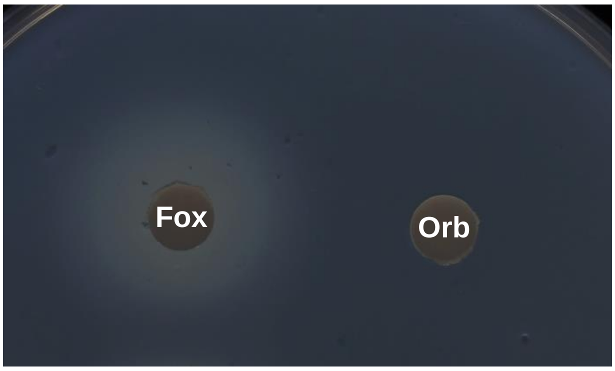

H111ΔpobA

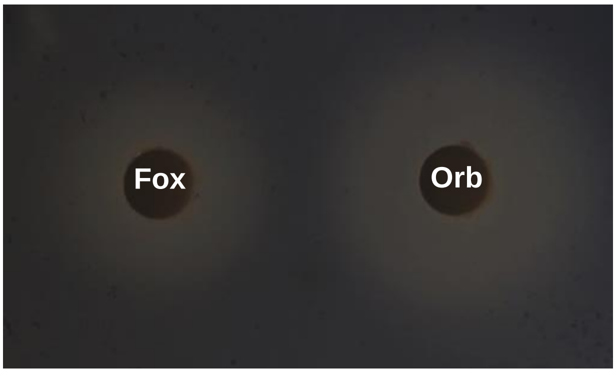

Fig. S4. Effect of inactivation of *orbA* on hydroxamate siderophore utilisation. A disc diffusion assay shows that whereas a *B. cenocepacia orbA* mutant is unable to utilise ornibactin (Orb) it is able to utilise ferrioxamine B (Fox).

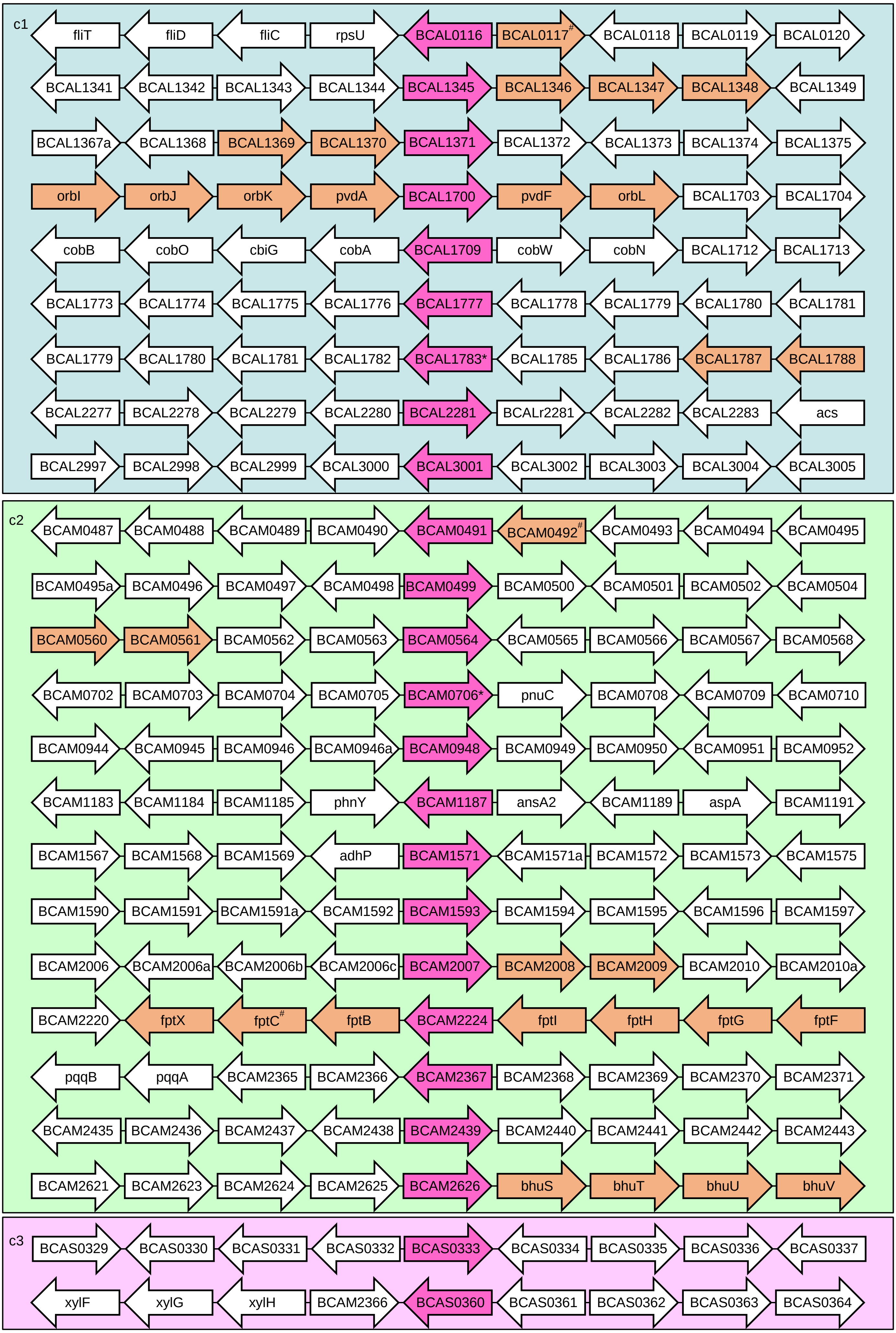
c1
fliT fliD fliC rpsU BCAL0116 BCAL0117# BCAL0118 BCAL0119 BCAL0120
BCAL1341 BCAL1342 BCAL1343 BCAL1344 BCAL1345 BCAL1346 BCAL1347 BCAL1348 BCAL1349
BCAL1367a BCAL1368 BCAL1369 BCAL1370 BCAL1371 BCAL1372 BCAL1373 BCAL1374 BCAL1375
orbI orbJ orbK pvdA BCAL1700 pvdF orbL BCAL1703 BCAL1704
cobB cobO cbiG cobA BCAL1709 cobW cobN BCAL1712 BCAL1713
BCAL1773 BCAL1774 BCAL1775 BCAL1776 BCAL1777 BCAL1778 BCAL1779 BCAL1780 BCAL1781
BCAL1779 BCAL1780 BCAL1781 BCAL1782 BCAL1783* BCAL1785 BCAL1786 BCAL1787 BCAL1788
BCAL2277 BCAL2278 BCAL2279 BCAL2280 BCAL2281 BCALr2281 BCAL2282 BCAL2283 acs
BCAL2997 BCAL2998 BCAL2999 BCAL3000 BCAL3001 BCAL3002 BCAL3003 BCAL3004 BCAL3005
c2
BCAM0487 BCAM0488 BCAM0489 BCAM0490 BCAM0491 BCAM0492# BCAM0493 BCAM0494 BCAM0495
BCAM0495a BCAM0496 BCAM0497 BCAM0498 BCAM0499 BCAM0500 BCAM0501 BCAM0502 BCAM0504
BCAM0560 BCAM0561 BCAM0562 BCAM0563 BCAM0564 BCAM0565 BCAM0566 BCAM0567 BCAM0568
BCAM0702 BCAM0703 BCAM0704 BCAM0705 BCAM0706* pnuC BCAM0708 BCAM0709 BCAM0710
BCAM0944 BCAM0945 BCAM0946 BCAM0946a BCAM0948 BCAM0949 BCAM0950 BCAM0951 BCAM0952
BCAM1183 BCAM1184 BCAM1185 phnY BCAM1187 ansA2 BCAM1189 aspA BCAM1191
BCAM1567 BCAM1568 BCAM1569 adhP BCAM1571 BCAM1571a BCAM1572 BCAM1573 BCAM1575
BCAM1590 BCAM1591 BCAM1591a BCAM1592 BCAM1593 BCAM1594 BCAM1595 BCAM1596 BCAM1597
BCAM2006 BCAM2006a BCAM2006b BCAM2006c BCAM2007 BCAM2008 BCAM2009 BCAM2010 BCAM2010a
BCAM2220 fptX fptC# fptB BCAM2224 fptI fptH fptG fptF
pqqB pqqA BCAM2365 BCAM2366 BCAM2367 BCAM2368 BCAM2369 BCAM2370 BCAM2371
BCAM2435 BCAM2436 BCAM2437 BCAM2438 BCAM2439 BCAM2440 BCAM2441 BCAM2442 BCAM2443
BCAM2621 BCAM2623 BCAM2624 BCAM2625 BCAM2626 bhuS bhuT bhuU bhuV
c3
BCAS0329 BCAS0330 BCAS0331 BCAS0332 BCAS0333 BCAS0334 BCAS0335 BCAS0336 BCAS0337
xylF xylG xylH BCAM2366 BCAS0360 BCAS0361 BCAS0362 BCAS0363 BCAS0364

Fig. S5. Genomic context of *B. cenocepacia* TBDT genes. Chromosome 1 (c1), pale blue background; chromosome 2 (c2), pale green background; chromosome 3 (c3), pink background. Genes encoding TBDTs are shown in magenta, whereas other genes that are potentially involved in iron homeostasis are shown in orange. BCAL1367a, BCAM1571a, BCAM2006c and BCAM2010a were not recognised in the original annotation of the J2315 genome sequence and have been provisionally given these names here. BCAM2006a, BCAM2006b and BCAM2621 are pseudogenes. All genes are rendered as the same size and so the figure is not to scale.

*BCAL1783 contains an in-frame stop codon and BCAM0706 contains a frameshift mutation in J2315 but are both wild-type in H111.

#PiuB/PepSY domain-containing proteins.

(a)

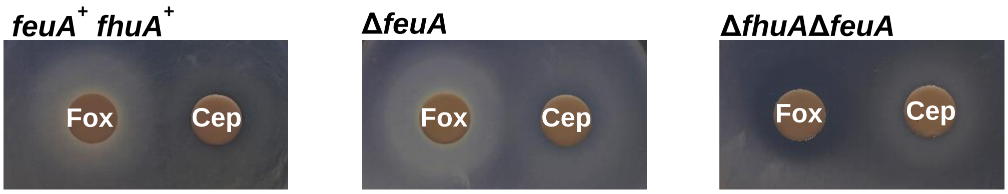

(b)

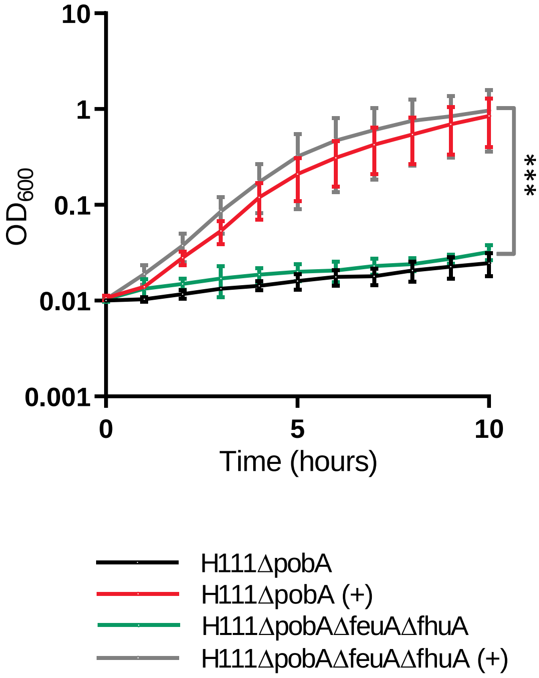

Fig. S6. Effect of cepabactin on growth of a *B. cenocepacia* Δ*feuA* Δ*fhuA* mutant in iron-limited medium. (a) Disc diffusion assay comparing the effect of cepabactin (Cep) and ferrioxamine B (Fox) on growth of the H111ΔpobAΔ*fhuA*Δ*feuA* mutant. (b) Growth of the H111ΔpobAΔ*fhuA*Δ*feuA* mutant in M9-CAA liquid medium in the presence (+) and absence of 30 µM cepabactin. Data were derived from three independent experiments (n=3). Error bars represent the ±SD. *** = P < 0.001.

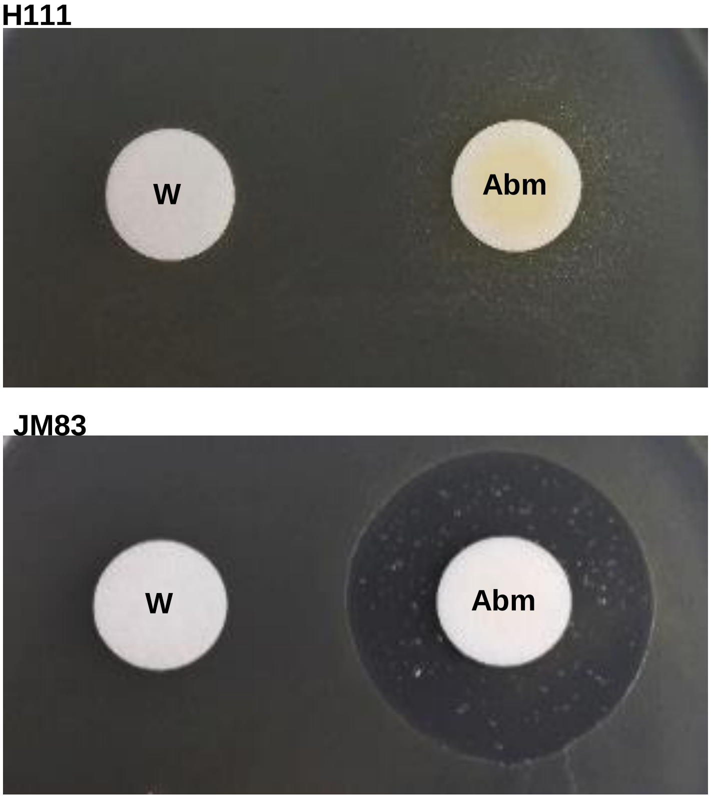

Fig. S7. Effect of albomycin on growth of *B. cenocepacia*. Filter disc loaded with albomycin (Abm) (5mM, 10 µl) and its solvent, dH_2O (W), were placed on M9–CAA agar plates seeded with (i) *B. cenocepacia* H111, (ii) *E. coli* JM83.

```
                                                                              PepSY_TM1
BCAL0117      -------------------------------------------MNAFLRPFLVRLHRWFGLAIALFLFVAGLTGALIAWDHELDAVLNPDFYTARSGA---A      56
BCAM0492      MSTTV------------------------------DRAVAPARSASAGYRTLWRWHFYAGLFVMPFLVILAITGTLYGFQPQIEPLLYPHRLVVEP-R---A      68
BCAM2222      -------------------------------------------MDASSRSRWRALHRGAGALFGVVLFVVLFRGAWSLAFDSMQGWWRPPAVAVARPT----      55
RSp1642       -------------------------------------------MKSATLRLYQTLHTWVGLMAGWALFIAFFAGSITVFHDELHAWQDPRRSHSAAAV----      55
NMB1721       ---MD----------------------------TQIK-TEADNQSNRRYLTVWRWHFYAGLLVMPFLTLLAVTGLGMLLFANITGK-EGERIHVVP-Q----      64
PA0801        ---------------------------------------------MNLRQSMSGLHTWSGLLVSWLLFVIVFAGSLASFDKELTRWMQPDLHLPGAQS----      53
PA1909        -------------------------------------------MTAKTLRTWYLVHKWTSLISTIFLLMLCLTGLPLIFHEEIEHYFEPHPQLEPLTA---E      56
PA2403        ------------------------------------------MSKKSRSRLWFLVHSWLALPIWFFVLIVCVTGTLAVVSQEIVWLANPDVRASKPYE---D      57
PA2465        -----------------------------------------------MRPVLVLLHRYVGLATALFLFLAGLTGSLLAFHHEIDEWLNPGFYAVGEGG---E      52
PA3789        -------------------------------------MQSNEKNGRDFYALAWRWHFYAGLFVAPFMILLALTGIVYLFKPQLDALLYPQLLRVEA-----G      60
PA4219        ---------------------------------------------MAARPLLLSLHGGAGALFGVLLFVVLFSGAWSLGHDDLREWLRAPAQAG-GEA----      52
PA4513        -----------------------------------------------MKKVWFQLHWFFGISAGLVLALMGVTGATLSFQDELMRALNPDVLVVQK-R---P      51
VCA0891       -MSTV----------------------------TTPQ-PTAAARRKTLYFLTWRWHFYAGLFVVPFMLMLALTGLVMLFDDEIEQARYAEVLNVTP-Q----      67
VPA1524       MLRNS----------------------------TKPQ-LKEASRAKSIYFMTWRWHFYAGLFVIPFMLMLSVTGLVMLFDDEIELARYETTLKVVQ-Q----      68
AGR_C_3630    MSVTTS---------------------------FEGERAPAQSSSINLYRAVWRWHFYAGLLVLPFMISLAVTGALYLFRDEFDNLIHSDLKRVEVVQ---N      72
CC_1782       ---------------------------------------MASRSKPSVRAFARQVHLWLGLSIGVLFAVVGLTGSILVFYTAIDDALHPEIRLRGAAP----      59
CC_2927       -------------------------------------------MKARTLRAWSWVHKWSSLVSTAFLLMLCITGLPLVFSHEIEHVLMEE-AWTPARP---D      55
CC_3501       ---MTA---------------------------IDNSETP--APAGAVYRAFWRWHFYAGLLVLPILMLMALTGGLYLFKDELTAVVHRPLVVVA--E---G      65
PP_0860       -----------------------------------------------MKKTLFQLHWFFGITAGLVLALMGITGALYSFQEELLRAFNADVLKVEV-R---A      51
PP_4610       -------------------------------------------MKEGFRQAMAWLHTWTGLIFGWLLFAIFLTGTLSYFKEEITHWSQPEVRSH--------      51
PP_4839       ----------------------------------------MRGTRVSFYNLAWRWHFYAGLFVAPFMILLAITGIIYLFKPQLDPLLYRDLMVVEA-----G      57
SO_3407       -------------------------------------------MKETFFRTLSWLHTWAGLLVCWVLLLIFFAGSLSYFRHEISLWAEPELHRGAFQD----      55
XAC4036       ------------------------MQIGCETHSHLQWPAVLFRGQVLIKTLLFQLHWLLGITAGLVLSVMGLTGAAMSFENEIVRMANPAIAQLAQ-RHAAG      77
BH1854        ----------------------------------------MNETKPSLYQVIWRWHFYAGVIVAPFLIILAVTGGIYLFKPQIEQTLYQQYYEVTP-E---G      58
BH0982        MVEAAN---------------------------ERVKTKEAEQGKRARYQVVWRWHFYAGLIFSPFLIILAISGGVYLFKPQIEASLYQELYYVQE-T---K      71
SCO0863       MSESPPLPDTPVTPPSSAAAEVVPLRSGDVTKPVTATGAGSGTTRASLRPLLLRLHFYAGVVVGPFLLVAAVTGLAYTAAPQIESVLYEHELKVT--P---R      97
SAVERM_7350   MTTAPSAPDAPSTTTD----------------EPPQSGTPGPARWAPLRPLVLRLHFYAGLLVAPFLLVAALTGFLYAASYQAEKIVYAHELTVP--V---G      81
BC_4751       -----------------------------------------MKVNRSLHYIFWRWHFYAGLFITPLLITLSLSGIGYLFREEVEDFIYKDLYFGKSAQ----      57
Cgl2326       MRSVLDYWIMTLAPSTNAV---------------SQQ---TRRNPSQAPRLLHRLHFFAGIICAPLIFIAALTGLVYAFSPTIESISNQEMLTVSKSA---S      81
bll7967       ---------------------------------------MAARLGHNIKAVLRQVHSIAGLALALLLSLIALTGAIISFEDEIVDHLNAGIMQVLP-R---S      59
NE0550        MSSESA-----------------------HQHAAGVDEERGTLPRRSWRQLWLSIHLYLGLFIGALLVILGLTGSIAVFWAEIDEWLNPELLTVTVPE---Q      76
NE1084        MKSN--------------------------ADSLTDTATESDPPVRGFRQRMTWLHTWGGLWAGWVLFAIFLTGTLGVFDDAITRWMKPERPLVAEVA----      72
NE1984        ------------------------------------------MIRADIIKLYKTVHTWTGITCGFALFIAFYAGALTMFQEPIARWASPPAVGVA-AV----      55
BB3604        ----------------------------------MKPARTKAAGEEGLRQSMSWLHTWSGLIFGWVLFAMFLTGTLAFFRPEITRWMQPEIDVA--------      60
BB4741        --------------------------------------MRPDGKTEGLRQAMSWLHTWSGLLLGWLLFAIFLLGTLSYVRDEISVWMKPEVHGS--------      56
WS1409        -----------------------------------------------MRRFFLWIHKWLGLLSTIFLVIAALSGSIIAFTDEIDRWLNPKLFSIDSSK---E      52
                                                                     *                **
```

```
                                                                                                                    hDhXXG
BCAL0117      --PLAPL--ELAAR-----I--EAADP--RVQ----VT-YLPLA-IEPGHTL----QAGVMPRTDPATGQPYALGFSQIAVDPATGAVQGRREWGAPSLARL    135
BCAM0492      TPRLDAD--------TLIARA-RTAMP--ADA----TPVSVQVA-TAPDRSAE----YVFR-LR--------DGSRESVYVSPYDGRVIGTLSVEN-R----    136
BCAM2222      -LP---------------LDAL-VTRAASLGVA--LRDA-RIVLP-RPDDPAIR----FCDAR------------QTCTLALDPATGAPLP----------DA    111
RSp1642       -PVD------AAAVDRFISAL-VRAHPS--AA--ADAY-V-NLP-TKNEPGLT----AYWFEQGAWRAT----------TDAKLAAGRTAPEDTALEPTR--G    127
NMB1721       ATVQPLS--------VQAEAA-RSAVNPETSS----VV-QYIAP-RADDMVAV----FRVN--N--------EGKATMVAVDPYTAKVVSTMPRNQ-G----    132
PA0801        -LG----------ADQVRDWL-RQRAP----D--AHAW-WMLPP-SERAPYWN----AGWEPRD------GSEFKV--FQLDAVSGQPLPK----------T    113
PA1909        SPRID-----YDDV---IARA-LAARP--GEV----VR-FLVFE-KDDPLGLVISAPSLVPPPENG--H---------MQPFDARTGEFFD----ALPPPG--    125
PA2403        AEPLS-----FSQV---LKAI-NEAQP--DLL----VE-SIQRP-DEEHFA--LTAEVSYPGGSEAT------------LYINPYIGAIQG----ESPSF---    122
PA2465        --RLSPG--SLVQR-----V--ESRYP--RQL----VW-YMEYP-EAGGHPA----LLATVPREAGAK-----VEHDVFYLDPVSGEEVGKRLWAACCFQPA    126
PA3789        AQMRSAD--------QLLVGV-RQAHP--QAA----VS-QYLPP-SEAGRSAQ----FVI--GE--------EGRQWNLFVDPYSGREIGRQDAQL-N----    126
PA4219        -LA---------------LERL-LERAGEEGVD--IRDA-TLLLP-APGHAAFS----VCDAR------------LDCRLDLDPASGRVIP--PMP-------    109
PA4513        EGVLPLD--------ELVRRI-EGAEP--GRK----VA-FVWVE-MDSDKAGR-I--FYTPPPG--------QRRGESRWIDPYTGAFVGEPRGE--G----    119
VCA0891       AQVMPVS--------QQLAAV-QKAYP--EAQ----VT-QFIPA-HQPDLANR----FSVL-FS--------DGSTQFVTVSPYSAVVIGAIDRSE-S----    134
VPA1524       EHKVPVS--------VQLESV-KQAYP--DFS----VT-QFVPA-KTAHLANR----FSIK-AE--------DGRSLVAAVNPYTGEVQGTIDRSD-S----    135
AGR_C_3630    APKALPS--------EIIAAA-LAAVP----G----TAVKYTTS-ADPGASTE----ITVD--T--------EDGKRAVYVNPYTAEVAGSLPDRG-T----    137
CC_1782       -------PPSWEQV---YQTARA-TYP--DKA---GPW-RFEVT-EAGGDIP---ARYYKPPERA--------DRDFAPMMVWFSADGGRVI---RRDYWGE    129
CC_2927       GPKLN-----LDQV---LDTA-LARKP--GEV----PA-YMSFD-EDRPVVNVTSV---QPGGKAGVYS--------FQPIDQTSGD--------PAPPVAGH    122
CC_3501       PARTAPT--------AWIKAA-ETATG----G----AAKQVVIP-ERADRSVL----VTVE--A--------KGEKQTAYVNPYTGAVIGTTQ-PG-G----    129
PP_0860       EGVLPPA--------ELVKRV-EAQQ---QDK----VS-MLWVD-VREGNAAR-V--FFMPAPG--------ERRGELRYADPYTGELKGEAAGQ--G----    118
PP_4610       ----ALD--PINSLAVAQRYL-QDNAG----H--SSTW-FIRMP-NEREAALS----VGYR--DPS--GGPRGFVRK--TLDTQTGQPV------E--ARDS    120
PP_4839       QHRQGAD--------TMIAEV-RQAYP--KGH----IA-QYLPP-LNAARSAQ----FVV--HD--------GGRELNVFVDPYSGKVIGEQDGKQ-N----    123
SO_3407       -YQ-ANQ--VVSQLDKGQDYI-ENHSP---AE--AQRW-LINFP-TQRNPMLS----FAWQLPPEK--GQRRGK------IEQHTALADSSDM--ITHVRDS    131
XAC4036       ERALPVD--------VLLQRL-DLAPA--GAGQKQAVT-RLLID-PTGA---R-P--SAA-RLS--------GKGAGRVYFDPYTGERVAPPRLS--T----    145
BH1854        -DRLSPD--------EQLEMV-HEQYP--DSV----IT-AYRPG-ESSTRSSE----VTL--ST--------VNGPLTVFLDPYTGEQLGTLYSED-R----    123
BH0982        EATLTPS--------ALVARA-KEAFP--ESP----VS-SVRFY-DDPQRTTE----ISM--MK--------DGERISVYVNPYNGEVQGSLVNSE-K----    137
SCO0863       GSAEPLA--------EQVAAA-ERAVP--DGT----LL-SVTKG-AGPTDSTR-V-TFGKDGLA--------EGYTLTAFVDPYDHEVIGTLETFG-Q--WL    169
SAVERM_7350   DTELPIS--------QQVAAA-RKAHP--EGT----LT-AVRPS-PEADATTR-VLLSGVKGVD--------EGHTLAVFVDPYTGKVRGSLEQYG-STGAL    156
BC_4751       TESISMS--------DSISLT-EKKYP--HYS----VA--KISE-FNGDYNTR----LTIANEY--------TGQQKYVYLDS-NNQIVGDQNASE-T----    123
Cgl2326       DTALPVR--------EQVSIA-QELHP--DLD----LS-GVRLG-DDS--STT-RVLFADETLA--------ESTVRAVFVDPYTGEITGDTTQYG-SSAAL    154
bll7967       TPALMPD--------ELVARL-KAAQD--FGK----VS-AVTLS-SDPAAAVH-V--RFGRDEQ--------GGRPASLYLDPYDARVIGSPRGE--E----    127
NE0550        K-NLAPGAPAYQSLDEIIRVARQAAAP--DSR---ITT-VYGAR-N-SEAVY-------------AVYASQPSSAWQRIFVDPYRAQVTGVRSYGANEWIPN    156
NE1084        -PGSAEQ--RAQAVRLAQTYL-QQAMP----R--GEFW-SIGLP-GESDPAIR----LFWRENE------DAKFQQTR--LDPVIGTELD-----KAVDRET    145
NE1984        -PLD------DA--PRLLELV-AAAHPE--AR--EEGI-KLHLRGYENEPA-R----VTWEEDESHEAGQPHGHVHWWATLKPD-GSLLA------KQEEPS    130
BB3604        ----PVS--AEQASAVAQRYL-QAHAP----D--ARRW-FVTLP-TQRAPAIQ----LSYPAAKPK--PGERGFTR--VSLDPATGDALH--------ARQT    131
BB4741        ----QPD--GQT-AARAVAAL-ATMAP----D--ALSW-TVALP-TARDPAAH----VSWRDPGAA--AGKAGLRR--AVLDAGSGQAIQ--------VRET    126
WS1409        GVALKSS--------EWIAKV-KERFP--QAH----IN-SYHLP-QKSSDSI----LAGVKFGKGA---PEPWSDYNQLFIDPRDGEVIGGRNTMRCCSQ-E    129
```

```
                                          PepSY_TM2                                               PepSY_TM3
BCAL0117        DLMPFIYRLHYSLFLPVYGGINFGFWVMGVVGIVWAIDSLIALVLAFPNL--KS----WRKSFAFRVRRGGYPLVFDLHRSGGVWVWGLLLVVAITSISMNL   231
BCAM0492        -FMQVDRMLHRKLLLG-----KTGEWLMELAACWTFVMIGTGIALWWPRGAA-----RVGRALRPDLSLRGRPLWKSLHATAGIWLAAGTVAFILTGLPWSG   227
BCAM2222        GRATLIVTLHKTLFAG-----FPGRIFVSLWGIVLLVLIVAGIVVHR-KR--------WPDAARIRRGHGLRVALFDLHAWIGLWGTPWLVLFAFTGALSGL   199
RSp1642         ELANFINNLHYALGFP-----DVGIYFMGAVSVLYGLALVSGVLLHLPRL--------KKDLLAVRHGRNLKRFWMDVHNVLGLFSLPFHLIFAMTAPLLCL   216
NMB1721         -WYYTMDEIHSDMMLG-----AAGDYLLETAASLTIIMVVSGLYLWWVKRRG-----IKAMLLP--SKGRARSWWRNLHGTFGTWVSLILLLFCLSGIAWAG   221
PA0801          VGGEFFFTLHYDLHAG-----MVGLYIVGIAGMLMLVALVSGTIVHR-RI--------FKDFFTLRPQAARQRAWLDAHNVLGVIGLPFHLMIAYTGLVIFI   201
PA1909          GFMYLMLKLHTDLFLG-----LPGYLFLGLMGLLLVASLVSGVVVYTP------FMRKLDFATV-RAERSRRLKWLDLHNLLGIVTLSWVLVVGVTGVINTL   215
PA2403          DFRQFTRALHGWWLVPFNNGYSWGWYMVSLLGLPMLASLITGLVVYKR------FWKGFLKPRL-RINQGARIFWGDFHRLSGIWSIWFIALISITGTWFLI   217
PA2465          NLVPWVLEFHHNLTLP----GNWGLYLMGGVAMFWFLDCFVGAWLTLPRG--RPFWSKWTTAWKI-KRGNAYRFNFDLHRAGGLWLWLLLAPVALSSVALNL   221
PA3789          -LQAVARALHGELMVG-----TVGDRLIELAAGWGIVLVVSGLYLWWPRGRA------ASALFWPRLHLRGRPLWRELHVLAGFWGSLLLLFMLLSGMTWTG   216
PA4219          -ALDLLLNLHKSLFVG-----FPGRVLVSLFGVSLLLLCLAGVLLHS-RR--------WRDLRRWRRDRGLRLALFDLHGLIGIWGLPWLLLFGFTGALSGL   196
PA4513          -FFNLMMQLHRFLAMG-----EYGKQVTAACTLILIFFCLSGLYLRWPRKVF-----DWRAWLTLDWSRKGRSFNWDLHAVAGTWCLALYLLAALTGLYWSY   210
VCA0891         -WYEWANSIHGTLLIG-----DWGDYLIEVAASLGMILLVSGIYLWLPLDNA-----RKAGFLKIRVGSGARIFWRDLHANLGGMLSLVLLFFLISGLSWAG   225
VPA1524         -VYELMNNIHGTLLIG-----EFGDRLIEISASLGILLLVSGLYLWLPRDNA-----SRAGFLKIRIAQGSRILLRDVHANLGGVLSLVLLFFLISGLSWAG   226
AGR_C_3630      -IMWTIRYLHSLKYFG-----TYARYLIEIAAGWSILLVATGIYLWWPRKQT-------GGVVTVRGTPKKRVFWRDTHAVTGIFVGFFIVFLAVTGMPWSG   226
CC_1782         YAMTWVYDLHHRLLLQ-----ATGGAILGWSGLAMLALMVAGLTAWWPRR------GGWRKALQMKPRAAPLRKLYDWHKQTGLWSFGLLVLLTLTGVLLAL   220
CC_2927         PVMEFLLQLHTDMFLG-----LAGMLFLGAMGLLLIVALVSGVVLYAP------FMRRLPFGTV-RASKRARTRWLDYHNLLGAVTAAWVLVVGATGVVNAL   212
CC_3501         -VMGVIKHLHSLEIAG-----PVMNLLVEVVAGWTIVLVATGIFLWWPRGQK-------GGVVTVRSQPAKRLFWRDLHAVTGIFAGGVIAFLAVTGMPWSA   218
PP_0860         -FFNLMLKLHRFLAMG-----DTGRQITGACTLMLVFFCLSGLYLRWPRKAL-----DWRTWLTFDWAKKGRAFNWDLHAVAGTWCLLFYLLFALTGLFWSY   209
PP_4610         RGGEFFYRFHFQLQMP----YPVGRWLSTFCAFIMLLGLVTGIITHK-KI--------FKEFFTFRPGK-GQRSWLDGHNAIGVLVLPFHLMISYSSLVLFM   208
PP_4839         -LQAIARALHGELMVG-----TVGDRLVELAAGWGIVLVVSGLYLWWPRGRS------GAGVLWPRLSARGRLFWRDLHAVTGFWGSALLLLMLVSGMTWTG   213
SO_3407         RGGDFFYRLHFDLHYM---PAITARYIVGVCTMFMLIALISGIVIHK-RI--------FKDLFSFRQNK-GARSWLDAHNVSSVIALPYHLMITYTGLITLM   220
XAC4036         -AFAFIEDLHRNLTAG-----KRGQAVTGAGALILLFFCASGLYLRWPRRWW-----SPRTWWVVEWRRQGRSFLWSLHAVFGTWCLLVYLLVALTGLTWSY   236
BH1854          -IMNKIEEMHGELMAG-----TIGDRIVELAACWALVLIITGLYLWFPQKKK-----SFAGVLYPRWKKGKRAFRRDLHAVPAFWIGAGMFFLIITGLPWSG   214
BH0982          -LTEIFKKIHSELWVG----GTFANRLVELAACWAVILLLTGLYIWWPRSRT-----SILGTILPRFKKKGRVFWRDMHAVPAFWLSLFIFILIATGLPWTG   229
SCO0863         PARAWLDDLHRNLHLG-----EFGRNYSELAASWLWVEVLGGLALWAGTPRN---RQRLRRLMVPDGGAKGRRRTMSWHGAVGLWASVGLLGLSATGLTWSG   263
SAVERM_7350     PLRTWIDEFHRDLQLG-----ENGRLYSELAASWLWVIAGGGLVLWFGRRRA---QRKV-------RGTSGRRRTLGLHGTVGVWAAAGFFFLSATGLTWST   243
BC_4751         -FANIMRELHSSLLVG----GTVVNYTVELAACWTIFLIVTGLYMSIRQFKN-------------TPSSNKREKAKRRHSIIGIIFTIPLFLLVASGLPWSG   207
Cgl2326         PFRQWVSQGHRMLWLG-----EPGRIYSELAASWLGVLAVGGFALLWLRNKK---PGRLRKMV--RTGGRGRVKTYRRHAALGTVAGLGFVFLTFTGLTWST   246
bll7967         -FFATVRRLHRWLLIP-GDAKGWGRQVTGVAALGLIVMLVSGLVLRWPRRAG-----SVKMWLKPNLGLSGRGLHRTLHAVIGTWVLLIYLVMTLTGLWYSF   222
NE0550          YFMDVIFQLHFSLLLG-----MNGQTLMAVCALLLLVSLITGLIVWWPTS------GQWRKALTIKRGAGPVRFNFDLHKTLSLYLFPVLGAVLLSGVFMNL   247
NE1084          EGGHHFVHMHFEFHAG-----EAGIWMVGFFAMIMLVALVSGVITHK-RI--------FKDFFTFRPKK-GQRSWLDAHNVASVLTLPFQFMIVYTGLAIFY   232
NE1984          ELAEFINTIHMRIGIP----EPWGSYFMGVVSLLYGVALIAGVIVLLPSL--------VKDFLALRVGRNLKRMWLDAHNVVGITALPFHIAMAITATALSL   220
BB3604          RGGDFFYRFHFELEMA----FPWGRWLASIAGMFMLVAIISGIITHK-KI--------FTDFFTFRPRKGGQRAWMDGHNVLSVLGLPFHLMITFSGLVIFM   220
BB4741          RGGDFLYRFHFELYGM---PRIWARWLVGAATLFMLVAIVSGVITHK-KI--------FSDFFTFRPGK-GQRSWLDGHNATAVLALPFNFLITFSGLLLLM   215
WS1409          SIIPYLYRFHYTLGLP----DRYGVWLMGGVAILWLFITLSGVWLSTPNLFKEGFWHRFKPSWKIKWGAKRYRWFLDTHRALGMWLLLPLFLLALSSVALNL   227
                         *                              **                                    *                **
```

```
BCAL0117      AVPVVRPLVSLVSP----LAE-TPYTN--------P------------------------------------------------EHFPPVPPG----------   263
BCAM0492      SWGKN--FKALAAT-VNLGAP-EG-AWGGASVRSTRPGAAATAGQAPAQATAPSGHHHDSGESMPGMVMDDLPLPQTPWAVGNVPVPHSPS-----------   313
BCAM2222      GALGTVSLAGVAYP----GQPQRAFAE--------LL-----------------------------------------------GGPPPVEAG----------   233
RSp1642       GMVLAMVFNTLAFD----GRL-LEAVP--------RI--------------------------------T------------TAAGTVAAAG----------   251
NMB1721       IWGGK--FVQAWS-----QFP-AG-KWGVEPNPVSVVP------------------------THGEVLNDGKVKEVPWVLELTPMPVSGTTVGKDGINPDE   289
PA0801        VYYMQ-AGLQVVYQ----NDGERFFHE--------VQ-----------------------------------------------GSYEREEVG----------   234
PA1909        ALPI-----LMLWQG----GQL-AEMTA--------P------------------------------------------------YKDA-PPLQ----------   242
PA2403        QAILADNHVTL-TN----DGI-PPVIA--------R------------------------------------------------ADVPRAAPG----------   248
PA2465        PSQVFKPLVSLFSP----IEP-SVYEA--------R------------------------------------------------GRLPREQLG----------   253
PA3789        YWGKQ--FADVWN-----RFP-AA-MW--NDVPTSDMQ--------------------------AGSLNSASRQQVPWPLENTPLPRSQPPAADAHAEHRG   280
PA4219        GALGTLLLAPVAYP----QEPNRVFVE--------LM-----------------------------------------------GPPPPAAEG----------   230
PA4513        EWYRN--GLFKLLD----DAP-AG-----------QQA-------------------QRGKPG-GR--------------------GERP-----------   242
VCA0891       IWGGK--LVQAWN-----TFP-TYYTWGEKPQSVLT----------------------------HADLNHGSEKEMPWNLEQTPVPQSHHHGGEH------   284
VPA1524       IWGAK--MVQAWN-----TFP-TYYTWGEKPESILT----------------------------HKDLNHGSSEEMPWNLELAAVPESKDKPAHDHANMEK   291
AGR_C_3630    VWGAK--VNEWANG-SNFGYP-AG-V-------RTDVP-------------------M--------SGEHLDHIAKNSWSLEQAKVPESTAS----------   279
CC_1782       PKESEIALRPILGP----PAG-APTID-------------------------------------------------------KSITFDPGT----------   251
CC_2927       ATPI----VGYWKN----TAL-KELTA--------A------------------------------------------------YDSP-VAPG----------   239
CC_3501       FWGKE--VRQITTE-AGWGRP-KP-P-------VAEQH-------------------H-------GKPAAVEEAAGVPWALQTKTPPPSGAQGARADMDDHA   282
PP_0860       EWYRE--GLNKLLA----DAP-AA-----------GQQ-------------------QKRGEGRGRHG-------------------PQNVD-----------   245
PP_4610       YMVMP-AGIMASYG----NDTDKYFND--------LF-----------------------------------------------GRNDAPKAA----------   241
PP_4839       LWGKQ--YADLWN-----RFP-AA-MW--NDVPKSDQQ--------------------------ARELNSAHRQTVPWAMENTPMPQSGA-----------   266
SO_3407       LIYIP-WTVSTAYP----EDNQAFLKE--------LN-----------------------------------------------PARQTEKAS----------   253
XAC4036       PWYRD--GMVALLG----GPP-AI-----------RG-----------------------DKG-----------------------------------------   258
BH1854        FWGQN--FQSIATQ-SGVGYP-PS-VW-VGSAPTSTVK--------------------------TED-----IADVPWAAEKLEVPTSNVQ----------   268
BH0982        VMGEQ--INRIATS-TNTGYP-PY-ALSFRDKPESTVK--------------------------TKE----VADDVPWATEQLTVPTSS-G----------   284
SCO0863       HAGAS--IGEVQDA-LGGSTP-AV-ST-------AF------------------------------------------------PAV----------------   290
SAVERM_7350   YAGAN--IDELRTS-LGQATP-SV-SA-------AA------------------------------------------------AGE----------------   270
BC_4751       FMGNQ--IYKIASSNESLGYP-KLYMA-------------------------------------PPESKVKELPWATRKEAPPESNLN----------   255
Cgl2326       YAGSN--ITDLRTQ-LNWTQP-SV-NA-------SL------------------------------------------------TAAPQV-----------DM   278
bll7967       DWYKD--GVAWLLS----RPH-VA-----------AAK-------------------MQPK--MPAKA-------------------PRVAA-----------   256
NE0550        NEPFVW-VTQLFSP----ATR-QPQHT--------L------------------------------------------------TSIPITGIP----------   278
NE1084        SLYMP-AGIFAHYP----NKD-TYFSQ--------LL-----------------------------------------------SRPAPREET----------   264
NE1984        SHELWSLQEAVIFG----GKQ-AILDE--------RD--------------------------------N------------EPFRAPKPIG----------   255
BB3604        YMLMP-AGVLAAYD----NER-AFFDE--------MF-----------------------------------------------PAFTQTALQ----------   252
BB4741        YQLMP-AAVDAAYQ----GDTRAFFNE--------RR-----------------------------------------------AAPPPPAVA----------   248
WS1409        RNELFRPVVGLFSP----LTP-LPFEA--------L------------------------------------------------KRYPPDPKF----------   259
```

```
                                                                                                 hDhXXG
BCAL0117      ----------------SQILPRERIVEIARSAG-RDA--GIAAPPGALLFAPGMNAYAVGFFTL----GNDHGDVGLGNAWLYWNAVTGKPVAAQVP--GRG     340
BCAM0492      -------------APTPAPLPLARAIAIVAGLG--VTSGYTLALPSGA-----DGVFTASYF----------PADPKAERTIYVDQYSGAVLKDIRY--GDY     383
BCAM2222      ---GP----------WRHAPDLDALLRRDAVRR-PDFRPEAVVL-----HRWGDVNARVEIAGTTAGLPSTAVF-----ERHLYRATDGQWLADATSR--GR     309
RSp1642       ---QP-----------APLLPAAQLLATARNATGPDFTPRSIHF-----QHIGDAHAVAELRGRSTRALGDYGSL--AIRAAAGQADSGRLLGNQTAH-ARD     331
NMB1721       --------PMT----------LETVDRFAREIG--FKGRYQLNLPKGE-----DGVWTLSQDSM-----SYDMISPFADRTVHIDQYSGKILADIRF--DDY     359
PA0801        ---RP----------AGPPASIDGLIVEAGKVWGDGGAPGWISV-----HHPYDEAATVDIRRRD----ASRILD--DQRTVNFDASSGELLHAQPS----Y     308
PA1909        -----------------RLGSLDAALATARRAA-PDMEVSFVGFPGTQFSS--QHHYAVFMRG------NTPLTERL-LKPALIDAQSGELTDMR-----EM     312
PA2403        ---Q-----------KVPYIELDEAVRLVTGRI-PSLEASAVFLPGNAYSP--MFVA---GRG------WYPL---M-FQSAALNPYTGKIETTRLLS-DRT     319
PA2465        ----------------ETRLDYDRTFQLASVEA-ARL--GIAEPIGELYYSFEYN-----FFGA----GFGDHDDPMGKSWLFFHGSDGRLLGQEVA--GQG     325
PA3789        HSAQAGHAMPM-DMPMPAGIPLQRVVDIARERG--VAAGYGIALPSGM-----EGVYTISVF----------PDDPRHDATLHIDQYSGKVLADVRW--QDY     362
PA4219        ---RP----------LASRIDLDRLLAGDAVRA-PGFVAQRLSL-----SHAGDVAGSVEIAGIRRGLPSTANF-----ERHRYRLADGTLLGERSSA--QR     306
PA4513        ------------ADAAPLVVDYAAVWNSIRQAGGDRLQAYNLRLPPAG-----GQPATVFYR-------LTDAPHERAFNTLTLDPANGQVKSDQRY--SDR     318
VCA0891       ----------E-MVAVNPQFGIDQVIAQAKALG--F-TQYRVAFPRGE-----TGVYTVSANTM-----AGDIVDPRDDRTAHFDQYSGALLTEVTW--QDY     360
VPA1524       --------GAS-YTASHRALSIDDIILKAEMMG--F-TSYKIFLPRSD-----NGVYTVAANSM-----GGDISDPRQDRTSHFDQYSGRLLVDVTW--QDY     369
AGR_C_3630    --------------ATGQPIGINAAIARFDGLG--LAAGYAVALPTKP-----TGVYSGSVY----------PDDLSKQRVVHLDQYSGEPLIDMRY--ADY     348
CC_1782       ----------------R--ISVDQAVAIAQGAL-PGARLAWIETPSTD-----SGVYRLRLQT------PGDPSRRFPHSFVWITPATGEVLAMTDA--RQA     321
CC_2927       -----------------QRSSLQAAVDKAKQAL-PDKDLQFVAFPGSDYST--DHHYAVFFHG------KTPLTTHL-TTPALIDARTGELAAIA-----PT     309
CC_3501       -------HHMM-MMDAPAPLDANAIVAKARAAG--LTGGFTLSLPKAP-----GGTWTAAQM----------SDQVEQTRAVYLDGGDGRVLADIGY--RDF     357
PP_0860       ------------RNAPPLVVDYDAIWANLKDAAGPGLSTYNLRLPPAG-----GQPANLFYL-------LDNAAHPRAFNNLVLDPATGQVKKHDRY--TDK     321
PP_4610       ---QV----------ATPLVALPSLYAKVQELQ-PGARVGNIQV-----QNPGDSNARVTFTQSA----ADHVAYRRS-ANWTFDGASGALLSQG----KPE     315
PP_4839       HAEHAGHHMMS-DMPAAPQVSVQQVEDIATSRQ--VELGYSITLPTTA-----DGVFTIAVF----------ADDPRNDATLHVDQYTGKVLADVRW--HDY     348
SO_3407       ---GI----------KAEQVRLSQLLPRVQAQW-GDAPIKQVTV-----SNPKDQNSQVTFYQNT----GKDVTD--ESTLLVFSGVTGELKYASP---HEV     327
XAC4036       -------------DSRPATVDLARVQRTLDGIPATRSAALDLRIPTRA-----GQPLNVRFL-------PDNPAHDRAYNSLDIAPDSGALLQRQDY--ALL     333
BH1854        ---------------GFLPLAVEEVVTIANRVG--MHPTYTIYLPREQ-----DSVYTVSAY----------PPRAQDEATIHIDQYTGAVLADYRY--DHY     336
BH0982        ---------------GQVPISLEDVTYLAETRE--VNKPYTISMPEGD-----TGVYTIASS----------HTKPGDNATIHVDQYSGAILSDVRF--HDY     352
SCO0863       ---GSGKEDA----GGTRDVGIDAAVAAAQDAG--LRGVLVVTPPSEA-----GTAYVVKENT---------RSWPVRQDSVAVAPATGEVTQTLRF--DDF     367
SAVERM_7350   ---HAGHDAAAGTGGTEHGVGLDKILAAARAKG--LGDPVEIVPPADA-----SSTYVVKQVQ---------RSWPEKQDSVAVDPATGEVTDTLRF--ADY     351
BC_4751       ---------------EPKAIS---VDELQKGIE--IKKPYVISLPADP-----KGVFTVSKSSG--SGITGMHVAPNEEITAYFDQYSGELISKTDY--RDY     328
Cgl2326       HDEHAGHHMH--MESATSGSGSIDLVAATAISE--LRTPLTITPPAQD-----GLAWTATENR---------DAYRFTTDTIAVDGDTGMLTNRLNS--TDW     360
bll7967       ------------VSEPARPIGFDQAWTTFQREEGDRFSRALLTLPAGP-----GTAIRIRSW-------GKDSTLEATRDEFRVDAATGQLVAADRY--ADK     332
NE0550        ----------------S--IGAEHAWAIATEHY-PDGKFGGMFMPGNA-----EGVYIVTQKH------VPKLSAFWSERQIAIDQYSGEILDVRAPD-ARR     349
NE1084        ---HI----------DAQVASLDKLLLT-AETE-LGRRASFVSV-----NHPGDSSASVTVFGLFDEEENEKYLLPPGSGNVIFDGITGETLDIQMSGDHRG     346
NE1984        ---EA----------GA-MMAPSQLLQRLKAQV-PDFEPKVMIF-----KNIGDKAASVRVAGAERGYVGDTHL-----GGVMLSAVTGELLKDSTRP-SRQ     331
BB3604        ---AN----------PAPLAPLAGLAARARAEW-DGGRPGRIVV-----NNPGDASATVSVTRST----SERIAYGRMAPTITFDGVTGQLRSQD----AKE     327
BB4741        ---LRGQ-----GMAAPSLPALQAMLDDAARLW-PASPVGTITV-----DRQGGSANWVELRESH----GQSLTDRGVARRAVYDVATGQRLQVPPA--PRP     330
WS1409        ----------------RPKVEFDEALERARVAL-AGE--GIELEPRSIFYLGGFRSYMVLF--------GPKHPLGYGTPRIVIHAESGEYVSRYIP--GRG     332
```

```
                                                   PepSY_TM4
BCAL0117      SAGDLFMQAQFPLHSGRIA------GVAGRVAVSVLGIVIAMLSVTGVCIWVKKRGARGRAAR--------SARP--AVP----------ASSRAAR*          411
BCAM0492      GAVSKAVSYGTSLHMGRYF------GLANQIICAVLSLGLAAMAVTGTVMWWKRRPAG----T--LGAPSRERGTPPMRG--------WIAGLVLLGIVFPLMG   467
BCAM2222      GFWLRAFIAVQPLHFAQYGWVGAA-GDGLRVLHFLMGLAACALCATGLHLWIERRRAQRE----------RSADV--LAA----------AAV---GVCGGLVL   387
RSp1642       G-NHAIYSVVYGLHFGTYG------DVALRLAYFVMGMAGAFVFYSGNLLWIESRRKQRN------PAQPRVHRL--LAQ----------ATV---GICIGCCA   407
NMB1721       NPFGKFMAASIALHMGTL-------GWWSVLANVLFCLAVIFIGISGCVMWWKRRPTGA--VG--IVPPAQKVKLPVWWM--------MALPLLAIALLFPTSL   444
PA0801        APGYATYGWLTGLHMIQWG------GQLVRWMYLLLGLSGAMMIFGGLQVWLAKREVRGS----------RGVGL--VRA----------LNL---AVCGGLSL   381
PA1909        PLYVKTLLLSQPLHFGDYG------GMPLKIVWALLDLVSIVILASGLYLWLGRRKTPLEKRL------AELPDG--S---------------LA---TEGKA*   383
PA2403        PLQF-VTESMRPLHTGDFG------GIWVKLIWFVFGLLLSMMVLSGLLIWSKRTAQATAALVKRGKRPARQPKP--AKP----------APVMANQTAEGQP*   403
PA2465        SWGERFYRLQYPIHGGRIA------GLPGRIAIAALGLAIAGLSLTGVYIWWRKRRARHWNGR*                                            382
PA3789        NAVARSVQMGVVLHEGKLF------GLGNQLLMLAVCLAILLSSASGLWIWWKRKPAG----R--LGVPPLRHELPRWKT--------GIAIMLALGVAFPLVG   446
PA4219        GFWLRAFIAVQPLHFAQYQWLGPGWSAALRGLHLAMGLGACLLCASGLYLWLQRRASAPD----------ARVRL--LQR----------LSQ---GFCAGLVA   385
PA4513        SFGSQLLASVYALHVGSYF------GMAGRILMMLASLAMPLFFITGWLLYLDRRRKKR------AVRASRADLG-------------------------GSAG   385
VCA0891       SPFAKAMAAGISLHQGDL-------SVWNKIANVLFCLAFILISVTGVVMWWLRRPTGQ--AR--LGVPPRFEQDGVWKA--------GLATLLVIGVAFPLAG   445
VPA1524       SWFAKLMAAGVSLHQGDV-------SIINKALNVLFCLAFILIAISGVVMWWLRRPSRS--AS--LGAPPQFQHDGVWKL--------GLATLVAICAAFPMGG   454
AGR_C_3630    GPLGKALEWGINVHMGQQF------GLANQIVLLAACFGIVLLAVSAGIMWWKRRPRG----S--LGVPPLPQDKRVLRG--------LLAMLAIGGILFPLVG   432
CC_1782       GAHDVLLNWTHPLHDASAG------GLPARLLAVLVGLAPSALLILGFMRWRQRLRPQERKLS--------DHS*                                 381
CC_2927       PWYVKTLTLSQPLHFGDYG------GMGLKILWALLDIATIVILASGLYLWLAKRKVAR*                                                362
CC_3501       GPAAQAIEWGIAVHEGRQF------GLVNTLVMLAGCVAVWLMGVSAIVMWWKRRPQGRPEGR--LAAPARPAHRGAYVG--------LLAIVLPLAVLYPLVG   445
PP_0860       SFKAQLLQSVYALHVGEYF------GLPGRIIVTIASLTMPLFFVTGWLLYLDRRRKKR------QVRAARGNVA-------------------------NS--   386
PP_4610       SGVMMTAFSFAGLHMGNFA------GPWLRWLYFFFGVAGTAVIGTGLVMWLGKRQLKHAK----NGHMPGELRL--VEV----------LNI---ASMSGLLL   394
PP_4839       SPVARATELGVMLHEGKMF------GALNQIIILLVCLMILLGSVSGLVMWWKRRPEG----G--LGVPPLRHDLPRWKA--------AVAVMLVLGVMFPLVG   432
SO_3407       SGAVVTYDTMMSLHTARFA------APLLRILFFFCGLLGCAMVATGTLMWAIRLRQKQQKVIDKGEKAGLGLRL--VEG----------LNF---MFILGLPL   410
XAC4036       PRGQQLAVSMFPLHSGSFF------GLPGRIVVMLASLGMSVFFVTGWMLYLDRRRKKR------ALRAARKVLQ-------------------------GAAP   400
BH1854        GSLAKAVALGITIHKGTQF------GWMNQWISLLVCIGMIGVALSGYYLWWKRKPKQ----G--LGAPKAPSVEKMKRF--------LF-LLIVLGIIFPLVG   419
BH0982        GMMAKGITIGIALHEGRLF------GLPNQILGLITCLGLIGLIVSSWVMWKKRKPQR----S--LGAPTQVKDRKVSRA--------VFVIMMVMGIIMPLVG   436
SCO0863       PVLAKLTSWGINAHMGLLF------GLVNQIVLALLAIGLIAMILWGYRMWWLRRPTRSEGFA--LGRA---PARGAWRRIPGRVLAPALVLAAVIGYYLPLFG   460
SAVERM_7350   PLLAKLTRWGIDAHTGVLF------GLVNQIALMALALSLVLLIVWGYRMWWQRGRA----SA--FGRP---IPRGAWQQVPPQILVPCMAVIAVLGYFVPLLG   440
BC_4751       GLLAQWFTYGIPLHEGHLF------GWPNKILCLLTTLSLLLLIYYGIKMWLARKPKG----K--LAAPPKQRDKKSIFV--------FFIMMVILGAVMPLFG   412
Cgl2326       PLAAQASAWLIQLHMGTLF------GLPNQVVLGLLAASIIVMIGLGYWMLWQHRPREGWPSA--PKRAG--FEKPTWGTIAL------GVVVIAYGLLAPLFA   448
bll7967       TFGEKIIAAVYDIHRGAIL------GWPGKLAFMIAAALMPLFAITGLLLYLSRRRLRR------PAQPPLGLLV-------------------------PGE*   398
NE0550        SAGETFLEWQWPLHSGQAF------GWPGRILIFLCGLACPVIYATGVIRWLQKRRVKVRSPR--------RPIR*                                410
NE1084        GEAQAVQRVMGTLHFARFG------GDTIKWLYFISGLAGAIMMATGSILFMVKRRQKALNEF--GSHTRRVYRL--IET----------LNV---AVIAGLCI   427
NE1984        DPDRRASETFYGLHSGQYE------GATITWAYFFLGFSGAWLFYSGNLLWIETRRKKACKGGE-LPEQRRDTYW--LGA----------GTV---GVCLGCVA   413
BB3604        SAVAATAGTIVGLHLGLFA------EPVLRWLYFLVSLAGTAMVGTGLALWIAKRRQKAA------GRESFALRL--VDA----------LNA---GSIAGLCV   404
BB4741        SAASAVYNVFTSLHLGRFA------DPGVRLLLLVSGLVGSFMVASGMVLWVVKRLPERRK----TGITPRGHRV--VEI----------LNV---AGLGGLCL   409
WS1409        SVGDRFTHLQFPLHSGQIA------GIGGRIFVAILGLVITWACISGLYLWWKKNPFSRISLF--------NLKF--KKE----------QS*             398
                           *                               **
```

Fig. S8. Amino acid sequence alignment of 36 representative PepSY_TM domain-containing proteins (PiuB-type proteins). Amino acid sequences of PepSY_TM domain-containing proteins (PiuB-type proteins) from various bacterial species were aligned by Clustal Omega and identical or similar amino acids were highlighted using BOXSHADE. White font with black shading indicates identical residue at the corresponding position in ~50% of sequences and white font with grey shading indicates similar residues at the corresponding position in ~50% of sequences. Many proteins containing PepSY-associated TM regions contain one or more additional domains at the C-terminus, but for clarity these additional domains are not shown. The location of the two PepSY_TM domains and hDhXXG motifs are indicated above the alignment. The invariant histidine that is located N-terminal to each PepSY_TM domain is indicated with an asterisk and the characteristic (S/T)G motif located near the C-terminus of each PepSY_TM domain is indicated with tandem asterisks below the alignment. A conserved sequence located within each PepSY domain is shown enclosed in a red box. This sequence corresponds to h(Y/F)hDPY(S/T)(G/A)XhhG in the N-terminal PepSY domain and hXhDhX(S/T)GXhh in the C-terminal PepSY domain, where h = amino acid with an aliphatic side-chain (i.e. I, L or V). Both motifs contain the previously identified conserved hDhXXG motif (22). Amino acids corresponding to the N- and C-terminal PepSY domains of FoxB (PA2465) are shown in dark orange and magenta font, respectively, and the cytoplasmic loop connecting the C-terminal PepSY_TM domain of the N-terminal PepSY domain with the N-terminal PepSY_TM domain of the C-terminal PepSY domain is shown in blue font (based on 23). BCAL0117 and BCAM2222 correspond to *B. cenocepacia* FhuB and FptC, respectively; PA2403, PA2465, PA4219 and PA4513 correspond to *P. aeruginosa* FpvG, FoxB, FptC and PiuB, respectively.

FptX

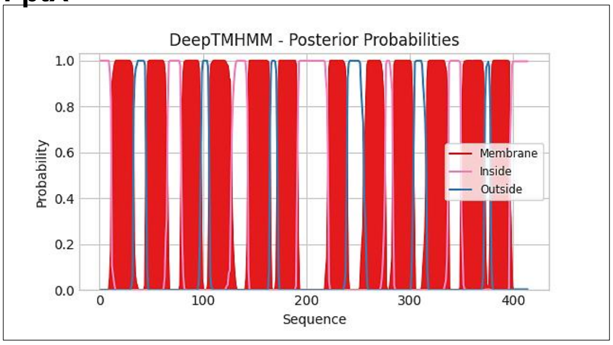

FiuB

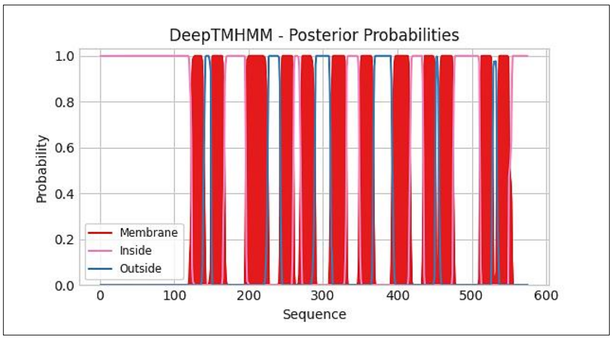

FhuB

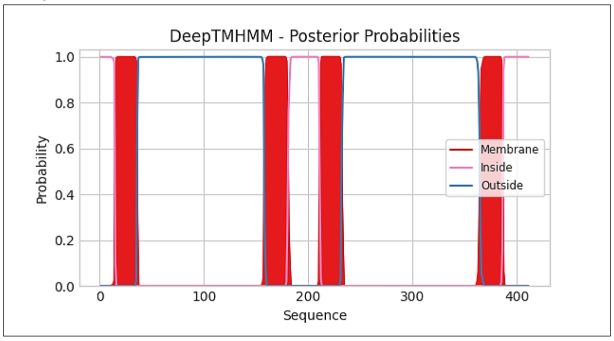

FoxB

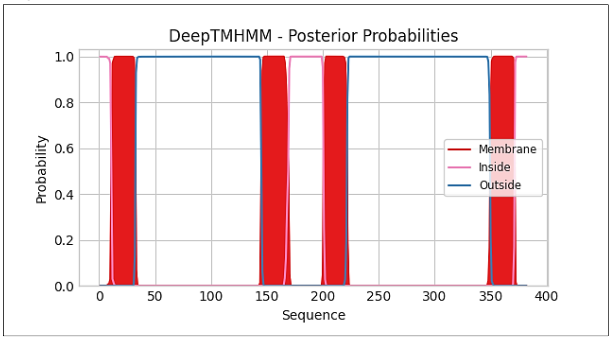

FpvG

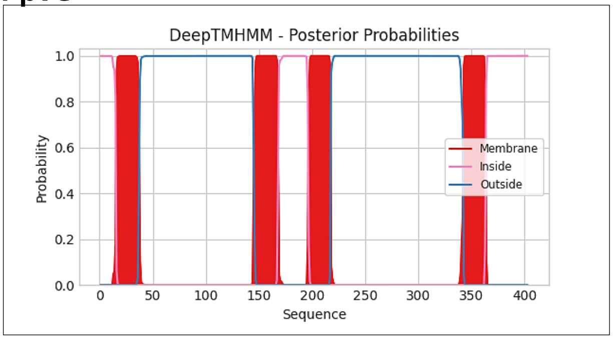

Transmembrane   Inner membrane   Outer membrane

Fig. S9. TMD analysis of the FptX, FiuB, FoxB and FpvG proteins of *P. aeruginosa* PAO1 and the *B. cenocepacia* H111 FhuB protein. The number and location of transmembrane segments in *B. cenocepacia* H111 FhuB (BCAL0117/I35_RS00625) and the FptX (PA4218), FiuB (PA0476), FoxB (PA2465) and FpvG (PA2403) proteins of *P. aeruginosa* PAO1 were predicted using DeepTMHMM version 1.0.24.

References

1. Darling P, Chan M, Cox AD, Sokol PA. Siderophore production by cystic fibrosis isolates of *Burkholderia cepacia*. Infection and Immunity. 1998;66(2):874-7.
2. Farmer KL, Thomas MS. Isolation and characterization of *Burkholderia cenocepacia* mutants deficient in pyochelin production: pyochelin biosynthesis is sensitive to sulfur availability. Journal of Bacteriology. 2004;186(2):270-7.
3. Spiewak HL, Shastri S, Zhang L, Schwager S, Eberl L, Vergunst AC, et al. *Burkholderia cenocepacia* utilizes a type VI secretion system for bacterial competition. MicrobiologyOpen. 2019;8(7).
4. Asghar AH, Shastri S, Dave E, Wowk I, Agnoli K, Cook AM, et al. The *pobA* gene of *Burkholderia cenocepacia* encodes a group I Sfp-type phosphopantetheinyltransferase required for biosynthesis of the siderophores ornibactin and pyochelin. Microbiology. 2011;157(2):349-61.
5. Carlier A, Agnoli K, Pessi G, Suppiger A, Jenul C, Schmid N, et al. Genome sequence of *Burkholderia cenocepacia* H111, a cystic fibrosis airway isolate. Genome Announcements. 2014;2(2):298-314.
6. Ankenbauer R, Sriyosachati S, Cox CD. Effects of siderophores on the growth of *Pseudomonas aeruginosa* in human serum and transferrin. Infection and Immunity. 1985;49(1):132-40.
7. Yanisch-Perron C, Vieira J, Messing J. Improved M13 phage cloning vectors and host strains: nucleotide sequences of the M13mpl8 and pUC19 vectors. Gene. 1985;33(1):103-19.
8. Ferrieres L, Hémery G, Nham T, Guérout A-M, Mazel D, Beloin C, et al. Silent mischief: bacteriophage Mu insertions contaminate products of *Escherichia coli* random mutagenesis performed using suicidal transposon delivery plasmids mobilized by broad-host-range RP4 conjugative machinery. Journal of Bacteriology. 2010;192(24):6418-27.
9. Simon R, Priefer U, Pühler A. A broad host range mobilization system for in vivo genetic engineering: transposon mutagenesis in Gram-negative bacteria. Nature Biotechnology. 1983;1(9):784-91.
10. Herrero M, de Lorenzo V, Timmis KN. Transposon vectors containing non-antibiotic resistance selection markers for cloning and stable chromosomal insertion of foreign genes in gram-negative bacteria. Journal of Bacteriology. 1990;172(11):6557-67.
11. Jackson SA, Fellows BJ, Fineran PC. Complete genome sequences of the *Escherichia coli* donor strains ST18 and MFD pir. Microbiology Resource Announcements. 2020;9(45):1014-20.
12. Marinus MG. Location of DNA methylation genes on the *Escherichia coli* K-12 genetic map. Molecular and General Genetics 1973;127(1):47-55.
13. Casadaban MJ, Cohen SN. Analysis of gene control signals by DNA fusion and cloning in *Escherichia coli*. Journal of Molecular Biology. 1980;138(2):179-207.
14. Shastri S, Spiewak HL, Sofoluwe A, Eidsvaag VA, Asghar AH, Pereira T, et al. An efficient system for the generation of marked genetic mutants in members of the genus *Burkholderia*. Plasmid. 2017;89:49-56.
15. Dix SR, Owen HJ, Sun R, Ahmad A, Shastri S, Spiewak HL, et al. Structural insights into the function of type VI secretion system TssA subunits. Nature Communications. 2018;9(1):4765.
16. Kovach ME, Elzer PH, Hill DS, Robertson GT, Farris MA, Roop II RM, et al. Four new derivatives of the broad-host-range cloning vector pBBR1MCS, carrying different antibiotic-resistance cassettes. Gene. 1995;166(1):175-6.
17. Kovach M, Phillips R, Elzer P, Roop 2nd R, Peterson K. pBBR1MCS: a broad-host-range cloning vector. Biotechniques. 1994;16(5):800-2.
18. Khan SR, Gaines J, Roop RM, Farrand SK. Broad-Host-Range expression vectors with tightly regulated promoters and their use to examine the influence of *traR* and *traM* expression on Ti plasmid quorum sensing. Applied and Environmental Microbiology. 2008;74(16):5053-62.

19. Fazli M, Harrison JJ, Gambino M, Givskov M, Tolker-Nielsen T. In-frame and unmarked gene deletions in *Burkholderia cenocepacia* via an allelic exchange system compatible with gateway technology. Applied Environmental Microbiology Reports. 2015;81(11):3623-30.
20. Mannaa M, Park I, Seo Y-S. Genomic features and insights into the taxonomy, virulence, and benevolence of plant-associated *Burkholderia* species. International Journal of Molecular Sciences. 2018;20(1):121.
21. Peacock RS, Weljie AM, Howard SP, Price FD, Vogel HJ. The solution structure of the C-terminal domain of TonB and interaction studies with TonB box peptides. Journal of Molecular Biology. 2005;345(5):1185-97.
22. Yeats C, Rawlings ND, Bateman A. The PepSY domain: a regulator of peptidase activity in the microbial environment? Trends in Biochemical Sciences. 2004;29(4):169-72.
23. Josts I, Veith K, Normant V, Schalk IJ, Tidow H. Structural insights into a novel family of integral membrane siderophore reductases. Proceedings of the National Academy of Sciences. 2021;118(34).
